# Computational Structural Analysis: Multiple Proteins Bound to DNA

**DOI:** 10.1371/journal.pone.0003243

**Published:** 2008-09-19

**Authors:** Andrija Tomovic, Edward J. Oakeley

**Affiliations:** Friedrich Miescher Institute for Biomedical Research, Novartis Research Foundation, Basel, Switzerland; Center for Genomic Regulation, Spain

## Abstract

**Background:**

With increasing numbers of crystal structures of protein∶DNA and protein∶protein∶DNA complexes publically available, it is now possible to extract sufficient structural, physical-chemical and thermodynamic parameters to make general observations and predictions about their interactions. In particular, the properties of macromolecular assemblies of multiple proteins bound to DNA have not previously been investigated in detail.

**Methodology/Principal Findings:**

We have performed computational structural analyses on macromolecular assemblies of multiple proteins bound to DNA using a variety of different computational tools: PISA; PROMOTIF; X3DNA; ReadOut; DDNA and DCOMPLEX. Additionally, we have developed and employed an algorithm for approximate collision detection and overlapping volume estimation of two macromolecules. An implementation of this algorithm is available at http://promoterplot.fmi.ch/Collision1/. The results obtained are compared with structural, physical-chemical and thermodynamic parameters from protein∶protein and single protein∶DNA complexes. Many of interface properties of multiple protein∶DNA complexes were found to be very similar to those observed in binary protein∶DNA and protein∶protein complexes. However, the conformational change of the DNA upon protein binding is significantly higher when multiple proteins bind to it than is observed when single proteins bind. The water mediated contacts are less important (found in less quantity) between the interfaces of components in ternary (protein∶protein∶DNA) complexes than in those of binary complexes (protein∶protein and protein∶DNA).The thermodynamic stability of ternary complexes is also higher than in the binary interactions. Greater specificity and affinity of multiple proteins binding to DNA in comparison with binary protein-DNA interactions were observed. However, protein-protein binding affinities are stronger in complexes without the presence of DNA.

**Conclusions/Significance:**

Our results indicate that the interface properties: interface area; number of interface residues/atoms and hydrogen bonds; and the distribution of interface residues, hydrogen bonds, van der Walls contacts and secondary structure motifs are independent of whether or not a protein is in a binary or ternary complex with DNA. However, changes in the shape of the DNA reduce the off-rate of the proteins which greatly enhances the stability and specificity of ternary complexes compared to binary ones.

## Introduction

DNA-binding proteins are important for the regulation of many crucial cellular processes (including transcription, recombination, and replication). The number of DNA-binding proteins known is very small compared to the number of regulatory controls they must provide within the nucleus. The problem is solved, at least in part, by the construction of higher-order regulatory complexes composed of multiple proteins. Structural analyses of such complexes may enable us to model the forces driving their assembly and stability which in turn may help us to understand these processes better. Such an understanding may help in predicting DNA-binding specificities. Transcription factors, a large subclass of DNA-binding proteins, are known to act cooperatively in the regulation of gene expression [1]–[7]. Their complexes can include both DNA and non-DNA-binding factors. The DNA-binding factors may be located either remotely (at some distance) or adjacent (with direct contacts) to their promoters [5].

Thanks to a large number of recent X-ray and NMR structures of protein∶protein, protein∶DNA, and protein∶RNA complexes, a lot of valuable information about the general features of such complexes has been discovered [8]–[23]. These results indicate that it is very difficult to find universally characteristic rules which can describe all protein-protein, protein-DNA, and protein-RNA interactions. However, some general principles have been deduced. For example, Lys or Arg pair preferentially with any nucleotide in both protein∶DNA and protein∶RNA complexes [16]; two-thirds of all protein-DNA interactions involve van der Waals contacts, compared to about one-sixth involving hydrogen bonds [18]; on average protein-protein interface has approximately the same non-polar character as the protein surface as a whole and carries somewhat fewer charged groups (however, some interfaces are significantly more polar and others more non-polar than the average) [17].

The current work comprises a structural analysis of macromolecular assemblies where several proteins are bound to DNA, using data from the Protein Data Bank (PDB) [24]. We analyzed the following chemical and physical properties: the size of interfaces between any two components; the number of residues/atoms involved in contacts between components; residue interface propensities and chemical composition; water-mediated contacts in interfaces; secondary structure motifs in interfaces; and interactions between amino acid side chains either with the DNA or with another protein in the complex. Some of these interface properties for ternary/quaternary complexes (i.e. complexes involving two/three proteins bound to DNA) have been compared with those obtained from binary complexes. One possible hypothesis why the above-mentioned protein-DNA and protein-protein interface properties are expected to depend on the number of proteins in a complex is that when two proteins are free (not bound to DNA) they are more able to find the best patches (on both proteins) to produce the most stable complexes possible, with the highest affinity between components. However, when one protein is bound to DNA then there is a spatial limitation in the movements that are possible in order to find the best interface patches (on both proteins) in order to make stable complexes. This is one possible explanation why protein-protein interface properties can be expected to be different in protein∶protein and in protein∶protein∶DNA complexes. A possible implication is that (if properties are similar or the same) actually two DNA-binding proteins bind first to each other and then bind to DNA together (as a complex). A similar hypothesis can be derived for protein-DNA interfaces in protein∶DNA and in protein∶{protein+}∶DNA complexes. One might suppose that these interfaces can be different, because when one protein binds to DNA there is a higher degree of freedom (rotational, translational) than when one protein should bind to a previously-made protein∶DNA complex. This is useful (from a theoretical point of view) for better understanding protein-DNA interactions which frequently involve complexes of multiple proteins. In addition, this can be useful (from a practical point of view) for the possible modelling of such complexes (their prediction, prediction of order of processes, modelling cis-regulatory modules, etc). In addition the nature of protein-protein interface and protein-DNA interface might be different that there is no any competition between them. This aspect can be also considered with this kind of analysis performed in this paper. In this work we have also calculated and compared, the conformational change of DNA in binary complexes (i.e. single protein-DNA complexes) and ternary/quaternary complexes (protein-protein-DNA/protein-protein-protein-DNA). Next, we analyzed protein-protein and protein-DNA energy binding affinity in protein-protein, single protein-DNA and multiple proteins-DNA complexes using several different tools. In addition, we analyzed and compared the thermodynamic stabilities of these complexes. We have provided an algorithm, and its web-based implementation, for calculating overlapping interface volumes and the number of interface atoms in collision between any two components (macromolecules) from a 3D complex stored in a pdb file.

## Results and Discussion

We have performed computational structural analysis and present herewith some general features we have observed about macromolecular assemblies of multiple proteins bound to DNA. The following tools were used in our analysis: PISA [25], [26]; PROMOTIF [27]; X3DNA [28]; ReadOut [29]; DDNA [30] and DCOMPLEX [31]. Additionally, we have developed and used an algorithm for collision detection and overlapping volume of two macromolecules. Web-base implementation of the algorithm is freely available from http://promoterplot.fmi.ch/Collision1/ (see Materials and Methods for details). All data sets, used in this study, are from the PDB database (see Materials and Methods for a definition of data sets used in this study).

### Physical properties of interfaces

Do physical properties of interfaces depend on the number of units in macromolecular assemblies? Are there any differences in physical properties of interfaces among protein∶protein∶DNA, protein∶DNA and protein∶protein complexes? In order to answer these questions, we performed analysis of physical interface properties of different macromolecular assemblies.

The number of interfaces in the dataset MutliProteins∶DNA together with their structural characteristics is summarized in Table 1.

**Table 1:** Descriptive statistics of interfaces.

Interface type	Number of interfaces	Average size of interface (Å^2^)±SE	Average number of interface residues*±SE	Average number of interface atoms*±SE	Average number of intermolecular H-bonds±SE	Average number of intermolecular salt bridges±SE
Protein-protein	52	929.84±179.4	49.5±8.4	190.9±36.0	9.36±3.7	4.08±0.7
DNA-protein	87	1002.3±56.5	52.2±2.9	222.2±12.5	18.0±1.1	0.0±0.0

Descriptive statistics of protein-protein and protein-DNA interfaces of complexes from group-MultiProteins∶DNA.

* For both components together in interface.

A detailed list of 52 protein-protein and 87 protein-DNA interfaces is given in Table S1. These values represent the sample sizes for the following hypothesis tests between protein-protein and protein-DNA interactions: There was no significant difference in average interface surface sizes (student's t-test, p-value = 0.69); nor the average number of interface residues (student's t-test, p-value = 0.76) nor the average number of atoms (p-value = 0.41). Based on this we can conclude that protein-protein and protein-DNA interfaces have similar average sizes and numbers of residues/atoms involved in their interactions in protein∶protein∶DNA complexes. La Conte et al. [17] found that most protein-protein interface areas are in the range of 1200–2000 Å^2^. They consider the total area on both components (without dividing by 2 to make the average area) as shown in formula (2). The protein-protein and protein-DNA interface areas for protein∶protein∶DNA complexes are also to this range (Table 1). The average area of protein-protein interfaces of complexes in the group-MultiProteins∶DNA and the average area of protein-protein interfaces of complexes in the group-Protein∶Protein we observe was comparable to those reported by Chakrabarti and Janin [9]. The DNA interface area sizes reported in Table 1 are comparable with those reported in studies considering only single protein-DNA complexes [15], [21]. The number of residues/atoms in protein-protein interfaces in this study was also comparable to previous studies [9], [17]. The situation is similar if we compare protein-DNA interfaces of protein∶protein∶DNA complexes with protein-DNA interfaces of protein∶DNA complexes [15], [21].

Based on this we can conclude that average interface size and the average number of interfaces residues/atoms between two macromolecules (DNA, protein) in any kind of complex (protein∶protein, protein∶DNA, protein∶protein∶DNA) are approximately the same. In addition, it appears that these physical properties are not influenced by the number of subunits in the complex.

### Distribution of hydrogen bonds in interfaces

The purpose of this section was to investigate differences in distributions of hydrogen bonds between interfaces of macromolecular assemblies. There is a statistically significant difference in the average number of intermolecular hydrogen bonds (H-bonds) between protein-protein and DNA-protein interfaces (student's t-test, p-value<0.0001). The number of H-bonds observed in previous protein-protein studies (mean 10.1±0.5) [17] is comparable to those reported in this study for group-MultiProteins∶DNA (Table 1). The situation is similar if we compare protein-protein-DNA verses protein-DNA interfaces [15], [21]. The small observed variations are due to small variations in the interface areas as the number of hydrogen bonds is dependent on this area.

In Table S2 we report the numbers of hydrogen bonds observed between the 20 amino acids and the four bases or the backbone of the DNA for the complexes listed in the group-MutliProteins∶DNA. We found that H-bond pairs were significantly different from random (Fisher's test, p<10^−6^). The most favoured amino acid-DNA base H-bond is ARG-G. In Figure S1 we report the distribution of H-bonds between the DNA bases and the bound proteins in group-MutliProteins∶DNA. 65.69% of all H-bonds where between protein side chains and the DNA backbone (Figure S1). Those H-bonds are not expected to confer specificity of binding but rather assist in complex stability. Most amino acids involved in H-bonds between the proteins and DNA (complex from group-MultiProteins∶DNA) are positively charged, presumably because of the negative charge of DNA (Figure S2). For the H-bonds at the protein-protein interfaces, the situation is different: negative and positively charged amino acids have an approximately equal frequency due to the need to pair charges in electrostatic interactions between donator and acceptor sites in the two proteins. Very similar distributions of H-bonds are found in groups –SingleSameProtein∶DNA and –SubSetMultiProteins∶DNA (Table S3, Table S4, Figure S3, Figure S4).

Most H-bonds (53.3%) are made with phosphate groups of the DNA at the protein∶DNA interfaces. Very few H-bonds (12%) are made with deoxyribose (Figure S1). This situation is the same as that reported by Lejeune et al. [16] and Luscombe et al. [18] for protein-DNA interactions. The distribution of H-bonds between the participating amino acids and the DNA is given in Table S2. Entries in Table S2 that diverge from the expected distribution (favoured amino acid-base H-bonds) are also similar to those observed by Luscombe et al. [18].

### Distributions of interface residues

In this section we present results about distributions of interface residues. We investigate if distributions of interface residues dependent on the number of units in the complex and if there are any differences in residue distributions between binary and ternary complexes (protein∶protein∶DNA, protein∶DNA, protein∶protein). The amino-acid propensities for the protein-protein and protein-DNA interfaces for complexes from the group-MultiProteins∶DNA are shown in Figure S5. For protein-DNA interfaces, ARG and LYS have the highest propensity values (>1.2), which indicates that they occur greater than 20% higher frequently in the interfaces than in the whole dataset. On other hand, many amino acids (ALA, ASP, CYS, GLN, GLU, ILE, LEU, MET, PHE, PRO, and VAL) are disfavoured in the interactions sites. For protein-protein interfaces, the situation is different and MET is the most favoured residue at interaction sites. In Figure S6 we report the distribution of amino acids involved in protein-protein and protein-DNA interfaces in the complexes from the group-MultiProteins∶DNA. Aliphatic amino acids are dominant in protein-protein interactions, while positively charged amino acids are the most involved in protein-DNA interactions. Those two distributions are significantly different, with a p-value<0.0001 (Chi-square multinomial test). The complexes in group-MutliProteins∶DNA have a number of van der Waals interactions between the amino acids in the proteins and either the DNA bases or backbone that is significantly different from random (Table S5, Fisher's p-value<5×10^−6^). In order to determine which of the pairings are different from expected, we performed individual Fisher's tests on each pair. The distributions of interface residues for protein-DNA interfaces of the complexes in the groups-SubSetMultiProteins∶DNA and –SingleSameProtein∶DNA are reported in Table S6 and Table S7.

Protein-protein interfaces are more hydrophobic than protein-DNA interfaces (they contain significantly more aliphatic amino acids, see Figure S6 for details). Protein-protein interfaces have many more negatively charged amino acids and far fewer positively charged amino acids than protein-DNA interfaces. All these interface parameters give an indication of the overall polar nature of protein-DNA interfaces. Given that the DNA molecule surface is negatively charged, it is perhaps not surprising that it favours positively charged protein surface patches.

The frequency distributions of amino acids in protein-DNA interaction sites in this study from the group-MultiProteins∶DNA are similar to those reported by Lejeune [16] (Figure S5 and Figure S6).

### Distribution of interface structural motifs

**Figure 1:**
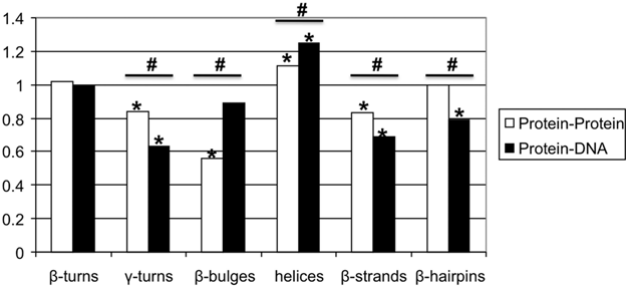
Secondary structure motif propensities. Secondary structure motif propensities for protein-protein and protein-DNA interfaces. Propensity values which are significantly different from 1 (either above or below), evaluated by the statistical bootstrapping method, are marked with “*”. Significant statistical differences between motif propensities of protein-protein and protein-DNA interfaces are marked with “#”.

We investigated if the distributions of structural motifs in interfaces of components in ternary (protein∶protein∶DNA) complexes are different from those in binary complexes (protein∶protein and protein∶DNA). In order to answer on this question we calculate the propensity values for protein-protein and protein-DNA secondary structure motifs from the group-MultiProteins∶DNA (shown in Figure 1). The most favoured protein-DNA interface motif in is the helix, and the least favoured motifs are γ-turns, β-strands, and β-hairpins. At protein-protein interfaces, the least favoured secondary structure motif is the β-bulge. The distributions of secondary structure motifs between protein-protein and protein-DNA interfaces are significant different (Chi-square multinomial goodness-of-fit test, p-value<0.01). For protein-DNA interfaces, the dominant structural motif is the helix. This result is consistent with the observation that many DNA binding sites on proteins are comprised of helix motifs [32]. The distribution of secondary structure motifs in protein-protein interfaces for the complexes used in this study (group-MultiProteins∶DNA, Figure 1) is similar to that observed by Guharoy and Chakrabarti [33] who observed that the contribution of β-strands is lower than that of helixes and that non-regular structural motifs appear in large numbers.

All previous results (from this and previous subsections) can be summarized in the form:
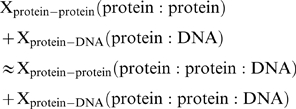
(1)where X_protein-protein_ (C) and X_protein-DNA_ (C) represent one of the following interface parameters: area, number of residues, number of atoms, number of H-bonds, distribution of residues, distribution of H-bond partners or the distribution of structural interface motifs in either protein-protein or protein-DNA interfaces respectively where complex C is either a protein∶protein, a protein∶DNA or a protein∶protein∶DNA complex. Formula (1) can be easily be expanded to cover quaternary complexes (protein∶protein∶protein∶DNA) as well, but for clarity we have only represented the case for ternary complexes.

It is apparent from formula (1) that interface parameters under discussion, for complexes composed of multiple proteins bound to DNA, can be estimated from protein-protein and single protein-DNA complexes alone. A more precise variant of formula (1), for example in the form of a regression equation, would be possible to derive if we had crystal structures of the same protein in all three states: protein∶protein; protein∶DNA and protein∶protein∶DNA.

Our results indicate that the physical properties of protein∶protein and protein∶DNA complexes, such as interface area, number of interface residues/atoms and hydrogen bonds and the distribution of interface residues and secondary structure motifs are no different in binary or ternary complexes. Thus, if we have two (or more) proteins which bind together, there will be no influence on these interface parameters of their DNA-binding interface when they bind together as a complex to DNA. This claim is not related to the energy of these interactions and it is expected that the interaction rate constants will not be the same for binary and multiple proteins complexes. If two DNA binding proteins can also bind to each other then this will tether them in the vicinity of the DNA such that when one of the proteins binds to DNA the second will have a faster on-rate because it will have a shorter distance to diffuse to find its binding site thus maintain a higher effective local concentration around the DNA. A detailed analysis of rate constants cannot unfortunately be made from crystal structures which are by definition static snapshots of this dynamic process.

### Water molecules in protein-protein and protein-DNA interactions

It has been discussed that water content and water mediated contacts in the protein-DNA interface are important components of protein-DNA interactions [34], [35]. Protein-protein and protein-DNA interfaces contain significant quantities of water [36]. Structural and biochemical data indicate that water-mediated interactions are important for the stability and specificity of recognition, despite the fact that interface solvent molecules exchange rapidly with the bulk solvent [36]. We wanted to evaluate the differences between water mediated contacts at protein-DNA interfaces in protein∶DNA complexes (single proteins bound to DNA) and in protein∶protein∶DNA complexes (multiple proteins bound to DNA). The average number of water mediated contacts between the protein-DNA interfaces of protein∶protein∶DNA complexes is ∼11.82±1.3 (Table S8). This is markedly different from the value of 28 reported for protein∶DNA complexes previously [36]. Similarly, we compared the water mediated contacts in the protein-protein interfaces of protein∶protein and protein∶protein∶DNA complexes. The average number of water molecules for protein-protein interfaces of complexes in the group-MultiProteins∶DNA was ∼4.9±0.83 (Table S8), as compared to ∼22 for protein-protein interactions in binary protein∶protein complexes reported by [36].

These results suggest that water mediated contacts in interfaces of components in protein∶protein∶DNA complexes play less important role in the stability and specificity of recognition then in interfaces of components in the binary protein∶protein and protein∶DNA complexes. However, as we discussed later in the text there are other factors which are more important for stability and specificity of component recognition in protein∶protein∶DNA complexes.

### DNA distortion

**Figure 2:**
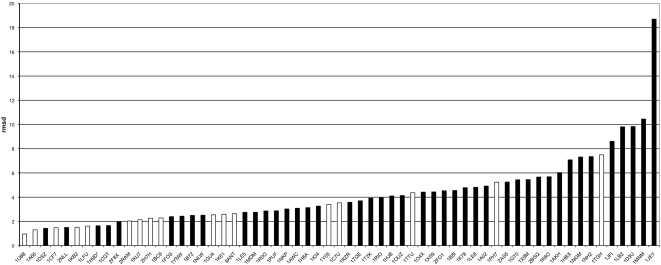
Distribution of rmsd values for measuring DNA distortion. Distribution of rmsd values calculated from fitting each DNA structure in the complexes from group-MultiProteins∶DNA (black bars) and group-SingleSameProtein∶DNA (white bars) to a corresponding canonical B-DNA.

In order to check if DNA structural deformation is higher when multiple proteins bind to DNA we performed computational structural analysis of DNA structures. DNA distortion was measured by calculating the root-mean-square deviation (rmsd) when each DNA structure was fitted onto its corresponding canonical A-DNA or B-DNA structure. Distributions of rmsd values for all complexes from the groups MultiProteins∶DNA (black bars) and SingleSameProtein∶DNA (white bars) were calculated (Figure 2). Statistical analysis of these results showed a significant difference in means of rmsd values (student's t-test with equal or unequal variance as appropriate, p-value<0.02) calculated for all complexes from the groups –MultiProteins∶DNA, -SingleProtein∶DNA and –SingleSameProtein∶DNA calculated after fitting each DNA structure onto the corresponding canonical A-DNA and B-DNA structures (Table 2). Further information for each complex is given inTable S9, S10, S11 and S12. The rmsd values for the group-SubMultiProteins∶DNA are the same as those for the group-MultiProteins∶DNA.

**Table 2:** Measuring DNA distortion.

Dataset of complexes	Average rmsd (±SE) from A-DNA	Average rmsd (±SE) from B-DNA
Group-MultiProteins∶DNA	8.26±0.4	4.71±0.5
Group-SingleProtein∶DNA	5.94±0.2(p<0.001)	3.44±0.2 (p = 0.007)#
Group-SingleSameProtein∶DNA	6.66±0.6 (p = 0.02)	2.87±0.4 (p = 0.004)#

Average rmsd values calculated from fitting each DNA structure in the complexes from group –MultiProteins∶DNA, -SingleProtein∶DNA, and –SingleSameProtein∶DNA to a corresponding canonical A-DNA and B-DNA.

p-values are calculated in comparison with Group A and obtained using the one-tailed Student's t-test.

# unequal variance.

The rmsd values of the group SubSetMultiProteins∶DNA, including comparisons with the group SingleSameProtein∶DNA, are given in Table S13. DNA distortion, however, is significantly higher when multiple proteins are bound to the DNA (Figure 2, Table 2, Table S13). It has been reported that when a single protein binds to DNA it results in a higher rmsd (conformational change) than that seen in the unbound DNA structure [15]. Here we reported that there are also further conformational changes to the structure of DNA which are induced when multiple proteins bind to it.

### Energetic properties of interfaces

The energetic properties of cooperatives are useful for understanding of how the essential macromolecular machines of cellular function are assembled and how they work [37]. We analyzed energetic and thermodynamic properties of different mulitcomponent complexes (protein∶protein∶DNA, protein∶DNA, protein∶protein). In Table 3 we report the free energy of dissociation (Δ*G^diss^*) and the free energy of solvation (Δ*G* ^int^) in kJ/mol for complexes from the four groups –MultiProteins∶DNA, -SubMultiProteins∶DNA, -SingleProtein∶DNA, and –SingleSameProtein∶DNA. In Table 4 we also report energy Z-score values for direct and indirect readouts for the three groups –MultiProteins∶DNA, -SubMultiProteins∶DNA and –SingleProtein∶DNA. The p-values in Table 3 were obtained by comparing the means of Δ*G* ^int^, Δ*G^diss^* and the Z-scores for the direct and indirect readouts using the student's t-test (with equal or unequal variance as appropriate). We could not calculate energy Z-scores for the indirect readouts of the group SubMultiProteins∶DNA because the DNA structure is the same for each complex, so the calculated Z-scores would also be the same. Detailed lists of the Δ*G* ^int^, Δ*G^diss^* and Z-scores for both the direct and indirect readouts of each complex and each group are available in Table S14, S15, S16, S17, S18, S19, S20, S21, S22 and S23.

**Table 3:** Complex energies.

Dataset of complexes	Average (±SE) solvation energy Δ*G* ^int^ (kJ/mol)	Average (±SE) Δ*G^diss^* (kJ/mol)	Average (±SE) energy Z-score for direct readout	Average (±SE)energy Z-score for indirect readout
Group-MultiProteins∶DNA	−234.61.03±18.4	50.41±6.0	−2.81±0.2	−2.36±0.1
Group-SubMultiProteins∶DNA	−123.21±9.8 (p<0.001)#	47.19±4.9 (p = 0.34)	−1.71±0.2 (p<0.001)	—
Group-SingleProtein∶DNA	−114.49±8.6 (p<0.001)#	48.52±5.3 (p = 0.41)	−1.84±0.3 (p = 0.005)#	−2.14±0.1 (p = 0.13)
Group-SingleSameProtein∶DNA	−99.79±15.0 (p<0.001)#	31.06±6.5 (p = 0.03)	−1.34±0.3 (p<0.001)#	−1.48±0.3 (p = 0.007)

Average solvation energy (kJ/mol), free energy barrier of assembly dissociation (kJ/mol), and energy Z-scores for direct and indirect readouts for groups –MultiProteins∶DNA, -SubMultiProteins∶DNA, -SingleProtein∶DNA and –SingleSameProtein∶DNA.

p-values are calculated in comparison with Group-MultiProteins∶DNA and obtained using the one-tailed Student's t-test.

# unequal variance.

**Table 4:** Affinity of components.

Dataset of complexes	Average (±SE) protein-DNA energy binding affinity (kJ/mol)	Average (±SE) protein-DNA overlapping volume (Å^3^)	Average (±SE) number of atoms in collision in protein-DNA interfaces
Group-MultiProteins∶DNA	−39.05±0.9	4.26±0.8	32.06±4.1
Group-SubMultiProteins∶DNA	−30.93±0.5 (p<0.001)#	2.04±0.3 (p = 0.007)#	15.44±1.9 (p<0.001)#
Group-SingleProtein∶DNA	−33.20±0.6 (p<0.001)	3.17±0.56 (p = 0.13)	20.45±1.8 (p = 0.006)#
Group-SingleSameProtein∶DNA	−32.79±0.9(p<0.001)#	2.313±0.8 (p = 0.04)#	15.5±3.3 (p = 0.001)#

Average protein-DNA energy binding affinity (kJ/mol), interface overlapping volume (Å^3^) and average number of interface collision atoms for groups –MultiProteins∶DNA, -SubMultiProteins∶DNA, -SingleProtein∶DNA and –SingleSameProtein∶DNA.

p-values are calculated in comparison with Group-MultiProteins∶DNA and obtained using the one-tailed Student's t-test.

# unequal variance.

Table 4 shows the average protein-DNA energy binding affinity in kJ/mol for the MultiProteins∶DNA, SubMultiProteins∶DNA, SingleProtein∶DNA and SingleSameProtein∶DNA groups; the average protein-DNA overlapping volume (in Å^3^) and the number of atoms in collision at the protein-DNA interfaces. All values were compared against the MultiProteins∶DNA group and a student's t-test was used to calculate the p-values. Further information on these parameters can be found in Table S24, S25, S26, S27 and S28.

The average protein-protein binding energy for complexes from the MultiProteins∶DNA group (which are bound to DNA) is significantly smaller (student's t-test, p-value = 0.05) than that of complexes from group-Protein∶Protein (Table 5). The average solvation energy (Δ*G* ^int^) and free energy barrier of assembly dissociation (Δ*G^diss^*) for protein-protein complexes from group–MultiProteins∶DNA is, respectively, smaller and larger (student's t-test, p-value<0.001) than that found for complexes from group-Protein∶Protein (Table 5). A list of protein-protein binding affinities for every complex in the MultiProteins∶DNA and Protein∶Protein groups may be found in Table S29–S30.

**Table 5:** Protein-protein interfaces energies.

Dataset of complexes	Average (±SE) protein-protein binding free energy (kJ/mol)	Average (±SE) solvation energy Δ*G* ^int^ (kJ/mol)	Average (±SE) Δ*G^diss^* (kJ/mol)
Group-MultiProteins∶DNA	−56.27±6.3	−234.61.03±18.4*	50.41±6.0*
Group-Protein∶Protein	−67.20±2.3 (p = 0.05)#	−81.937±10.1 (p<0.001)#	8.22±2.9 (p<0.001)#

Average protein-protein binding free energy (kJ/mol), average solvation energy (kJ/mol) and average free energy barrier of assembly dissociation (kJ/mol) for protein-protein complexes from group –MultiProteins∶DNA and –Protein∶Protein.

p-values are calculated in comparison with Group-MultiProteins∶DNA and obtained using the one-tailed Student's t-test.

# unequal variance.

* calculated for the whole complex (the same values as in Table 3).

The energetic properties of protein-DNA interfaces of the complexes in group-SubSetMultiProteins∶DNA, including their comparisons with corresponding values from group-SingleSameProtein∶DNA, are given in Tables S31 and S32.

The free energy barrier of assembly dissociation (Δ*G^diss^*, Table 3) is higher for complexes involving multiple proteins bound to DNA (MultiProteins∶DNA) than those involving only single protein-DNA complexes (SubMultiProteins∶DNA, SingleProtein∶DNA and SingleSameProtein). The SingleSameProtein∶DNA and the SubMultiProteins∶DNA groups both contain proteins which are also components of the complexes found in the MultiProteins∶DNA group, but the SubMultiProteins∶DNA group was formed by manually removing the extra protein units from the complexes of group-MultiProteins∶DNA in order to get single protein-DNA complexes. We see that in comparison with the SingleSameProtein∶DNA group, complexes in the MultiProteins∶DNA group have significantly (p = 0.03, student's t-test) higher free energy barriers of assembly dissociation (Δ*G^diss^*). This means that multiple proteins-DNA complexes are more thermodynamically stable than single protein-DNA complexes. Comparing the MultiProteins∶DNA group to the three other groups (SubMultiProteins∶DNA, SingleProtein∶DNA, and SingleSameProtein∶DNA), we find a significantly smaller free energy (student's test, p-value<0.001, Table 3) of solvation gain upon complex formation (Δ*G* ^int^). The same result was found when comparing the MutliProteins∶DNA group to the SubSetMultiProteins∶DNA group (Table S31).

The energy Z-scores for direct and indirect readouts (conformational energy) have more negative values for complexes with multiple proteins bound to DNA (Table 3 and Table S31). More negative Z-scores mean that the target DNA sequence fits into a given protein structure better [29]. Therefore, DNA-binding proteins fit their targets better when they form a ternary complex with DNA. The Z-score also indicates that ternary complexes may be more stable than binary ones. The binding energy affinity, overlapping volume and number of atoms in collision (Table 4) is significantly higher in protein-protein-DNA complexes than in protein-DNA complexes. Differences in overlapping volume and number of atoms in collision are due not only to the bigger interface area (twice protein∶DNA), but also to the higher affinity of multiple proteins binding (interface area sizes for the SingleProteins∶DNA, SingleSameProteins∶DNA and –SubMultiProteins∶DNA groups are similar, butthe SingleProtein∶DNA and SingleSameProtein∶DNA groups have higher protein-DNA binding affinities, overlapping volumes and numbers of atoms in collision than those in the SubMultiProteins∶DNA group, Table 4 and Table S32). Cis-modules that contain transcription factor binding sites (cis-motifs) of transcription factors which make direct physical contact with each other have higher DNA-binding affinities than cis-modules that contain transcription factor binding sites (cis-motifs) of factors without direct mutual contacts. This information may be used for the prediction of cis-regulatory motifs/modules in the following way: if we say that the value of a scoring function for binding sites which are close to one another (where there might be the physical contact between corresponding transcription factors) may have a lower threshold value than a threshold which should be used for scoring function for binding sites that are further away (where there might not be the physical contact between corresponding transcription factors). Modelling DNA∶protein∶protein∶DNA interactions caused by the bending of DNA would also be a possible explanation for introducing a similar strategy; however, there is still not enough information for computational modelling of DNA-bending (i.e. there are not yet any computational strategies which can predict when two transcription factors which are bound to DNA with a long distance between them would have direct physical contact as a consequence of DNA bending). In addition to that, another important implication for the prediction of CRM or cis-motifs is the overlap between transcription factors which have binding sites close to each other. Based on our collision detection results, we realized that sometimes when transcription factors bind to the different grooves of DNA (major and minor) their binding sites can overlap a lot, but from a 3D point of view there is no physical overlap between factors. On the other hand, if two transcription factors bind to the same groove (usually major) then there can be a large overlap between them from a 3D point of view if there is a large overlap between their binding sites (i.e. this situation is not possible). In other words, if care is taken about the structural classification of transcription factors (i.e. if they bind to the major or minor groove) this information can also be used for CRM or cis-motif predictions.

It is interesting to note that protein-protein affinities are higher when proteins are not bound to DNA (Table 5). Interfaces between proteins that are part of a multi-complex (with DNA) can be weaker than those found in binary ones. Binding to DNA may decrease protein-protein affinities, while increasing the overall stability of the complex (significantly higher stability, student's test, p<0.001, Table 5). When two proteins bind freely in solution they are largely unhindered in their rotational movement so they can align themselves using the most energetically favourable orientation which gives them the optimal protein-protein binding energy. When DNA is added to the complex, the three components must arrange themselves to form a global energy minima. However the requirement of binding to DNA introduces a restriction on the possible arrangement of the components such that the protein-protein binding may be weakened by this extra strain but the additional synergistic stability of the three way complex more than compensates for this effect (Table 5).

### Conclusion

It is very difficult to determine the rules governing the assembly of complexes by data-mining alone [38]. Universal conclusions for the types of complexes used are unreliable because of the limited number of available structures (44). However, many general descriptive features can be elucidated even with a modest data collection. As further structures become available, the confidence in the results presented here can be further constrained. The precedent for such studies, using similar or even smaller number of structures is well documented (e.g. [10], [15], [19], [23]).

In this paper, we conclude that protein-protein and protein-DNA interface parameters, such as interface area, number of interface residues/atoms and hydrogen bonds, and distribution of interface residues, hydrogen bonds, van der Walls contacts and secondary structure motifs in complexes where multiple proteins are bound to DNA are no different in protein-protein, single protein-DNA or multiple proteins-DNA complexes. Thus, if we have two (or more) proteins which bind together, there will be no influence on these interface parameters. Also, if we have one protein bound to DNA, then that binding will have no influence (in terms of the interface parameters mentioned) on the types of interface interactions that can occur with subsequent protein-protein complex expansion. The water mediated contacts in interfaces of components in protein∶protein∶DNA complexes play less important role (found in less quantity) in the stability and specificity of recognition then in interfaces of components in the binary protein∶protein and protein∶DNA complexes. Distortion is significantly higher when multiple proteins bind to DNA. This distortion is required to accommodate multiple protein binding events. The combinatorial assembly of transcription factors has been known for a long time to play an important role in stabilizing regulatory complexes. A deeper understanding of structural considerations may be helpful when predicting the assembly of transcription factor complexes. The formation of multiple protein interactions with DNA results in a decrease in protein-protein affinity and an increase in protein-DNA affinity with a net gain in overall stability for a protein-protein-DNA complex. Such effects are clearly important for modelling transcription factor cooperativity.

## Materials and Methods

### Definition of data sets

We selected 75 crystal complexes from the PDB database which contained two or more proteins bound to DNA with a resolution of 3.25 Å or less. We discarded all homologous complexes with less than 30% protein sequence for all protein components using the PISCES server [39], [40]. Our final dataset contained 46 complexes (Table S33). We determined the UniProt ID of each protein component using the tool [41]. This dataset was called group-MultiProteins∶DNA. Most of the complexes from group-MultiProteins∶DNA are ternary (two proteins bound to DNA), but a few of them are quaternary (three proteins bound to DNA). A very few of them contain one protein which does not make contact with DNA but is bound to another protein which does have a direct contact with DNA. We created a second dataset (group-SubMultiProteins∶DNA) from group-MultiProteins∶DNA which consisted of 91 structures (this number is smaller than 92, because some of the proteins do not have direct contact with DNA), each of which was a sub-structure containing only one protein unit plus DNA. In addition, we analysed a set (group-SingleProtien∶DNA, Table S34) of single protein-DNA complexes (102 structures), which was a subset derived from a previous study [16]. We found 17 PDB structures (group-SingleSameProtein∶DNA, Table S35) which contained single proteins and DNA, but the proteins were all components of complexes in group-MultiProteins∶DNA. Corresponding subgroup of group-MultiProteins∶DNA which contains complexes for each where there is a partner in the SingleSameProtein∶DNA group we call this group-SubSetMultiProteins∶DNA (Table S36). The group-Protein∶Protein (Table S37), which contained 70 protein-protein complexes, came from a previous study [9].

### Physical and chemical analysis of interfaces

We used the PISA service from the European Bioinformatics Institute [25], [26] to calculate interface areas and compositions. There are two possibilities for defining the interface between two macromolecular components: the first approach defines the interface as the protein surface area which becomes inaccessible to solvents when two chains come into contact; the second method defines the interface as the set of atoms, where the atom centers from different proteins lie within a distance of 1–5 Å. Both approaches are widely used in macromolecular complex analysis and produce roughly equivalent results. The PISA service uses the first approach. The interface area between macromolecular components M1 and M2 is calculated as the difference in total accessible surface areas of isolated and interfacing structures divided by two, i.e.:
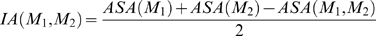
(2)where ASA(M1) and ASA(M2) are the accessible surface areas of macromolecular components M1 and M2 respectively, and ASA(M1M2) is the accessible surface area of the complex of M1 and M2.

We also used the PISA service to calculate hydrogen bonds, salt bridges, disulphide bonds and interface residues. However, PISA provides no information about van der Waals contacts between atoms (residues) because they may be in contact with several other residues. This is the principal difference between the outputs for van der Waals and hydrogen bonds, where inter-atomic links are well determined. However, in order to produce results comparable with previous studies, we have calculated van der Waals contacts in the following way: all atoms not involved in hydrogen bonds but separated by 3.9 Å or less are considered to be interacting through van der Waals contacts [18]. We also analyzed the statistical distribution of amino acid-amino acid and amino acid-nucleotide pairs (“interaction matrices”) for hydrogen bonds and van der Waal contacts. For all amino acid-amino acid and amino acid-nucleotide pairs we calculated contingency tables. The expected values for these tables are based on an assumption of random interactions. We evaluated the contingency tables using Fisher's exact test for count data with simulated p-values based on 200000 repetitions (GNU R). The p-value obtained by Fisher's exact test indicates whether rows and columns in contingency tables are independent or not. However, this does not provide information about which of the pairings are different from expected. To calculate this we performed individual Fisher's tests (GNU R) for each pair.

In order to determine the chemical characteristics of the interfaces, we classified the interface residues using Eisenberg's hydrophobicity scale [42] in a similar way to Lejeune et al. [16]: amino acids are assigned to groups which contain those that are positively charged (Arg and Lys), negatively charged (Asp and Glu), polar (Asn, Gln, His, Ser, and Thr), aliphatic (Ala, Ile, Leu, Met and Val), aromatic (Phe, Trp, and Tyr), and particular (Cys, Gly, and Pro). Multinomial distributions obtained in this study were compared using the Chi-square multinomial goodness-of-fit test.

In addition, a general indication of the hydrophobicity of the interfaces can be estimated using the residue interface propensities. The residue interface propensities give a measure of the relative importance of different amino acid (nucleic acid) residues in all the interfaces of complexes. The propensity values can be calculated using the accessible surface area of residues, as was done by Ellis et al. [10], or using the frequencies of residues, as was done by Lejeune et al. [16]. Both approaches have the same goal, to determine the relative importance of the different residues. Because of its simplicity, we have used the approach described in [16]. Following that, the propensity P_x_ for the interface residues x (x and y are amino acid or DNA structures) can be calculated by:
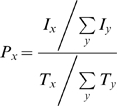
(3)where I_x_ is the total number of residues x in the interface area, T_x_ is the total number of residues in the whole dataset and similar for T_y_ and I_y_. If P_x_>1 it indicates that the residue x is “favoured” and occurs more frequently at interfaces than in the dataset as a whole. If P_x_<1 then residue x is “disfavoured” at interaction sites; in all other cases we can say that residue x is neither over- nor under-represented in the interface region in the complexes. In order to evaluate whether a particular propensity value was significantly different from 1 (either above or below), a statistical bootstrapping method was implemented similar to [10].

### Structural analysis of interfaces

We analyzed the types of secondary structures present within protein-protein and protein-DNA interfaces using the PROMOTIF program [27]. PROMOTIF defines 11 different secondary structure motifs: β-turns, γ-turns, β-bulges, α-helices, 3_10_-helices, β-strands, β-sheets, βαβ units, ψ-loop, β-hairpins, and disulphide bridges. For each structural motif we calculated propensities in the same way as we did for residue propensities (formula (3)).

### Analysis of DNA distortion

DNA distortions were estimated by calculating the root-mean-square deviation (rmsd) when each DNA structure from a complex was fitted onto the corresponding canonical A-DNA and B-DNA structures as in [15], using the whole DNA from crystal strucutres and without normalization to the length of the DNA used. (Regions which are not in interactions do not have significant deformation therefore their contributions to RMSD is not big.) Canonical A-DNA and B-DNA for the nucleotide sequence (with the same length) from the complex were constructed using X3DNA [28]. The fitting was performed with the McLachlan algorithm [43] as implemented in the program ProFit [44].

### Analysis of water molecules in protein-protein and protein-DNA interactions

Water molecules are defined as interface water molecules if they are less than 3.5 Å from the atoms of the two components of a complex, as in [21]. This analysis was restricted to those structures with 2.4 Å or better resolution as the identification of water in the electron density map may be ambiguous at lower resolutions [21].

### Analysis of energetic properties of interfaces

The chemical stability of complexes was analysed by calculating the free energy barrier of assembly dissociation (Δ*G^diss^*) and the solvation free energy gain upon formation of the assembly (Δ*G* ^int^) in kJ/mol using PISA. Assemblies with higher positive values of Δ*G^diss^* are more thermodynamically stable, and that value indicates that an external driving force is required to dissociate the assembly. For the calculation of Δ*G* ^int^ and Δ*G^diss^* we used structures from all six groups (-MultiProteins∶DNA, -SubMultiProteins∶DNA, -SingleProtein∶DNA, -SingleSameProtein∶DNA, -SubSetMultiProteins∶DNA and –Protein∶Protein).

We calculated Z-scores for intermolecular and intramolecular readouts using a ReadOut server [29]. Direct readouts (direct contacts between amino acids and base pairs) and water-mediated contacts are intramolecular energies, whereas indirect energies quantify sequence-dependent DNA conformational energies. The specificity of the complex is given by the Z-score, and larger negative values correspond to higher specificities [45]. For the calculation of the Z-score, we used the data from groups –MultiProteins∶DNA, -SubMultiProteins∶DNA, -SingleProteins∶DNA, -SingleSameProtein, -SubSetMultiProteins∶DNA.

We calculated binding energy affinities (protein-DNA) for each structure in groups –MultiProteins∶DNA, -SubMultiProteins∶DNA, -SingleProtein∶DNA, -SingleSameProtein∶DNA, and –SubSetMultiProteins∶DNA using the DFIRE energy function [30].

We compared the mean of Δ*G* ^int^, Δ*G^diss^*, the Z-score for direct and indirect readouts, and the binding energy affinities between group-MultiProteins∶DNA and each of the other three groups (-SubMultiProteins∶DNA, -SingleProtein∶DNA and –SingleSameProtein∶DNA) using student's t-test (one-tailed). Differences in the variances of corresponding values between groups were calculated using Bartlett's test. In those cases where we had significant differences in variance between groups, we used student's t-test with unequal variance.

For protein-protein complexes (group-Protein∶Protein) we calculated Δ*G* ^int^ and Δ*G^diss^* using the PISA server. We have calculated protein-protein binding energy affinities for complexes from group-Protein∶Protein and protein-protein subcomplexes from group-MultiProteins∶DNA using DCOMPLEX [31]. We also compared the average protein-protein binding affinities, average values of Δ*G* ^int^ and Δ*G^diss^* between groups –MultiProteins∶DNA and –Protein∶Protein.

### Collision detections and overlapping volume of two macromolecules

We calculated the number of atoms in collision and the volume of the overlapping region for protein-protein and protein-DNA interfaces from groups –MutliProteins∶DNA, -SubMultiProteins∶DNA, -SingleProtein∶DNA and –SingleSameProtein∶DNA. Collision detection between two macromolecules is actually collision detection between complex objects, where these objects are composed of collections of spheres. The most straightforward algorithm for modelling this problem (in the case of two objects: A1 and A2) is checking each sphere from object A1 against each sphere from object A2, and we know that objects A1 and A2 intersect only if one or more of these pairs intersect. For two objects with M and N spheres this algorithm requires O(MN) time to complete. There are several geometric algorithms with better speed for collision detection between objects in 3D space such as those based on bounding-volume (BV) hierarchies [46], [47], algorithms based on axis-aligned bounding boxes AABB [48], [49], algorithms based on oriented bounding boxes [50], and spatial hashing [51], [52]. In this study we used an algorithm for collision detection based on spatial hashing [51] and axis-aligned bounding boxes AABB [48], [49]. To perform this, we executed the following steps (Figure S7):

- Make an AABB around each macromolecule.
- Check if any pair of AABBs overlaps. In order for two AABBs to overlap they must overlap on all three special axes. If there is no overlap then they cannot be in collision. Otherwise they may be in collision.
- Perform a special hashing on the overlapping region of each pair of AABBs that contain macromolecules that may be in collision.

**Figure 3:**
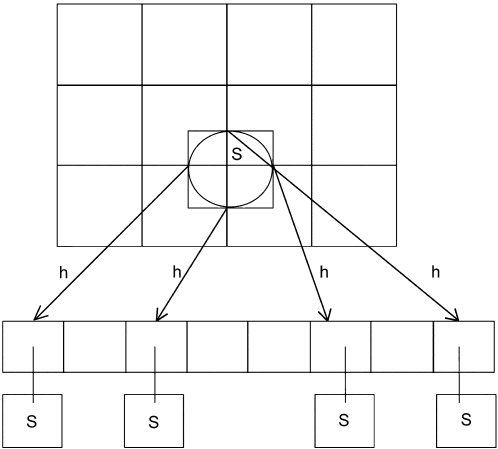
Assignment of hash values to the atoms of a macromolecule. Hash values are computed for all the grid cells covered by the AABB of the sphere (atom) from a macromolecule. In this case, sphere S falls into four cells and they are mapped onto a hash table.

The overlapping region (a rectangular prism) is divided into a three dimensional grid of cells. Each cell in the grid is a cube with side lengths equal to the diameter of the largest sphere (atom) in the macromolecule. This is a uniform spatial subdivision. Each sphere (atom) in the macromolecule can be assigned to the cell in which it lies using a hash function as follows: First it is necessary to make an AABB for each sphere. Then the (x,y,z) coordinates of the six side centers are assigned to their corresponding cells using the hash function (Figure 3).

The hash function we used is given in formula (4) [52]:
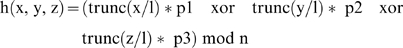
(4)where p1, p2, and p3 are large prime numbers (in our case 73856093, 19349663 and 83492791 respectively). The size of a cell is defined as 1, the hash table has a size “n”. The function “trunc(x)” rounds the real number “x” down to the next integer. The function “xor” is a Boolean exclusive-or operation.

To test whether a sphere “S” from another macromolecule intersects with the first macromolecule, it suffices to find out if that sphere intersects any of the spheres of another macromolecule that share a cell with “S”. The time complexity of this algorithm is linear “O(n)”, where “n” is the number of sphere-atoms found in the overlapping region between two macromolecules AABBs.

We extended the collision detection algorithm so that it is able to calculate the number of atoms which are in collision and their overlapping volume. Instead of stopping the analysis as soon as two atoms are found to be in collision, the algorithm is continued until all of the atoms from the different macromolecules have been counted. From this it is a simple matter to estimate the overlapping volume from the colliding spheres.

Web-base implementation of the algorithm is freely available from http://promoterplot.fmi.ch/Collision1/. The user submits pdb files and then specifies which chains to test for collision. The output lists the number of atoms from each protein which are in collision and the volume of overlapping region. In addition, with this tool user may display 3D complex from PDB files as interactive web pages using the Corotna VRML Client plug-in or any other VRML plug-in.

## Supporting Information

Figure S1 Distribution of H-bonds according to the nucleotide part (group-MultiProteins∶DNA).
(0.91 MB TIF)

Figure S2 Distribution of amino acids involved in H-bonds in protein-protein and protein-DNA interfaces (group-MultiProteins∶DNA).
(0.93 MB TIF)

Figure S3 Distribution of H-bonds according to the nucleotide part (group-SingleSameProtein∶DNA).
(0.91 MB TIF)

Figure S4 Distribution of H-bonds according to the nucleotide part (group-SubSetMultiProteins∶DNA).
(0.91 MB TIF)

Figure S5 Amino acid propensities for protein-protein and DNA-protein interfaces (group MultiProteins∶DNA). Propensity values which are significantly different from 1 (either above or below), as evaluated using the statistical bootstrapping method, are marked with “*”.
(1.08 MB TIF)

Figure S6 Distribution of amino acids involved in interaction sites of protein-protein and DNA-protein (group-MultiProteins∶DNA).
(1.07 MB TIF)

Figure S7 Visualization of first several steps of the collision detection algorithm. Situation (A) represents scenario when there is on overlapping between two macromolecules and corresponding axis-aligned bounding boxes either; situation (B) represents scenario when there is no overlapping between two macromolecules but with overlapping between corresponding axis-aligned bounding boxes; situation (C) represents scenario when there is overlapping between two macromolecules and corresponding axis-aligned bounding boxes.
(3.00 MB TIF)

Table S1 Detailed list of interface parameters for each complex from group-MultiProteins∶DNA
(0.09 MB PDF)

Table S2 The number of observed hydrogen bonds between amino acid and nucleotide moieties in protein-DNA interfaces (group-MultiProteins∶DNA)
(0.07 MB DOC)

Table S3 The number of observed hydrogen bonds between amino acid and nucleotide moieties in protein-DNA interfaces (group-SingleSameProtein∶DNA)
(0.07 MB DOC)

Table S4 The number of observed hydrogen bonds between amino acid and nucleotide moieties in protein-DNA interfaces (group-SubSetMultiProteins∶DNA).
(0.06 MB DOC)

Table S5 Number of observed van der Waals contacts between amino acid and nucleotide moieties in protein-DNA interfaces (group-MultiProteins∶DNA).
(0.06 MB DOC)

Table S6 Number of observed van der Waals contacts between amino acid and nucleotide moieties in protein-DNA interfaces (group-SingleSameProtein∶DNA).
(0.07 MB DOC)

Table S7 Number of observed van der Waals contacts between amino acid and nucleotide moieties in protein-DNA interfaces (group-SubSetMultiProteins∶DNA).
(0.06 MB DOC)

Table S8 The number of water-mediated contacts in protein-protein and protein-DNA intrerfaces of selected complexes in group-MultipleProteins∶DNA
(0.04 MB PDF)

Table S9 Detailed list of rmsd values calculated from fitting each DNA structure in the complexes from group-MultiProteins∶DNA to a corresponding canonical A-DNA and B-DNA.
(0.04 MB PDF)

Table S10 Detailed list of rmsd values calculated from fitting each DNA structure in the complexes from group-SingleProtein∶DNA to a corresponding canonical A-DNA and B-DNA.
(0.04 MB PDF)

Table S11 Detailed list of rmsd values calculated from fitting each DNA structure in the complexes from group-SingleSameProtein∶DNA to a corresponding canonical A-DNA and B-DNA.
(0.03 MB PDF)

Table S12 Detailed list of rmsd values calculated from fitting each DNA structure in the complexes from group-SubSetMutliProteins∶DNA to a corresponding canonical A-DNA and B-DNA.
(0.04 MB PDF)

Table S13 Average rmsd values calculated from fitting each DNA structure in the complexes from group -SubSetMultiProteins∶DNA and -SingleSameProtein∶DNA to a corresponding canonical A-DNA and B-DNA.
(0.03 MB DOC)

Table S14 Detailed list of energies for each complex in group-MultiProteins∶DNA
(0.04 MB PDF)

Table S15 Detailed list of energies for each complex in group-SubMultiProteins∶DNA
(0.04 MB PDF)

Table S16 Detailed list of energies for each complex in group-SingleProtein∶DNA
(0.04 MB PDF)

Table S17 Detailed list of energies for each complex in group-SingleSameProtein∶DNA
(0.04 MB PDF)

Table S18 Detailed list of energies for each complex in group-SubSetMultiProteins∶DNA
(0.04 MB PDF)

Table S19 Detailed list of energies Z-scores (direct and indirect readouts) for each complex in group-MultiProteins∶DNA
(0.04 MB PDF)

Table S20 Detailed list of energies Z-scores (direct and indirect readouts) for each complex in group-SubMultiProteins∶DNA
(0.04 MB PDF)

Table S21 Detailed list of energies Z-scores (direct and indirect readouts) for each complex in group-SingleProtein∶DNA
(0.04 MB PDF)

Table S22 Detailed list of energy Z-scores (direct and indirect readouts) for each complex in group-SingleSameProtein∶DNA
(0.04 MB PDF)

Table S23 Detailed list of energy Z-scores (direct and indirect readouts) for each complex in group-SubSetMultiProteins∶DNA
(0.04 MB PDF)

Table S24 Detailed list of protein-DNA energy binding affinity, overlapping volume and number of atoms in collision for each complex in group-MultiProteins∶DNA
(0.04 MB PDF)

Table S25 Detailed list of protein-DNA energy binding affinity, overlapping volume and number of atoms in collision for each complex in group-SubMultiProteins∶DNA
(0.05 MB PDF)

Table S26 Detailed list of protein-DNA energy binding affinity, overlapping volume and number of atoms in collision for each complex in group-SingleProtein∶DNA
(0.05 MB PDF)

Table S27 Detailed list of protein-DNA energy binding affinity, overlapping volume and number of atoms in collision for each complex in group-SingleSameProtein∶DNA
(0.04 MB PDF)

Table S28 Detailed list of protein-DNA energy binding affinity, overlapping volume and number of atoms in collision for each complex in group-SubSetMultiProteins∶DNA
(0.04 MB PDF)

Table S29 Detailed list of protein-protein binding free energy for each protein-proteincomplex in group-MultiProteins∶DNA
(0.04 MB PDF)

Table S30 Detailed list of protein-protein binding free energy for each protein-proteincomplex in group-Protein∶Protein
(0.06 MB PDF)

Table S31 Average solvation energy (kJ/mol), free energy barrier of assembly dissociation (kJ/mol), and energy Z-scores for direct and indirect readouts for groups -SubSetMultiProteins∶DNA, -SingleSameProtein∶DNA
(0.03 MB DOC)

Table S32 Average protein-DNA energy binding affinity (kJ/mol), interface overlapping volume (Å3) and average number of interface collision atoms for groups -SubSetMultiProteins∶DNA, -SingleSameProtein∶DNA
(0.03 MB DOC)

Table S33 List of PDB IDs used in the study (group-MultiProteins∶DNA), with description of component (including Swiss Prot ID) and biological process of components.
(0.08 MB DOC)

Table S34 The list of PDB codes of complexes from group-SingleProtein∶DNA
(0.03 MB DOC)

Table S35 The list of PDB codes of complexes from group-SingleSameProtein∶DNA
(0.03 MB DOC)

Table S36 The list of PDB codes of complexes from group-SubSetMultiProteins∶DNA
(0.03 MB DOC)

Table S37 The list of PDB codes of complexes from group-Protein∶Protein
(0.03 MB PDF)
