# Supplementary figures and images for: Computational Structural Analysis: Multiple Proteins Bound to DNA
Source: PLoS One. 2008 Sep 19;3(9):e3243. doi: 10.1371/journal.pone.0003243 (PMC2532747; doi:10.1371/journal.pone.0003243)

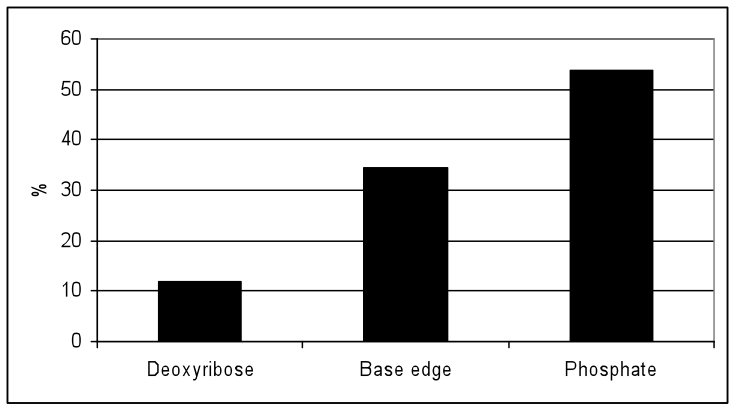

Supplement: Figure S1 — Distribution of H-bonds according to the nucleotide part (group-MultiProteins∶DNA). (0.91 MB TIF) [file pone.0003243.s001.tif]

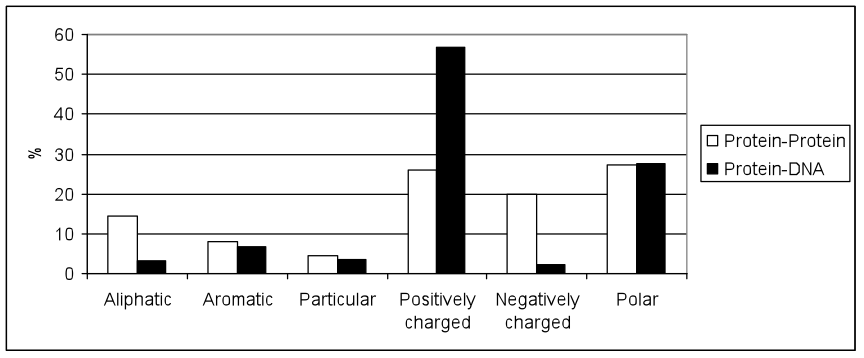

Supplement: Figure S2 — Distribution of amino acids involved in H-bonds in protein-protein and protein-DNA interfaces (group-MultiProteins∶DNA). (0.93 MB TIF) [file pone.0003243.s002.tif]

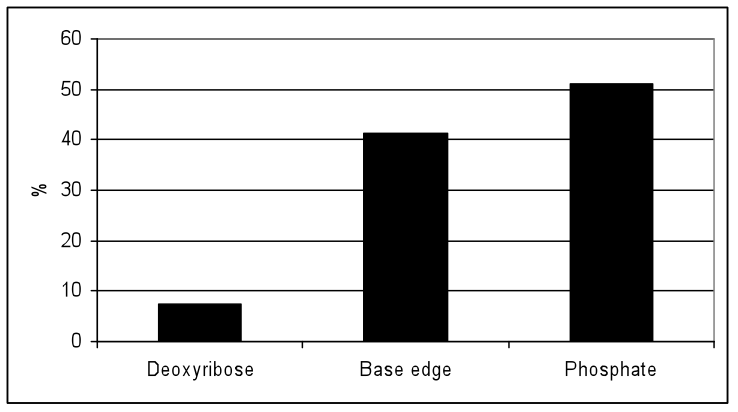

Supplement: Figure S3 — Distribution of H-bonds according to the nucleotide part (group-SingleSameProtein∶DNA). (0.91 MB TIF) [file pone.0003243.s003.tif]

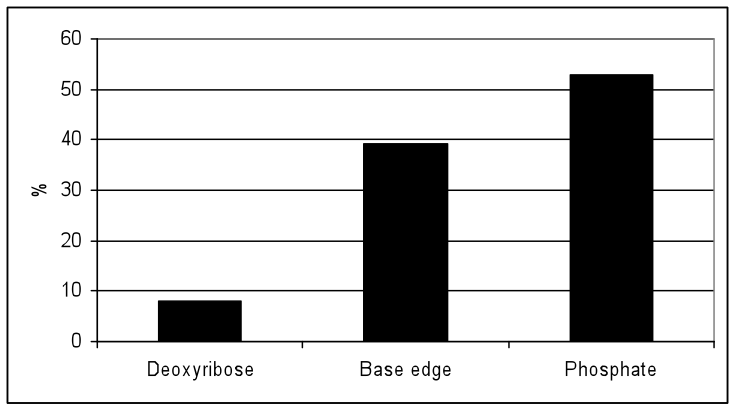

Supplement: Figure S4 — Distribution of H-bonds according to the nucleotide part (group-SubSetMultiProteins∶DNA). (0.91 MB TIF) [file pone.0003243.s004.tif]

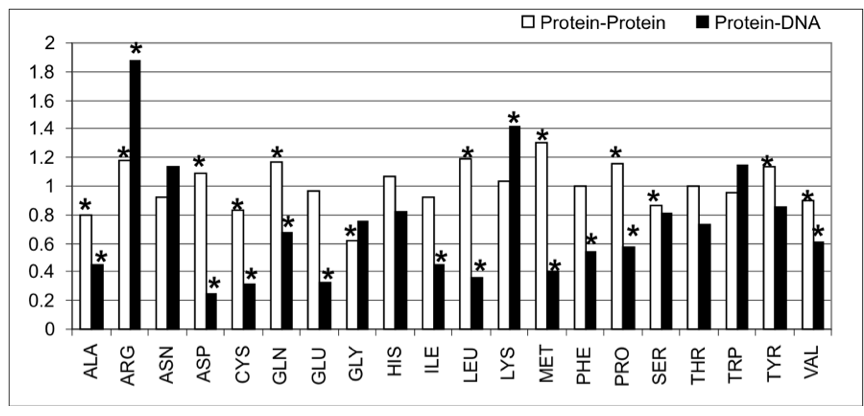

Supplement: Figure S5 — Amino acid propensities for protein-protein and DNA-protein interfaces (group MultiProteins∶DNA). Propensity values which are significantly different from 1 (either above or below), as evaluated using the statistical bootstrapping method, are marked with “*”. (1.08 MB TIF) [file pone.0003243.s005.tif]

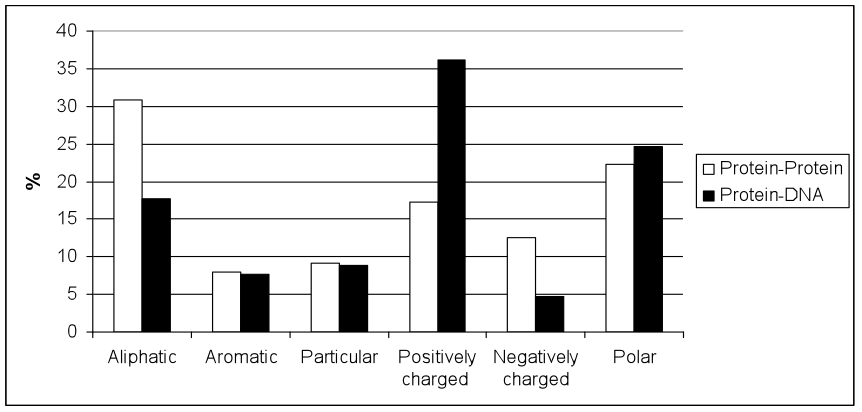

Supplement: Figure S6 — Distribution of amino acids involved in interaction sites of protein-protein and DNA-protein (group-MultiProteins∶DNA). (1.07 MB TIF) [file pone.0003243.s006.tif]

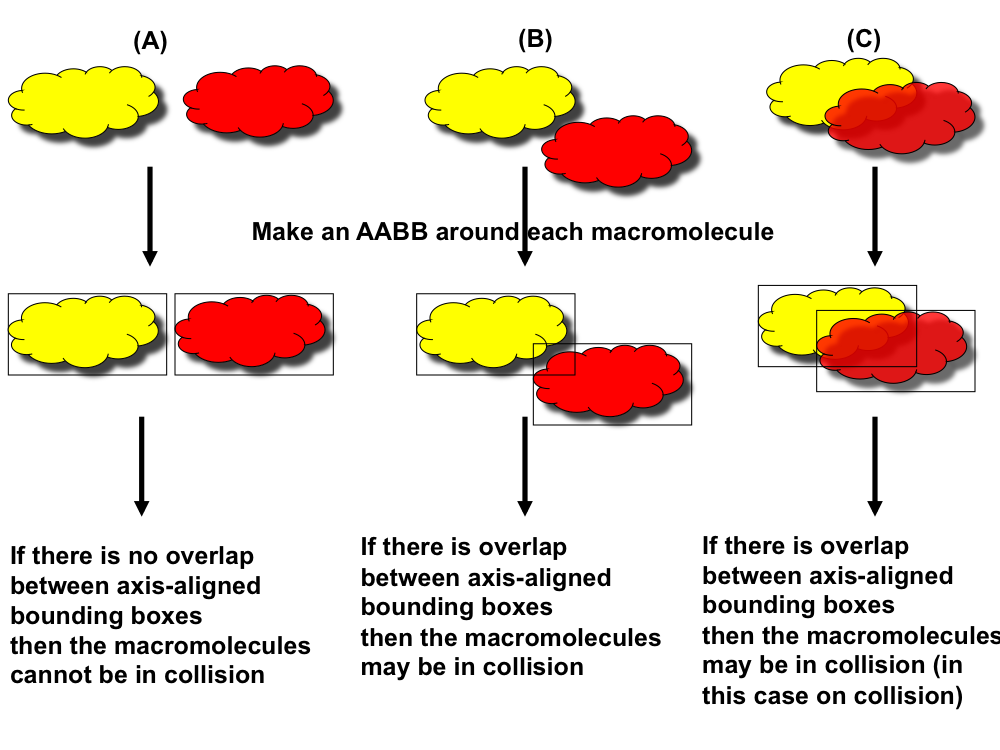

Supplement: Figure S7 — Visualization of first several steps of the collision detection algorithm. Situation (A) represents scenario when there is on overlapping between two macromolecules and corresponding axis-aligned bounding boxes either; situation (B) represents scenario when there is no overlapping between two macromolecules but with overlapping between corresponding axis-aligned bounding boxes; situation (C) represents scenario when there is overlapping between two macromolecules and corresponding axis-aligned bounding boxes. (3.00 MB TIF) [file pone.0003243.s007.tif]
