# Supplementary material for: Computational Structural Analysis: Multiple Proteins Bound to DNA
Source: PLoS One. 2008 Sep 19;3(9):e3243. doi: 10.1371/journal.pone.0003243 (PMC2532747; doi:10.1371/journal.pone.0003243)
Supplement: Table S1 — Detailed list of interface parameters for each complex from group-MultiProteins∶DNA (0.09 MB PDF) [file pone.0003243.s008.pdf]

Table S1. Detailed list of interface parameters for each complex from group-MultiProteins:DNA

	Component 1- # interface // surface // total atoms	Component 2- # interface // surface // total atoms	Component 1- # interface // surface // total residues	Component 2- # interface // surface // total residues	Componenet 1 ASA interface // total	Component 2 ASA interface // total	Component 1 solvation energy (isolated structure // gain at complexation // average gain // p-value)	Component 2 solvation energy (isolated structure // gain at complexation // average gain // p-value)
**1A02**								
**protein-protein**								
fos (F)-jun(J)	94 // 360 // 426	100 // 373 // 443	21 // 52 // 52	22 // 53 // 53	1001.3 // 5776.0	1017.8 // 5685.9	-27.1 // -8.0 // -1.1 // 0.072	-19.5 // -8.2 // -0.5 // 0.043
fos (F)-nfat(N)	46 // 373 // 443	45 // 1329 // 2204	15 // 53 // 53	16 // 264 // 280	388.4 // 5685.9	4106 // 15437.7	-19.5 // 1.2 // -0.2 // 0.686	-231.1 // 0.6 // -0.8 // 0.744
jun (J)-nfat(N)	32 // 360 // 426	35 // 1329 // 2204	7 // 52 // 52	11 // 264 //280	335.5 // 5776.0	311.1 // 15437.7	-27.1 // 3.3 // -0.4 // 0.896	-231.1 // 0.1 // -0.6 // 0.649
**DNA-protein**								
DNA(A)-jun(J)	29 // 338 // 410	25 // 360 // 426	4 // 20 // 20	8 // 52 // 52	280.9 // 4559.6	290.2 // 5776.0	54.0 // -8.6 // -4.6 // 0.161	-27.1 // 1.7 // -0.3 // 0.792
DNA(B)-jun(J)	31 // 329 // 404	30 // 360 // 426	6 // 20 // 20	6 // 52 // 52	262.1 // 4484.7	290.0 // 5776.00	32.8 // -4.1 // - 3.1 // 0.391	-27.1 // 1.8 // -0.4 // 0.790
DNA(A)-fos(F)	43 // 338 // 410	36 // 373 // 443	7 // 20 // 20	8 // 53 // 53	325.6 // 4559.6	370.4 // 5685.9	54.0 // -6.0 // -6.9 //0.557	-19.5 // 1.3 // -0.2 // 0.716
DNA(B)-fos(F)	34 // 329 // 404	30 // 373 // 443	6 // 20 // 20	10 // 53 // 53	311.1 // 4484.7	300.6 // 5685.9	32.8 // -9.1 // -3.4 // 0.099	-19.5 // 0.8 // -0.1 // 0.659
DNA(A)-nfat(N)	44 // 338 // 410	39 // 1329 // 2204	10 // 20 // 20	13 // 264 // 280	364.2 // 4559.6	412.2 // 15437.7	54.0 // -2.8 // -7.0 // 0.801	-231.1 // 4.8 // -0.7 // 0.990
DNA(B)-nfat(N)	59 // 329 // 404	67 // 1329 // 2204	8 // 20 // 20	19 // 264 // 280	626.5 // 4484.7	593.4 // 15437.7	32.8 // -9.7 // -5.9 // 0.245	
**1AKH**								
**protein-protein**								
MATING-TYPE PROTEIN A-1(A) -MATING-TYPE PROTEIN ALPHA-2(B)	67 // 271 // 395	59 // 435 // 606	19 // 48 // 49	15 // 75 // 78	573.9 // 3547.4	651.8 // 6228.3	-36.2 // -2.3 // -2.0 // 0.489	-42.5 // -5.3 // -3.3 // 0.266
**DNA-protein**								
DNA(C,D)-MATING-TYPE PROTEIN A-1(A)	74 // 652 // 855	65 // 271 // 395	13 // 42 // 42	17 // 48 // 49	640.6 // 7816.0	715.5 // 3547.4	86.8 // -13.5 // -9.9 // 0.271	-36.2 // -0.5 // -2.0 // 0.682
DNA(C,D)-MATING-TYPE PROTEIN ALPHA-2(B)	129 // 652 // 855	96 // 435 // 606	18 // 42 // 42	23 // 75 // 78	933.4 // 7816.0	1023.6 // 6228.3	86.8 // -11.2 // -17.2 // 0.764	-42.5 // 5.3 // -5.4 // 0.994
**1AWC**								
**protein-protein**								
GA BINDING PROTEIN ALPHA (A) - GA BINDING PROTEIN BETA 1 (B)	90 // 547 // 907	81 // 653 // 1156	24 // 104 // 110	22 // 139 // 153	795.8 // 6498.3	856.8 // 7275.7	-103.8 // -4.2 // -1.5 // 0.210	-142.2 // -8.1 // -1.6 // 0.013
**DNA-protein**								
DNA(E)-GA BINDING PROTEIN ALPHA (A)	43 // 354 // 421	50 // 547 // 907	6 // 21 // 21	15 // 104 // 110	477.7 // 4713.8	401.1 // 6498.3	50.5 // -8.7 // -6.1 // 0.293	-103.8 // -1.4 // -0.9 // 0.412
DNA(D)-GA BINDING PROTEIN ALPHA (A)	42 // 362 // 431	41 // 547 // 907	7 // 21 // 21	10 // 104 // 110	382.8 // 4792.7	350.2 // 6498.3	24.2 // -7.1 // -2.8 // 0.183	-103.8 // 0.5 // -0.7 // 0.737
DNA(E)- GA BINDING PROTEIN BETA 1 (B)	3 // 362 // 431	3 // 653 // 1156	1 // 21 // 21	2 // 139 // 153	23.1 // 4792.7	20.2 // 7275.7	24.2 // -1.1 // -0.2 // 0.258	-142.2 // -0.3 // -0.1 // 0.281
DNA(D)- GA BINDING PROTEIN BETA 1 (B)	2 // 354 // 421	4 // 653 // 1156	1 // 21 // 21	2 // 139 // 153	14.9 // 4713.8	15.4 // 7275.7	50.5 // -0.5 // -0.3 // 0.327	-142.2 // 0.4 // -0.1 // 0.840
**1B72**								
**protein-protein**								
HOMEOBOX PROTEIN HOX B1(A)-PBX1(B)	40 // 391 // 559	41 // 381 // 576	9 // 66 // 68	14 // 71 // 73	402.0 // 5541.1	363.9 // 5104.1	-40.3 // -4.5 // -2.1 // 0.158	-51.5 // -2.3 // -1.1 // 0.304
**DNA-protein**								
DNA(E)-HOMEOBOX PROTEIN HOX B1(A)	60 // 343 // 407	44 // 391 // 559	11 // 20 // 20	13 // 66 // 68	445.5 // 4580.5	460.5 // 5541.1	34.7 // -6.7 // -6.1 //0.455	-40.3 // 0.8 // -2.4 // 0.893
DNA(D)-HOMEOBOX PROTEIN HOX B1(A)	41 // 341 // 407	42 // 391 // 559	6 // 20 // 20	12 // 66 // 68	364.7 // 4601.6	372.1 // 5541.1	40.6 // -5.2 // -4.9 // 0.46	-40.3 // 0.6 // -2.2 // 0.877
DNA(E)-PBX1(B)	61 // 343 // 407	42 // 381 // 576	11 // 20 // 20	17 // 71 // 73	485.3 // 4580.5	499.4 // 5104.1	34.7 // -6.2 // -6.2 // 0.484	-51 // 2.4 // -1.1 // 0.922
DNA(D)-PBX1(B)	50 // 341 // 407	54 // 381 // 576	5 // 20 // 20	14 // 71 // 73	441.3 // 4601.6	454.8 // 5104.1	40.6 // -5.5 // -6.0 // 0.525	-51.5 // 1.5 // -1.4 // 0.850
**1B8I**								
**protein-protein**								
ULTRABITHORAX HOMEOTIC PROTEIN IV (A) - HOMEOBOX PROTEIN EXTRADENTICLE (B)	34 // 384 // 557	30 // 313 // 471	7 // 60 // 62	10 // 56 // 58	314.7 // 5344.5	287.4 // 4040.9	-45 .4 // -4.6 // 1.3 // 0.071	-40.1 // -1.2 // -0.6 // 0.387
**DNA-protein**								
DNA (C) - ULTRABITHORAX HOMEOTIC PROTEIN IV (A)	60 // 255 // 301	44 // 384 // 557	11 // 15 // 15	13 // 60 // 62	417.9 // 3449.5	453.7 // 5344.5	27.1 // -3.7 // -6.4 // 0.668	-45.4 // 0.9 // -1.7 // 0.833
DNA(D) - ULTRABITHORAX HOMEOTIC PROTEIN IV (A)	52 // 256 // 308	48 // 384 // 557	6 // 15 // 15	13 // 60 // 62	465.3 // 3540.8	445.8 // 5344.5	30.3 // -11.0 // -6.2 // 0.182	-45.4 // 2/0 // -1.8 // 0.911
DNA (C) - HOMEOBOX PROTEIN EXTRADENTICLE (B)	43 // 255 // 301	37 // 313 // 471	9 // 15 // 15	13 // 56 // 58	391.4 // 3449.5	374.3 // 4040.9	27.1 // -8.2 // -4.6 // 0.230	-40.1 // 2.3 // -0.7 // 0.918
DNA (D) - HOMEOBOX PROTEIN EXTRADENTICLE (B)	45 // 256 // 308	46 // 313 // 471	5 // 15 // 15	10 // 56 // 58	390.7 // 3540.8	434.6 // 4040.9	30.3 // -2.0 // -5.3 // 0.743	-40.1 // 3.2 // -0.9 // 0.949
**1CF7**								
**protein-protein**	62 // 350 // 525	60 // 434 // 658	19 // 66 // 67	16 // 78 //82	609.2 // 4683.3	629.7 // 5534.2	-50.7 // -6.1 // -2.1 // 0.068	-61.0 // -7.5 // -1.7 // 0.024
E2F4 (A) - DP2 (B)								
**DNA-protein**								
DNA (C) - E2F4 (A)	42 // 266 // 305	41 // 350 // 525	7 // 15 // 15	10 // 66 // 67	362.5 // 3585.3	384.3 // 4683.3	55.6 // -4.8 // -8.8 // 0.784	-50.7 // 2.2 // -1.4 // 0.927
DNA (D) - E2F4 (A)	55 // 263 // 310	49 // 350 // 525	10 // 15 //15	15 // 66 // 67	466.8 // 3564.8 // 4683.3	14.9 // -5.6 // -3.1 // 0.314	-50.7 // 1.5 // -1.7 // 0.893	
DNA (C) - DP2 (B)	35 // 266 // 305	33 // 434 // 568	5 // 15 // 15	9 // 78 // 82	294.5 // 3585.3	311.0 // 5534.2	55.6 // -3.8 // -7.3 // 0.774	-61 // 1.8 // -0.9 // 0.893
DNA (D) - DP2 (B)	33 // 263 // 310	38 // 434 // 658	6 // 15 // 15	12 // 78 // 82	315.0 // 3564.8	337.9 // 5534.2	14.9 // -6.4 // -1.9 // 0.144	-61/.0 // 2.4 // -1.1 // 0.921
**1CQT**								
**protein-protein**								
TF1 (B) - Association factor (J)	65 // 723 // 1042	45 // 161 // 184	17 // 129 // 129	16 // 23 // 23	484.6 // 9273.6	537.5 // 2667.8	-84 3 // -0.8 // -2.2 // 0.690	-8.5 // -5.2 // -2 // 0.143
**DNA-protein**								
DNA (O) - TF1 (B)	90 // 248 // 293	96 // 723 // 1042	11 // 14 // 14	29 // 129 // 129	852.8 // 3345.2	824.5 // 9273.6	28.1 // -12.7 // -10.2 // 0.344	-84.3 // 2.3 // -3.2 // 0.936
DNA (P) - TF1 (B)	81 // 245 // 281	70 // 723 // 1042	11 // 14 // 14	24 // 129 // 129	712.8 // 3305.5	739.4 // 9273.6	34.9 // -11.8 // -11.5 // 0.468	-84.3 // 3.1 // -2.3 // 0.960
DNA (O) - Association factor (J)	23 // 248 // 293	17 // 161 // 184	5 // 14 // 14	5 // 23 // 23	190.0 // 3345.2	191.0 // 2667.8	28.1 // -1.6 // -2.6 // 0.596	-8.5 // -1.0 // -0.7 // 0.468
DNA (P) - Association factor (J)	15 // 245 // 281	15 // 161 // 184	3 // 14 // 14	4 // 23 // 23	140.6 // 3305.5	143.8 // 2667.8	34.9 // -5.0 // -2.1 // 0.163	-8.5 // -1.9 // -0.7 // 0.233
**1D3U**								
**protein-protein**								
TATA binding protein (A) - TF IIB (B)	72 // 863 // 1415	73 // 949 // 1595	19 // 168 // 181	22 // 174 // 201	742.0 // 10503.1	746.6 // 11080.4	-174.5 // 0.9 // -2.0 // 0.844	-175.8 // -4.6 // -0.7 // 0.104
**DNA-protein**								
DNA (C) - TATA binding protein (A)	82 // 401 // 493	85 // 863 // 1415	9 // 24 // 24	32 // 168 // 181	826.3 // 5446.4	726.3 // 10503.1	48.6 // -0.2 // -9.9 // 0.931	-174.5 // -6.6 // -2.4 // 0.062
DNA (D) - TATA binding protein (A)	90 // 388 // 464	91 // 863 // 1415	10 // 23 // 23	33 // 168 // 181	829.4 // 5271.6	710.4 // 10503.1	62.3 // -0.2 // -14.5 // 0.981	-174.5 // - 4.7 // -2.6 // 0.193
DNA (C) - TF IIB(B)	63 // 401 // 493	64 // 949 // 1595	11 // 24 // 24	21 // 174 // 201	604.2 // 5446.4	542.7 // 11080.4	48.6 // -12.5 // -7.6 // 0.203	-175.8 // 1.4 // -0.6 // 0.750
DNA (D) - TF IIB (B)	58 // 388 // 464	53 // 949 // 1595	11 // 23 // 23	19 // 174 // 201	549.9 // 5271.6	492.0 // 11080.4	62.3 // -9.1 // -9.3 // 0.504	-175.8 // 0.8 // -0.5 // 0.687
**1DSZ**								
**protein-protein**								
RETINOIC ACID RECEPTOR ALPHA (A) - RETINOIC ACID RECEPTOR RXR-ALPHA (B)	25 // 361 // 575	18 // 432 // 667	8 // 68 // 75	6 // 81 // 84	166.7 // 4784.3	191.0 // 6035.8	-54.5 // -0.0 // -0.6 // 0.637	-69.6 // -0.5 // 0.2 // 0.366
**DNA-protein**								
DNA(C) - RETINOIC ACID RECEPTOR ALPHA (A)	37 // 254 // 309	41 // 361 // 575	5 // 15 // 15	12 // 68 // 75	375.5 // 3496.0	337.5 // 4784.3	20.5 // -5.2 // -3.0 // 0.301	-54.5 // 1.6 // -0.9 // 0.874
DNA (D) - RETINOIC ACID RECEPTOR ALPHA (A)	42 // 260 // 300	42 // 361 // 575	7 // 15 // 15	17 // 68 // 75	394.3 // 3514.4	387.1 // 4784.3	33.7 // -6.1 // -5.4 // 0.433	-54.5 // 3.2 // -1.0 // 0.960
DNA (C) - RETINOIC ACID RECEPTOR RXR-ALPHA (B)	46 // 254 // 309	54 // 432 // 667	6 // 15 // 15	17 // 81 // 84	475.4 // 3496.0	391.8 // 6035.8	20.5 // -8.3 // -3.7 // 0.181	-69.6 // 3.9 // 0.7 // 0.855
DNA (D) - RETINOIC ACID RECEPTOR RXR-ALPHA (B)	49 // 260 // 300	45 // 432 // 667	9 // 15 // 15	18 // 81 // 84	421.1 // 3514.4	418.9 // 6035.8	33.7 // -4.9 // -6.3 // 0.599	-69.6 // 4.2 // 0.6 // 0.895
**1FOS**								
**protein-protein**								
cFos (G) - cJun(H)	108 // 396 // 477	115 // 379 // 446	24 // 59 //59	24 // 58 //58	1163.4 // 6309.1	1183.0 // 5993.2	-21.5 // -9.4 // -3.2 // 0.103	-21.2 // -11.0 // -6.2 // 0.171
**DNA - protein**								
DNA (C) - cFos (G)	35 // 334 // 418	34 // 396 // 477	7 // 20 // 20	10 // 59 // 59	318.2 // 4626.8	325.6 // 6309.1	27.4 // -8.1 // -2.9 // 0.115	- 21.5 // 1.0 // -1.0 // 0.771
DNA (D) - cFos (G)	46 // 336 // 396	36 // 396 // 477	8 // 20 // 20	9 // 59 // 59	351.3 // 4493.2	430.0 // 6309.1	45.6 // -5.1 // -6.2 // 0.579	-21.5 // 1.4 // -1.1 // 0.808
DNA (C) - cJun (H)	34 // 334 // 418	30 // 379 // 446	6 // 20 // 20	7 // 58 // 58	296.1 // 4626.8	324.2 // 5993.2	27.4 // -5.4 // -2.8 // 0.262	-21.2 // 2.2 // -1.6 // 0.913
DNA (D) - cJun (H)	31 // 336 // 396	25 // 379 // 446	7 // 20 // 20	10 // 58 // 58	251.7 // 4493.2	254.6 // 5993.2	45.6 // -6.3 // -4.2 // 0.298	-21.2 // 0.3 // -1.3 // 0.767
**1GT0**								
**protein-protein**								
Octamer bidning TF1 (C) - SOX-2 (D)	16 // 738 // 1118	13 // 477 // 647	7 // 134 // 138	4 // 80 // 80	131.1 // 10028.2	128.2 // 6847.8	-102.0 // -0.8 // -0.3 // 0.408	-53.3 // 0.9 // -0.3 // 0.785
**DNA-protein**								
DNA (A) - Octamer binding TF-1 (C)	85 // 408 // 493	84 // 738 // 1118	12 // 24 // 24	28 // 134 // 138	805.2 // 5584.7	787.1 // 10028.2	66.1 // -13.1 // -13.8 // 0.530	-102.0 // 2.0 // -1.8 // 0.852
DNA (B) - Octamer binding TF-1 (C)	81 // 408 // 485	74 // 738 // 1118	11 // 24 // 24	25 // 134 // 138	693.1 // 5397.5	703.8 // 10028.2	37.6 // -7.7 // -7.5 // 0.476	-102.0 // 2.7 // -1.5 // 0.893
DNA (A) - SOX2 (D)	89 // 408 // 493	83 // 477 // 647	10 // 24 // 24	22 // 80 // 80	726.9 // 5584.7	749.1 // 6847.8	66.1 // -4.5 // -14.4 // 0.922	-53.3 // -0.3 // -1.8 // 0.643
DNA (B) - SOX2 (D)	85 // 408 // 485	89 // 477 // 647	9 // 24 // 24	26 // 80 // 80	739.1 // 5397.5	761.0 // 6847.8	37.6 // -0.1 // -7.8 // 0.878	-53.3 // -1.0 // -1.9 // 0.577
**1H8A**								
**protein-protein**								
no direct contact								
**DNA-protein**								
DNA (D) -CAAT/ENHANCER binding protein beta (A)	40 // 444 // 533	34 // 470 // 580	6 // 26 // 26	9 // 68 // 68	356.9 // 5880.4	375.5 // 7075.6	55.8 // -5.3 // -5.0 // 0.460	-23.5 // 3.6 // -1.1 // 0.956
DNA (D) -CAAT/ENHANCER binding protein beta (B)	33 // 444 // 533	33 // 452 // 573	8 // 26 // 26	11 // 67 // 67	307.4 // 5880.4	325.9 // 6729.2	55.8 // -8.7 // -4.1 // 0.144	-25.6 // 0.6 // -0.8 // 0.731
DNA (E) - CAAT/ENHANCER binding protein beta (A)	37 // 436 // 527	28 // 470 // 580	8 // 26 // 26	8 // 68 // 68	336.2 // 5849.0	334.4 // 7075.6	60.1 // -9.3 // -5.1 // 0.179	-23.5 // 1.4 // -0.9 // 0.834
DNA (E) - CAAT/ENHANCER binding protein beta (B)	34 // 435 // 527	30 // 452 // 573	6 // 26 // 26	10 // 67 // 67	300.9 // 5849.00	302.3 // 6729.2	60.1 // -4.5 // -4.7 // 0.497	-25.6 // 2.1 // -0.8 // 0.885
DNA (D) - MYB transforming protein (C)	60 // 444 // 533	67 // 579 // 895	9 // 26 // 26	21 //99 // 105	569.0 // 5880.4	587.1 // 7487.4	55.8 // -11.1 // -7.5 // 0.262	-73.1 // 3.4 // -0.2 // 0.872
DNA (E) - MYB transforming protein (C)	49 // 436 // 527	56 // 579 // 895	8 // 26 // 26	20 // 99 // 105	511.1 // 5849.0	501.5 // 7487.4	60.1 // -12.8 // -6.8 // 0.127	-73.1 // 1.6 // -0.1 // 0.750
**1H9D**								
**protein-protein**								
Core-bnding factor alpha subuinit1 (C) - Core-binding factor cbf-beta (D)	115 // 586 // 956	126 // 646 // 1057	31 // 120 // 125	34 // 124 // 129	1141.0 // 7414.6	1048.0 // 7655.2	-105.3 // -9.1 // -2.6 // 0.043	-114.5 // -2.4 // 1.6 // 0.160
**DNA - protein**								
DNA (H) - Core-bnding factor alpha subuinit1 (C)	39 // 165 // 198	39 // 586 // 956	7 // 10 // 10	13 // 120 // 125	389.5 // 2315.6	372.2 // 7414.6	11.0 // -6.0 // -2.6 // 0.233	-105.3 // 1.6 // -0.9 // 0.866
DNA (G) - Core-bnding factor alpha subuinit1 (C)	36 // 181 // 206	37 // 586 // 956	8 // 10 // 10	10 // 120 // 125	316.8 // 2426.9	365.5 // 7414.6	26.3 // -5.1 // -5.2 // 0.503	-105.3 // 5.2 // -0.8 // 0.993
**1HBX**								
**protein-protein**								
Serum response factor (A) - Ets-domain protein ELK-4 (G)	19 // 517 // 681	20 // 614 // 925	7 // 87 // 87	7 // 108 // 112	179.5 // 7533.6	158.3 // 8199.0	-54.3 // -1.3 // -1.2 // 0.508	-91.5 // -1.9 // -0.7 // 0.224
Serum response factor (B) - Ets-domain protein ELK-4 (G)	101 // 521 // 685	79 // 614 // 925	28 // 86 // 86	17 // 108 // 112	784.9 // 7478.5	864.8 // 8199.0	-59.9 // -5.2 // -5.5 // 0.534	-91.5 // -5.6 // -2.6 // 0.181
**DNA-protein**								
DNA (C) - Serum response factor (A)	64 // 450 // 533	62 // 517 // 681	7 // 26 // 26	16 // 87 // 87	617.5 // 5866.5	573.3 // 7533.6	68.6 // -6.5 // -9.8 // 0.698	-54.3 // 1.6 // -4.1 // 0.958
DNA (C) - Serum response factor (B)	88 // 450 // 533	79 // 521 // 685	14 // 26 // 26	23 // 86 // 86	725.6 // 5866.5	719.2 // 7478.5	68.6 // -12.9 // -13.4 // 0.517	-59.9 // -1.6 // -4.3 // 0.776
DNA (C) - Ets-domain protein ELK-4 (G)	60 // 450 // 533	62 // 614 // 925	12 // 26 // 26	22 // 108 // 112	583.0 // 5866.5	495.3 // 8199.0	68.6 // -12.6 // -9.1 // 0.266	-91.5 // -1.2 // -2.0 // 0.627
DNA (W) - Serum response factor (A)	78 // 448 // 527	64 // 517 // 681	14 // 26 // 26	22 // 87 // 87	638.1 // 5849.5	687.2 // 7533.6	57.7 // -11.4 // -10.1 // 0.402	-54.3 // -1.8 // -4.2 // 0.780
DNA (W) - Serum response factor (B)	58 // 448 // 527	62 // 521 // 685	6 // 26 // 26	16 // 86 // 86	563.1 // 5849.5	527.0 // 7478.5	57.7 // -4.2 // -7.5 // 0.711	-59.9 // 0.2 // -3.4 // 0.861
DNA (W) - Ets-domain protein ELK-4 (G)	62 // 448 // 527	50 // 614 // 925	12 // 26 // 26	14 // 108 // 112	461.6 // 5849.5	472.3 // 8199.0	57.7 // -4.6 // -8.0 // 0.715	-91.5 // 3.5 // -1.6 // 0.974
**1HJB**								
**protein-protein**								
no direct contact								
**DNA-protein**								
DNA (I) - CAAT/ENHANCER binding protein beta (D)	30 // 433 // 532	25 // 461 // 580	7 // 26 // 26	9 // 68 // 68	294.9 // 5800.9	289.7 // 6916.2	63.2 // -7.6 // -4.4 // 0.206	-26.7 // -0.4 // -0.7 // 0.551
DNA (I) - CAAT/ENHANCER binding protein beta (E)	38 // 433 // 532	33 // 448 // 576	7 // 26 // 26	10 // 68 // 68	337.4 // 5800.9	350.7 // 6970.5	63.2 // -4.3 // -5.5 // 0.598	-26.5 // 4.2 // -0.9 // 0.963
DNA (I) - RUNT-related TF (F)	40 // 433 // 532	39 // 558 // 935	9 // 26 // 26	11 // 114 // 120	324.8 // 5800.9	395.3 // 7144.5	63.2 // -6.5 // -5.8 // 0.426	-108.5 // 6.7 // -0.3 // 0.995
DNA (J) - CAAT/ENHANCER binding protein beta (D)	35 // 434 // 528	33 // 461 // 580	6 // 26 // 26	9 // 68 // 68	325.8 // 5742.8	331.0 // 6916.2	57.9 // -4.6 // -4.7 // 0.494	-26.7 // 3.0 // -0.9 // 0.920
DNA (J) - CAAT/ENHANCER binding protein beta (E)	36 // 434 // 528	31 // 448 // 576	7 // 26 // 26	9 // 68 // 68	337.1 // 5742.8	342.7 // 6970.5	57.9 // -10.3 // -4.8 // 0.109	-26.5 // -0.1 // -0.9 // 0.628
DNA (J) - RUNT-related TF (F)	51 // 434 // 528	51 // 558 // 935	9 // 26 // 26	15 // 114 // 120	469.0 // 5742.8	467.2 // 7144.5	57.9 // -8.4 // -6.8 // 0.365	-108.5 // 1.9 // -0.4 // 0.811
**1IO4**								
**protein-protein**								
RUNT-related TF (C) - Core-binding factor beta subunit (D)	109 // 556 // 934	118 // 688 // 1059	29 // 114 // 120	33 // 122 // 128	1087.3 // 7288.7	1062.2 // 7876.2	-110.1 // -8.3 // -0.7 // 0.027	-108.4 // -2.1 // 0.3 // 0.284
CAAT/ENHANCER binding protein beta (A) - Core-binding factor beta subunit (D)	12 // 426 // 538	15 // 688 // 1059	4 // 63 // 63	4 // 122 // 128	107.7 // 6355.5	101.0 // 7876.2	-23.7 // 0.7 // -0.3 // 0.773	-108.4 // -0.2 // 0.0 // 0.487
CAAT/ENHANCER binding protein beta (B) - Core-binding factor beta subunit (D)	32 // 477 // 595	33 // 688 // 1059	10 // 70 // 70	10 // 122 // 128	304.0 // 7279.5	298.2 // 7876.2	-22.6 // 0.5 // -1.2 // 0.758	-108.4 // 2.3 // 0.1// 0.856
**DNA-protein**								
DNA (E) - CAAT/ENHANCER binding protein beta (A)	6 // 439 // 532	4 // 426 // 538	2 // 26 // 26	3 // 63 // 63	38.1 // 5850.6	36.6 // 6355.5	63.4 // 1.6 // -0.9 // 0.307	-23.7 // 0.7 // -0.1 // 0.845
DNA (E) - CAAT/ENHANCER binding protein beta (B)	2 // 439 // 532	4 // 477 // 595	2 // 26 // 26	2 // 70 // 70	35.2 // 5850.6	32.5 // 7279.5	63.4 // -1.9 // -0.3 // 0.094	-22.6 // 0.0 // -0.2 // 0.664
DNA (F) - CAAT/ENHANCER binding protein beta (A)	17 // 430 // 528	20 // 426 // 538	3 // 26 // 26	6 // 63 // 63	114.9 // 5756.3	105.7 // 6355.5	58.9 // -3.0 // -2.3// 0.389	-23.7 // -0.9 // -0.5 // 0.444
DNA (F) -CAAT/ENHANCER binding protein beta (B)	7 // 430 // 528	5 // 477 // 595	3 // 26 // 26	1 // 70 // 70	35.2 // 5756.3	38.5 // 7279.5	58.9 // 0.3 // -1.0 // 0.717	-22.6 // 0.4 // -0.2 // 0.773
DNA (E) - RUNT-related TF (C)	44 // 439 // 532	40 // 556 // 934	8 // 26 // 26	11 // 114 // 120	356.6 // 5850.6	435.1 // 7288.7	63.4 // -8.3 // -6.4 // 0.334	-110.1 // 8.0 // -0.3 // 0.998
DNA (F) - RUNT-related TF (C)	52 // 430 // 528	43 // 556 // 934	9 // 26 // 26	14 // 114 // 120	459.1 // 5756.3	425.0 // 7288.7	58.9 // -7.7 // -7.1 // 0.444	-110.1 // 1.5 // -0.3 // 0.766
**1JEY**								
**protein-protein**								
Ku70 (A) - Ku86 (B)	901 // 2512 // 3982	910 // 2752 // 4242	213 // 458 // 493	207 // 504 // 530	9051.9 // 31906.0	9044.8 // 34448.9	-451.0 // -56.2 // -22. 7 // 0.001	-480.4 // -64.3 // -29.7 // 0.001
**DNA-protein**								
DNA (C) - Ku70 (A)	41 // 311 // 368	37 // 2512 // 3982	7 // 18 // 18	14 // 458 // 493	359.8 // 4231.4	364.5 // 31906.0	55.2 // -3.2 // -7.3 // 0.798	-451 // 2.3 // -0.9 // 0.918
DNA (D) - Ku70 (A)	56 // 523 // 625	57 // 2512 // 3982	11 // 31 // 31	22 // 458 // 493	463.6 // 6581.4	413.2 // 31906.0	41.9 // -9.6 // -4.5 // 0.167	-451.0 // -0.2 // -1.4 // 0.687
DNA (C) -Ku86(B)	52 // 311 // 368	54 // 2752 // 4242	11 // 18 // 18	16 // 504 // 530	473.7 // 4231.4	444.2 // 34448.9	55.2 // -11.8 // -9.2 // 0.313	-480.4 // 0.9 // -1.8 // 0.846
DNA (D) - Ku86(B)	77 // 523 // 625	81 // 2752 // 4242	13 // 31 // 31	26 // 504 // 530	695/5 // 6581.4	672.9 // 34448.9	41.9 // -12.5 // -6.2 // 0.154	-480.4 // 3.6 // -2.6 // 0.968
**1JFI**								
**protein-protein**								
NC2 (A) - TATA- binding protein-associated phosphor protein (B)	174 // 352 // 490	196 // 806 // 1059	44 // 62 // 63	51 // 134 // 135	1993.8 // 11547.3	1969.9 // 5203.6	-72.4 // -24.6 // -9.3 // 0.002	-48.3 // -22.9 // -10.7 // 0.009
NC2 (A) - TATA-box binding protein (C)	23 // 352 // 490	19 // 822/ 1453	7 // 62 // 63	6 // 160 // 183	185.9 // 5203.6	243.0 // 10474.5	-48.3 // -0.4 // -1.4 // 0.710	-189.2 // 0.9 // -0.4 // 0.794
TATA- binding protein-associated phosphor protein (B) - TATA-box binding protein (C)	67 // 806 // 1059	77 // 822 // 1453	20 // 134 // 135	22 // 160 // 183	750.8 // 11547.3	686.7	-189.2 // -3.8 // -1.7 // 0.286	-72.4 // -4.0 // -3.2 // 0.413
**DNA-protein**								
DNA (D) - NC2 (A)	21 // 306 // 369	7 // 352 // 490	6 // 18 // 18	4 // 62 // 63	115.3 // 4199.4	141.8 // 5203.6	38.1 // 0.3 // -2.6 // 0.798	-48.3 // 1.5 // -0.4 // 0.940
DNA (E) - NC2 (A)	16 // 310 // 363	15 // 352 // 490	4 // 18 // 18	6 // 62 // 63	98.1 // 4226.7	110.1 // 5203.6	36.6 // -1.1 // -1.9 // 0.589	-48.3 // 1.1 // -0.9 // 0.896
DNA (D) - TATA- binding protein-associated phosphor protein (B)	41 // 306 // 369	31 // 806 // 1059	6 // 18 // 18	9 // 134 // 135	265.0 // 4199.4	246.3 // 11547.3	38.1 // -4.4 // -5.1 // 0.544	-72.4 // 0.4 // -1.5 // 0.804
DNA (E) - TATA- binding protein-associated phosphor protein (B)	33 // 310 // 363	36 // 806 // 1059	7 // 18 // 18	12 // 134 // 135	298.8 // 4226.7	290.7 // 11547.3	36.6 // -1.4 // -3.9 // 0.713	-72.4 // -1.0 // -1.7 // 0.629
DNA (E) - TATA-box binding protein (C)	86 // 310 // 363	81 // 822 // 1453	10 // 18 // 18	30 // 160 // 183	799.7 // 4226.7	680.5 // 10474.5	36.6 // -5.1 // -10.2 // 0.766	-189.2 // -5.7 // -1.8 // 0.102
DNA (D) - TATA-box binding protein (C)	86 // 306 // 369	74 // 822 // 1453	9 // 18 // 18	29 // 160 // 183	815.4 // 4199.4	707.3 // 10474.5	38.1 // 4.9 // -10.7 // 0.992	-189.2 // -5.6 // -1.6 // 0.084

**1K6O**								
**protein-protein**								
ETS domain protein ELK-4 (A) - Serum response factor (B)	14 // 431 // 745	12 // 481 // 641	5 // 82 // 89	5 // 85 // 85	111.3 // 5428.7	117.9 // 7108.2	-84.2 // -1.4 // -0.5 // 0.229	-52.8 // -0.9 // -0.9 // 0.540
**DNA-protein**								
DNA (D) - ETS domain protein ELK-4 (A)	44 // 396 // 470	44 // 431 // 745	9 // 23 // 23	14 // 82 // 89	386.1 // 5312.4	377.5 // 5428.7	41.6 // -6.3 // - 4.6 // 0.352	-84.2 // 0.5 // -1.6 // 0.847
DNA (E) - ETS domain protein ELK-4 (A)	42 // 391 // 467	51 // 431 // 745	6 // 23 //23	16 // 82 // 89	473.3 // 5273.6	402.4 // 5428.7	53.8 // -5.8 // -5.8 // 0.483	-84.2 // -2.1 // -1.9 // 0.451
DNA (D) - Serum response factor (B)	74 // 396 // 470	55 // 481 // 641	14 // 23 // 23	21 // 85 // 85	575.2 // 5312.4	604.1 // 7108.2	41.6 // -7.5 // -7.8 // 0.502	-52.8 // -0.7 // -4.0 // 0.862
DNA (E) - Serum response factor (B)	64 // 391 // 467	66 // 481 // 641	7 // 23 // 23	16 // 85 // 85	591.5 // 5273.6	565.2 // 7108.2	53.8 // -4.4 // -8.8 // 0.763	-52.8 // 0.4 // -4.8 // 0.934
DNA (D) - Serum response factor (C)	56 // 396 // 470	60 // 484 // 634	7 // 23 // 23	16 // 84 // 84	547.3 // 5312.4	520.2 // 7057.3	41.6 // -4.7 // -5.9 // 0.570	-48.4 // -0.8 // -5.0 // 0.919
DNA (E) - Serum response factor (C)	60 // 391 // 467	52 // 484 // 634	12 // 23 // 23	16 // 84 // 84	510.7 // 5273.6	524.5 // 7057.3	53.8 // -4.0 // -8.2 // 0.763	-48.4 // -1.3 // -4.3 // 0.854
**1K78**								
**protein-protein**								
Pax5 (A) - C-Ets1 (B)	16 // 656 // 964	26 // 512 // 862	4 // 119 // 124	7 // 98 // 102	199.2 // 9042.0	169.6 // 6080.6	95.8 // 0.5 // -0.4 // 0.742	-102.4 // -0.9 // -0.2 // 0.363
Pax5 (I) - C-Ets1 (B)	14 // 306 // 461	16 //512 // 862	4 // 55 // 58	4 // 98 // 102	134.9 // 4479.1	120.8 // 6080.6	-45.5 // -1.9 // -0.3 // 0.120	-102.4 // 0.6 // -0.1 // 0.728
**DNA-protein**								
DNA (C) - Pax5 (A)	103 // 457 // 555	86 // 656 // 964	19 // 27 // 27	28 // 119 // 124	905.9 // 6187.8	914.6 // 9042.0	60.1 // -9.8 // -13.6 // 0.683	-95.8 // -3.2 // -2.0 // 0.366
DNA (C) - Pax5 (I)	44 // 457 // 555	41 // 306 // 461	10 // 27 // 27	15 // 55 // 58	379.4 // 6187.8	367.8 // 4479.1	60.1 // -4.1 // -5.8 // 0.625	-45.5 // 1.1 // -0.7 // 0.772
DNA (D) - Pax5 (A)	104 // 454 // 546	120 // 656 // 964	16 // 27 // 27	40 // 119 // 124	1068.0 // 6048.7	934.2 // 9042.0	48.7 // -9.1 // -11.1 // 0.590	-95.8 // -3.1 // -2.8 // 0.469
DNA (D) - Pax5 (I)	49 // 454 // 546	43 // 306 // 461	8 // 27 // 27	15 // 55 // 58	424.8 // 6084.7	441.2 // 4479.1	48.7 // -5.8 // -5.3 // 0.449	-45.5 // -0.8 // -0.8 // 0.529
DNA (C) - C-Ets1 (B)	51 // 457 // 555	34 // 512 // 862	9 // 27 // 27	10 // 98 // 102	333.1 // 6187.8	366.6 // 6080.6	60.1 // -5.6 // -6.7 // 0.572	-102.4 // 2.9 // -0.2 // 0.927
DNA (D) - C-Ets1 (B)	45 // 454 // 546	62 // 512 // 862	6 // 27 // 26	17 // 98 // 102	493.3 // 6084.7	420.1 // 6080.6	48.7 // -7.8 // -4.8 // 0.258	-102.4 // -1.7 // -0.4 // 0.335
**1LB2**								
**protein-protein**								
Catabolite gene activator protein (A) - DNA-directed RNA polymerase alpha chain (B)	35 // 990 // 1591	34 // 391 // 565	8 // 196 // 201	11 // 71 // 72	316.7 // 11363.1	335.4 // 4856.9	-175.6 // -0.6 // -1.1 // 0.622	-66.1 // -0.7 // -0.6 // 0.507
**DNA-protein**								
DNA (K) - Catabolite gene activator protein (A)	38 // 339 // 403	41 // 990 // 1591	6 // 20 // 20	9 // 196 // 201	366.0 // 4531.2	405.2 // 11363.1	53.2 // -8.6 // -6.0 // 0.278	-175.6 // 2.6 // -1.3 // 0.958
DNA (J) - Catabolite gene activator protein (A)	49 // 403 // 493	46 // 990 // 1591	11 // 24 // 24	20 // 196 // 201	430.1 // 5401.5	396.4 // 11363.1	40.5 // -8.8 // -4.9 // 0.221	-175.6 // 1.8 // -1.5 // 0.921
DNA (K) - DNA-directed RNA polymerase alpha chain (B)	26 // 339 // 403	30 // 391 // 565	4 // 20 // 20	10 // 71 // 72	256.2 // 4531.2	197.8 // 4856.9	53.2 // -5.7 // -4.1 // 0.326	-66.1 // 0.0 // -0.5 // 0.634
DNA (J) -DNA-directed RNA polymerase alpha chain (B)	20 // 403 // 493	20 // 391 // 565	3 // 24 // 24	5 // 71 // 72	168.7 // 5401.5	160.5 // 4856.9	40.5 // -0.0 // -2.0 // 0.715	-66.1 // 0.3 // -0.4 // 0.681
DNA (K) - DNA-directed RNA polymerase alpha chain (E)	25 // 339 // 403	28 // 364 // 507	4 // 20 // 20	10 // 65 // 66	261.0 // 4531.2	207.0 // 4306.2	53.2 // -6.4 // -3.9 // 0.246	-58.0 // 0.3 // -0.6 // 0.718
DNA (J) - DNA-directed RNA polymerase alpha chain (E)	23 // 403 // 493	21 // 364 // 507	4 // 24 // 23	5 // 65 // 66	179.3 // 5401.5	197.7 // 4306.2	40.5 // -1.8 // -2.3 // 0.538	-58.0 // -0.4 // -0.5 // 0.565
**1LE5**								
**protein-protein**								
Nuclear factor NF-kappa-B p65 subunit (A) - Nuclear factor NF-kappa-B p105 factor (B)	71 // 1264 // 2184	76 // 1468 // 2462	19 // 263 // 274	18 // 303 // 313	704.2 // 14395.9	709.2 // 16293.7	-236.1 // -2.6 // -0.3 // 0.224	-286.5 // -1.5 // -1.3 // 0.479
**DNA-protein**								
DNA (C) - Nuclear factor NF-kappa-B p65 subunit (A)	61 // 207 // 244	71 // 1264 // 2184	8 // 12 // 12	22 // 263 // 274	551.2 // 2819.1	542.1 // 14395.9	26.6 // -11.4 // -7.8 // 0.265	-236.1 // 0.3 // -0.3 // 0.592
DNA (D) - Nuclear factor NF-kappa-B p65 subunit (A)	24 // 205 // 242	24 // 1264 // 2184	6 // 12 // 12	10 // 263 // 274	215.5 // 2820.9	247.9 // 14395.9	20.1 // -2.7 // -2.3 // 0.442	-236.1 // 2.5 // -0.1 // 0.926
DNA (C) - Nuclear factor NF-kappa-B p105 factor (B)	37 // 208 // 244	39 // 1468 // 2462	10 // 12 // 12	14 // 303 // 313	317.6 // 2819.1	325.8 // 16293.7	26.6 // -4.7 // -4.7 // 0.488	-286.5 // 2.8 // -0.6 // 0.941
DNA (D) - Nuclear factor NF-kappa-B p105 factor (B)	70 // 205 // 242	85 // 1468 // 2462	9 // 12 // 12	24 // 303 // 313	747.8 // 2820.9	684.5 // 16293.7	20.1 // -14.9 // -6.9 // 0.102	-286.5 // -1.0 // -1.4 // 0.560
**1LE8**								
**protein-protein**								
Mating type protein A-1 (A) - Mating type protein alpha-2 (B)	62 // 284 // 414	56 // 409 // 579	16 // 52 // 53	15 // 71 // 74	607.6 // 3926.6	659.1 // 5939.0	-34.6 // -1.1 // -2.2 // 0.656	-48.4 // -5.3 // -2.7 // 0.187
**DNA-protein**								
DNA (C) - Mating type protein A-1 (A)	42 // 340 // 406	36 // 284 // 414	8 // 20 // 20	11 // 52 // 53	361.1 // 4541.9	412.8 // 3926.6	33.4 // -8.0 // -4.1 // 0.205	-34.6 // -0.1 // -1.3 // 0.702
DNA (D) - Mating type protein A-1 (A)	32 // 341 // 408	33 // 284 // 414	6 // 20 // 20	12 // 52 // 53	316.8 // 4608.3	323.9 // 3926.6	55.3 // -6.4 // -5.2 // 0.374	-34.6 // 1.5 // -1.2 // 0.876
DNA (C) - Mating type protein alpha-2 (B)	45 // 340 // 406	34 // 409 // 579	10 // 20 // 20	12 // 71 // 74	330.3 // 4541.9	337.2 // 5939.0	33.4 // -4.7 // -4.4 // 0.466	-48.4 // 1.8 // -1.6 // 0.931
DNA (D) - Mating type protein alpha-2 (B)	46 // 341 // 408	48 // 409 // 579	7 // 20 // 20	13 // 71 // 74	441.4 // 4608.3	421.1 // 5939.0	55.3 // -7.8 // -7.5 // 0.462	-48.4 // -0.2 // -2.3 // 0.792
**1MDM**								
**protein-protein**								
Paired box protein PAX-5 (A) - C-ETS-1 protein (B)	15 // 663 // 964	21 // 614 // 1030	4 // 120 // 124	6 // 125 // 129	188.7 // 8919.3	171.6 // 7245.4	-93.6 // 0.3 // -0.4 // 0.719	-116.4 // -0.5 // -0.5 // 0.528
**DNA-protein**								
DNA (C) - Paired box protein PAX-5 (A)	103 // 440 // 533	90 // 663 // 964	18 // 26 // 26	29 // 120 // 124	905.0 // 5898.5	926.1 // 8919.3	56.1 // -8.4 // -13.1 // 0.731	-93.6 // -2.9 // -2.5 // 0.446
DNA (D) - Paired box protein PAX-5 (A)	117 // 442 // 527	126 // 663 // 964	17 // 26 // 26	42 // 120 // 124	1053.1 // 5849.4	958.5 // 8919.3	43.2 // -8.4 // -11.4 // 0.635	-93.6 // -1.8 // -3.5 // 0.663
DNA (C) - C-ETS-1 protein (B)	59 // 440 // 533	43 // 614 // 1030	10 // 26 // 26	13 // 125 // 129	391.4 // 5898.5	442.7 // 7245.4	56.1 // -5.8 // -7.5 // 0.613	-116.4 // 3.2 // -1.1 // 0.964
DNA (D) - C-ETS-1 protein (B)	46 // 442 // 527	61 // 614 // 1030	6 // 26 // 26	16 // 125 // 129	507.5 // 5849.4	426.4 // 7245.4	43.2 // -7.5 // -4.5 // 0.263	-116.4 // -1.0 // -1.6 // 0.585
**1MNM**								
**protein-protein**								
MCM1 (A) - MAT ALPHA-2 (D)	79 // 521 // 681	59 // 463 // 631	22 // 85 // 85	12 // 76 // 77	611.4 // 7511.9	735.5 // 6389.4	-57.4 // -3.1 // -3.8 // 0.594	-52.3 // -5.8 // -1.4 // 0.060
MCM1 (B) - MAT ALPHA-2 (C)	90 // 468 // 623	64 // 436 // 609	25 // 81 // 81	13 // 76 // 77	651.1 // 6695.5	742.5 // 6024.7	-51.9 // -4.0 // -7.0 // 0.792	-43.6 // -4.9 // -3.0 // 0.269
**DNA-protein**								
DNA (E) - MCM1 (A)	60 // 442 // 533	43 // 521 // 681	14 // 26 // 26	13 // 85 // 85	478.9 // 5926.4	437.9 // 7511.9	51.8 // -6.6 // -7.0 // 0.518	-57.4 // 2.9 // -2.1 // 0.972
DNA (E) - MCM1 (B)	39 // 442 // 533	35 // 468 // 623	8 // 26 // 26	11 // 81 // 81	367.7 // 5926.4	344.2 // 6695.5	51.8 // -4.3 // -4.6 // 0.508	-51.9 // -0.1 // -2.7 // 0.872
DNA (F) - MCM1 (A)	48 // 445 // 527	54 // 521 // 681	6 // 26 // 26	14 // 85 // 85	460.8 // 5916.8	427.8 // 7511.9	60.4 // -4.8 // -6.5 // 0.614	-57.4 // 1.1 // -2.6 // 0.900
DNA (F) - MCM1 (B)	58 // 445 // 527	59 // 468 // 623	12 // 26 // 26	19 // 81 // 81	575.8 // 5916.8	536.0 // 6695.5	60.4 // -14.8 // -7.9 // 0.112	-51.9 // 0.2 // -4.6 // 0.944
DNA (E) - MAT ALPHA-2 (C)	57 // 442 // 533	72 // 436 // 609	7 // 26 // 26	15 // 76 // 77	554.1 // 5926.4	533.6 // 6024.7	51.8 // -8.1 // -6.7 // 0.386	-43.6 // 0.3 // -3.4 // 0.878
DNA (F) - MAT ALPHA-2 (C)	64 // 445 // 527	58 // 436 // 609	11 // 26 // 26	13 // 76 // 77	538.8 // 5916.8	585.4 // 6024.7	60.4 // -4.8 // -8.7 // 0.737	-43.6 // 3.6 // -2.7 // 0.983
**1N6J**								
**protein-protein**								
Myocyte-specific enhancer factor 2B (A) - Calcineurin-binding protein Cabin 1 (G)	52 // 608 // 768	51 // 151 // 186	12 // 93 // 93	15 // 26 // 26	454.8 // 9033.2	436.6 // 2411.2	-64.4 // -2.4 // -1.5 // 0.372	-11.4 // -3.1 // -3.0 // 0.422
Myocyte-specific enhancer factor 2B (B) - Calcineurin-binding protein Cabin 1 (G)	48 // 590 // 751	51 // 151 // 186	14 // 90 // 90	14 // 26 // 26	520.6 // 8763.9	503.2 // 2411.2	-62.2 // -2.8 // -1.3 // 0.284	-11.3 // -3.3 // -3.0 // 0.424
**DNA-protein**								
DNA (C) - Myocyte-specific enhancer factor 2B (A)	43 // 238 // 284	34 // 608 // 768	11 // 14 // 14	9 // 93 // 93	288.4 // 3303.8	308.0 // 9033.2	32.1 // -2.7 // -5.8 // 0.728	-64.4 // 0.5 // -0.9 // 0.736
DNA (D) - Myocyte-specific enhancer factor 2B (A)	51 // 235 // 284	53 // 608 // 768	6 // 14 // 14	14 // 93 // 93	482.1 // 3303.4	451.8 // 9033.2	30.6 // -4.7 // -6.7 // 0.635	-64.4 // -0.3 // -1.5 // 0.664
DNA (C) - Myocyte-specific enhancer factor 2B (B)	58 // 238 // 284	57 // 590 // 751	8 // 14 // 14	15 // 90 // 90	497.8 // 3303.8	470.2 // 8763.9	32.1 // -6.6 // -7.8 // 0.573	-62.2 // -0.1 // -1.6 // 0.687
DNA (D) - Myocyte-specific enhancer factor 2B (B)	45 // 235 // 284	35 // 590 // 751	11 // 14 // 14	9 // 90 // 90	280.1 // 3303.4	322.1 // 8763.9	30.6 // -1.8 // -5.9 // 0.790	-62.2 // 1.0 // -1.0 // 0.793
**1NGM**								
**protein-protein**								
TF IID (A) - TF IIIB BRF subunit (B)	203 // 833 // 1416	193 // 498 // 586	46 // 163 // 180	46 // 72 // 72	1853.1 // 10430.0	1925.8 // 7749.2	-191.1 // 0.3 // -4.5 // 0.810	-30.3 // -9.8 // -10.9 // 0.583
**DNA-protein**								
DNA (C) - TF IID (A)	94 // 325 // 386	88 // 833 // 1416	10 // 19 // 19	31 // 163 // 180	843.4 // 4409.6	741.9 // 10430.0	40.3 // 5.6 // -11.7 // 0.994	-191.1 // -6.0 // -1.9 // 0.099
DNA (D) - TF IID (A)	91 // 331 // 387	100 // 833 // 1416	9 // 19 // 19	30 // 163 // 180	829.9 // 4489.2	778.6 // 10430.0	39.6 // -8.6 // -10.9 // 0.618	-191.1 // -5.8 // -2.2 // 0.148
DNA (C) - TF IIIB BRF subunit (B)	3 // 325 // 386	4 // 498 // 586	1 // 19 // 19	3 // 72 // 72	33.9 // 4409.6	31.3 // 7749.2	40.3 // -1.5 // -0.4 // 0.191	-30.3 // -0.1 // -0.2 // 0.629
**1NH2**								
**protein-protein**								
TF IID (A) - TF IIA (C)	72 // 800 // 1416	61 // 334 // 419	17 // 157 // 180	12 // 50 // 50	538.8 // 10328.3	610.1 // 4985.3	-190.9 // 1.7 // -1.6 // 0.848	-36.0 // -4.6 // -2.6 // 0.235
TF IID (A) - TF IIA (D)	51 // 800 // 1416	37 // 585 // 807	13 // 157 // 180	11 // 104 // 104	383.1 // 10328.3	435.8 // 7982.0	-190.9 // 0.9 // -1.1 // 0.779	-74.9 // -3.9 // -1.6 // 0.147
**DNA-protein**								
DNA (E) - TF IID (A)	79 // 254 // 287	75 // 800 // 1416	7 // 14 // 14	28 // 157 // 180	764.2 // 3788.0	653.1 // 10328.3	15.7 // 7.5 // -4.9 // 0.973	-190.9 // -5.0 // -1.7 // 0.115
DNA (F) - TF IID (A)	86 // 281 // 323	88 // 800 // 1416	9 // 16 // 16	29 // 157 // 180	815.2 // 3812.8	688.1 // 10328.3	34.3 // -5.8 // -10.5 // 0.748	-190.9 // -6.1 // -2.0 // 0.087
DNA (E) - TF IIA (C)	6 // 254 // 287	11 // 334 // 419	3 // 14 // 14	4 // 50 // 50	69.1 // 3788.0	61.2 // 4985.3	15.7 // -2.1 // -0.4 // 0.178	-36.0 // 0.6 // -0.5 // 0.803
DNA (F) - TF IIA (C)	17 // 281 // 323	7 // 334 // 419	4 // 16 // 16	2 // 50 // 50	102.8 // 3812.8	110.8 // 4985.3	34.3 // -3.9 // -2.1 // 0.268	-36.0 // 1.4 // -0.3 // 0.932
**1NKP**								
**protein-protein**								
Myc proto-oncogene protein (A) - Max protein (B)	161 // 562 // 733	163 // 539 // 686	38 // 88 // 88	40 // 83 // 83	1681.7 // 8180.4	1673.7 // 7607.8	-42.8 // -15.4 // -5.8 // 0.039	-35.0 // -14.3 // -3.0 // 0.013
**DNA-protein**								
DNA (F) - Myc proto-oncogene protein (A)	30 // 321 // 385	25 // 562 // 733	7 // 19 // 19	9 // 88 // 88	254.0 // 4370.1	263.7 // 8180.4	35.3 // -3.8 // -3.3 // 0.440	-42.8 // -0.4 // -0.9 // 0.611
DNA (G) - Myc proto-oncogene protein (A)	55 // 330 // 385	55 // 562 // 733	8 // 19 // 19	14 // 88 // 88	495.7 // 4457.5	503.3 // 8180.4	34.0 // -12.2 // -5.7 // 0.122	-42.8 // 0.6 // -2.0 // 0.797
DNA (F) - Max protein (B)	54 // 321 // 385	47 // 539 // 686	8 // 19 // 19	12 // 83 // 83	462.6 // 4370.1	458.7 // 7607.8	35.3 // -11.0 // -5.9 // 0.182	-35.0 // -1.9 // -0.9 // 0.365
DNA (G) - Max protein (B)	36 // 330 // 385	28 // 539 // 686	8 // 19 // 19	8 // 83 // 83	260.2 // 4457.5	272.7 // 7607.8	34.0 // -3.8 // -3.7 // 0.475	-35.0 // -1.6 // -0.5 // 0.321
**1NLW**								
**protein-protein**								
Mad protein (A) - Max protein (B)	131 // 491 // 634	148 // 479 // 637	35 // 79 // 79	36 // 76 // 76	1501.0 // 7326.1	1480.9 // 6829.1	-26.4 // -15.0 // -6.7 // 0.042	-38.1 // -10.9 // -1.5 // 0.026
**DNA-protein**								
DNA (F) - Mad protein (A)	56 // 304 // 366	44 // 491 // 634	8 // 18 // 18	11 // 79 // 79	469.2 // 4180.1	449.7 // 7326.1	39.6 // -10.9 // -7.3 // 0.260	-26.4 // 1.0 // -2.3 // 0.866
DNA (G) - Mad protein (A)	28 // 302 // 366	19 // 491 // 634	6 // 18 // 18	7 // 79 // 79	203.7 // 4153.6	222.1 // 7326.1	35.6 // -1.7 // -3.3 // 0.643	-26.4 // -1.7 // -1.0 // 0.360
DNA (F) - Max protein (B)	36 // 304 // 366	28 // 479 // 637	7 // 18 // 18	8 // 76 // 76	274.7 // 4180.1	297.0 // 6829.1	39.6 // -3.1 // -4.7 // 0.621	-38.1 // 0.3 // -0.3 // 0.635
DNA (G) - Max protein (B)	54 // 302 // 366	49 // 479 // 637	8 // 18 // 18	12 // 76 // 76	486.0 // 4153.6	487.8 // 6829.1	35.6 // -12.7 // -6.4 // 0.130	-38.1 // 0.3 // -0.5 // 0.617
**1O4X**								
**protein-protein**								
Oct1 (A) - Sox2 (B)	27 // 669 // 1039	25 // 493 // 664	10 // 127 // 129	8 // 77 // 77	219.8 // 8953.7	252.7 // 7148.1	-100.9 // 0.1 // -0.7 // 0.683	-62.0 // -1.2 // 0.0 // 0.330
**DNA-protein**								
DNA (C) - Oct1 (A)	63 // 324 // 385	64 // 669 // 1039	10 // 19 // 19	22 // 127 // 129	621.2 // 4454.9	602.3 // 8953.7	51.9 // -13.1 // -10.1 // 0.297	-100.9 // 1.2 // -1.7 // 0.827
DNA (D) - Oct1 (A)	44 // 319 // 388	43 // 669 // 1039	8 // 19 // 19	17 // 127 // 129	446.5 // 4486.7	426.5 // 8953.7	23.7 // -6.6 // -3.3 // 0.245	-100.9 // 2.5 // -1.1 // 0.918
DNA (C) - Sox2 (B)	76 // 324 // 385	63 // 493 // 664	10 // 19 // 19	19 // 77 // 77	634.5 // 4454.9	664.8 // 7148.1	51.9 // -3.0 // -12.2 // 0.925	-62.0 // -0.3 // 0.1 // 0.474
DNA (D) -Sox2 (B)	73 // 319 // 388	85 // 493 // 664	9 // 19 // 19	26 // 77 // 77	742.7 // 4486.7	770.3 // 7148.1	23.7 // 2.1 // -5.4 // 0.885	-62.0 // -3.4 // 0.1 // 0.207
**1OUZ**								
**protein-protein**								
Integration host factor alpha subunit (A) - Integration host factor beta subunit (B)	249 // 594 // 768	240 // 569 // 745	56 // 96 // 96	55 // 94 // 94	2377.6 // 8255.1	2414.3 // 8069.1	-61.0 // -18.8 // -5.5 // 0.015	-62.9 // -18.9 // -8.8 // 0.046
**DNA-protein**								
DNA (C) - Integration host factor alpha subunit (A)	85 // 611 // 716	66 // 594 // 768	15 // 35 // 35	20 // 96 // 96	642.1 // 8130.9	681.9 // 8255.1	74.9 // -4.9 // -10.4 // 0.790	-61.0 // 0.9 // -1.5 // 0.759
DNA (D) - Integration host factor alpha subunit (A)	20 // 257 // 307	25 // 594 // 768	3 // 15 // 15	12 // 96 // 96	211.3 // 3420.5	178.6 // 8255.1	22.3 // -5.4 // -1.7 // 0.140	-61.0 // -0.1 // -0.6 // 0.626
DNA (C)- Integration host factor beta subunit (B)	105 // 611 // 716	71 // 569 // 745	14 // 35 // 35	25 // 94 // 94	778.7 // 8130.9	779.9 // 8069.1	74.9 // -9.1 // -12.9 // 0.687	-62.9 // 0.3 // -2.6 // 0.790
DNA (D) - Integration host factor beta subunit (B)	18 // 257 // 307	11 // 569 // 745	5 // 15 // 15	3 // 94 // 94	109.8 // 3420.5	98.6 // 8069.1	22.3 // -1.8 // -1.6 // 0.448	-62.9 // -0.9 // -0.4 // 0.314
DNA(E) - Integration host factor alpha subunit (A)	74 // 343 // 403	62 // 594 // 768	9 // 20 // 20	21 // 96 // 96	613.2 // 4633.6	603.9 // 8255.1	38.6 // -4.9 // -8.3 // 0.697	-61.0 // -0.3 // -1.4 // 0.629
DNA (E)-Integration host factor beta subunit (B)	38 // 343 // 403	34 // 569 // 745	7 // 20 // 20	10 // 94 // 94	338.9 // 4633.6	327.1 // 8069.1	38.6 // -1.8 // -4.3 // 0.695	-62.9 // 0.6 // -1.2 // 0.785
**1PUF**								
**protein-protein**								
Homeobox protein HoxA9 (A) - Pre-B-cell leukemia transcription factor 1 (B)	34 // 477 // 645	43 // 405 // 596	7 // 76 // 77	12 // 71 // 73	395.1 // 6870.5	346.5 // 5461.8	-47.0 // -3.6 // -1.3 // 0.179	-51.6 // -0.4 // -0.3 // 0.509
**DNA-protein**								
DNA (D) - Homeobox protein HoxA9 (A)	72 // 337 // 403	72 // 477 // 645	8 // 20 // 20	17 // 76 // 77	661.3 // 4602.7	638.4 // 6870.5	44.3 // -10.4 // -9.5 // 0.434	-47.0 // 2.4 // -2.7 // 0.913
DNA (E)- Homeobox protein HoxA9 (A)	84 // 338 // 411	72 // 477 // 645	12 // 20 // 20	18 // 76 // 77	686.2 // 4599.5	702.1 // 6870.5	37.8 // -7.8 // -9.4 // 0.584	-47.0 // 1.6 // -2.7 // 0.875
DNA (D)- Pre-B-cell leukemia transcription factor 1 (B)	43 // 337 // 403	47 // 405 // 596	5 // 20 // 20	10 // 71 // 73	392.6 // 4602.7	416.0 // 5461.8	44.3 // -2.1 // -5.6 // 0.759	-51.6 // 2.4 // -0.4 // 0.841
DNA (E) - Pre-B-cell leukemia transcription factor 1 (B)	63 // 338 // 411	55 // 405 // 596	12 // 20 // 20	17 // 71 // 73	547.4 // 4599.5	511.9 // 5461.8	37.8 // -11.7 // -7.1 // 0.213	-51.6 // 3.9 // -0.4 // 0.922
**1R0O**								
**protein-protein**								
Ultraspiracle protein (A) - Ecdysone receptor (B)	34 // 380 // 611	33 // 392 // 609	9 // 72 // 76	8 // 74 // 78	294.9 // 5090.4	284.5 // 5132.0	-60.7 // 1.5 // 0.6 // 0.676	-71.3 // -1.7 // -0.7 // 0.325
**DNA-protein**								
DNA (C) - Ultraspiracle protein (A)	46 // 312 // 369	46 // 380 // 611	7 // 18 // 18	17 // 72 // 76	429.1 // 4268.9	373.5 // 5090.4	38.2 // -10.3 // -5.6 // 0.184	-60.7 // 3.0 // 0.8 // 0.793
DNA (D) - Ultraspiracle protein (A)	38 // 307 // 368	38 // 380 // 611	6 // 18 // 18	14 // 72 // 76	364.3 // 4207.3	356.3 // 5090.4	41.7 // -5.6 // -5.2 // 0.454	-60.7 // 2.6 // 0.7 // 0.786
DNA (C) - Ecdysone receptor (B)	40 // 312 // 369	40 // 392 // 609	6 // 18 // 18	14 // 74 // 78	397.3 // 4268.9	369.8 // 5132.0	38.2 // -5.6 // -4.9 // 0.432	-71.3 // 5.1 // -0.9 // 0.982
DNA (D) - Ecdysone receptor (B)	47 // 307 // 368	47 // 392 // 609	9 // 18 // 18	19 // 74 // 78	424.0 // 4207.3	419.7 // 5132.0	41.7 // -8.2 // -6.4 // 0.356	-71.3 // 3.3 // -1.0 // 0.928
**1RIO**								
**protein-protein**								
SigA (H) - Repressor protein CI (B)	18 // 324 // 483	19 // 506 // 753	5 // 58 // 61	6 // 92 // 96	156.6 // 4347.0	198.0 // 6604.0	-44.0 // 1.5 // -0.3 // 0.866	-70.8 // 0.7 // -0.6 // 0.813
**DNA-protein**								
DNA (U) - SigA (H)	31 // 459 // 550	26 // 324 // 483	7 // 27 // 27	8 // 58 // 61	283.4 // 6167.7	266.0 // 4347.0	54.6 // -6.0 // -3.7 // 0.281	-44.0 // 0.4 // -0.5 // 0.682
DNA (T) - SigA (H)	36 // 453 // 551	35 // 324 // 483	6 // 27 // 27	10 // 58 // 61	335.8 // 6125.0	345.8 // 4347.0	51.0 // -5.1 // -4.1 // 0.393	-44.0 // 0.4 // -0.7 // 0.683
DNA (U) - Repressor protein CI (A)	46 // 459 // 550	45 // 521 // 764	10 // 27 // 27	15 // 92 // 97	436.8 // 6167.7	414.9 // 6673.5	54.6 // -5.7 // -5.5 // 0.470	-76.9 // 0.5 // -0.9 // 0.733
DNA (T) - Repressor protein CI (A)	56 // 453 // 551	51 // 521 // 764	9 // 27 // 27	17 // 92 // 97	510.3 // 6125.0	534.0 // 6673.5	51.0 // -9.0 // -6.3 // 0.304	-76.9 // 0.4 // -1.1 // 0.727
DNA (U) - Repressor protein CI (B)	38 // 459 // 550	41 // 506 // 753	7 // 27 // 27	16 // 92 // 96	374.7 // 6167.7	353.2 // 6604.0	54.6 // -7.8 // -4.5 // 0.227	-70.8 // -1.2 // -1.4 // 0.550
DNA (T) - Repressor protein CI (B)	48 // 453 // 551	42 // 506 // 753	8 // 27 // 27	15 // 92 // 96	403.6 // 6125.0	377.6 // 6604.0	51.0 // -8.6 // -5.4 // 0.262	-70.8 // 1.7 // -1.4 // 0.903
**1RZR**								
**protein-protein**								
Glucose-resistance amylase regulator (A) - Phosphocarrier protein HPr (S)	74 // 1490 // 2562	71 // 385 // 632	22 // 296 // 332	18 // 83 // 87	652.7 // 16221.6	693.2 // 4618.0	-282.2 // -2.6 // -1.4 // 0.358	-72.7 // -8.4 // -2.3 // 0.017
Glucose-resistance amylase regulator (A) - Phosphocarrier protein HPr (Y)	3 // 1490 // 2562	3 // 387 // 632	1 // 296 // 332	1 // 84 // 87	26.8 // 16221.6	26.6 // 4661.7	-282.2 // 0.2 // -0.1 // 0.775	-71.5 // 0.6 // -0.1 // 0.916
Glucose-resistance amylase regulator (D) - Phosphocarrier protein HPr (S)	8 // 1460 // 2558	4 // 385 // 632	2 // 293 // 332	1 // 83 // 87	45.0 // 16554.9	45.4 // 4618.0	-284.5 // 0.2 // -0.2 // 0.723	-72.7 // 0.9 // -0.1 // 0.938
Glucose-resistance amylase regulator (D) - Phosphocarrier protein HPr (Y)	75 // 1460 // 2558	76 // 387 // 632	24 // 293 // 332	19 // 84 // 87	676.8 // 16554.9	742.8 // 4661.7	-284.5 // -2.1 // -1.5 // 0.431	-71.5 // -9.9 // -2.9 // 0.011
**DNA-protein**								
DNA(E) - Glucose-resistance amylase regulator (A)	53 // 276 // 327	51 // 1490 // 2562	9 // 16 // 16	14 // 296 // 332	470.9 // 3697.2	435.7 // 16221.6	22.6 // -4.0 // -4.3 // 0.514	-282.2 // -1.7 // -1.0 // 0.318
DNA (B) - Glucose-resistance amylase regulator (A)	59 // 279 // 323	57 // 1490 // 2562	8 // 16 // 16	21 // 296 // 332	516.1 // 3720.2	461.9 // 16221.6	43.6 // -7.6 // -9.2 // 0.605	-282.2 // -3.3 // -1.1 // 0.148
DNA (E) - Glucose-resistance amylase regulator (D)	62 // 276 // 327	59 // 1460 // 2558	8 // 16 // 16	20 // 293 // 332	565.8 // 3697.2	485.8 // 16554.9	22.6 // -4.0 // -5.1 // 0.563	-284.5 // -1.6 // -1.2 // 0.402
DNA (B) - Glucose-resistance amylase regulator (D)	50 // 279 // 323	46 // 1460 // 2558	9 // 16 // 16	15 // 293 // 332	443.6 // 3720.2	422.3 // 16554.9	43.6 // -6.0 // -7.8 // 0.630	-284.5 // -2.7 // -0.9 // 0.177

**1T2K**								
**protein-protein**								
Interferon regulatory factor 3 (B) - Transcription factor AP-1 (C)	4 // 580 // 888	6 // 412 // 484	2 // 106 // 108	2 // 62 // 62	37.0 // 6964.2	34.5 // 6419.7	-85.2 // -0.5 // -0.1 // 0.266	-24.2 // 0.4 // -0.3 // 0.766
Interferon regulatory factor 3 (A) - Cyclic-AMP-dependent transcription (D)	24 // 573 // 912	18 // 389 // 477	5 // 107 // 110	6 // 61 // 61	170.9 // 7108.0	176.8 // 6036.7	-89.1 // -0.4 // -0.2 // 0.483	-18.4 // 1.8 // -0.8 // 0.9
**DNA-protein**								
DNA (E)- Interferon regulatory factor 3 (A)	49 // 533 // 649	48 // 573 // 912	9 // 31 // 31	15 // 107 // 110	457.2 // 7009.4	421.9 // 7108.0	53.9 // -4.7 // -5.0 // 0.507	-89.1 // 0.3 // -0.5 // 0.637
DNA (F)- Interferon regulatory factor 3 (A)	76 // 527 // 616	72 // 573 // 912	11 // 31 // 31	20 // 107 // 110	710.7 // 6909.0	658.6 // 7108.0	104.3 // -12.7 // -15.0 // 0.632	-89.1 // 0.7 // -0.7 // 0.688
DNA (E) - Interferon regulatory factor 3 (B)	61 // 533 // 649	55 // 580 // 888	12 // 31 // 31	17 // 106 // 108	531.9 // 7009.4	512.5 // 6964.2	53.9 // -7.2 // -6.2 // 0.415	-85.2 // 0.4 // -0.7 // 0.680
DNA (F) - Interferon regulatory factor 3 (B)	76 // 527 // 616	71 // 580 // 888	11 // 31 // 31	21 // 106 // 108	638.9 // 6909.0	595.3 // 6964.2	104.3 // -13.7 // -15.0 // 0.570	-85.2 // -1.4 // -1.0 // 0.446
DNA (E) - Transcription factor AP-1 (C)	36 // 533 // 649	26 // 412 // 484	5 // 31 // 31	8 // 62 // 62	255.9 // 7009.4	277.7 // 6419.7	53.9 // -3.8 // -3.6 // 0.464	-24.2 // 0.1 // -1.1 // 0.698
DNA (F) - Transcription factor AP-1 (C)	8 // 527 // 616	8 // 412 // 484	4 // 31 // 31	5 // 62 // 62	58.0 // 6909.0	59.0 // 6419.7	104.3 // -0.9 // -1.6 // 0.597	-24.2 // 0.3 // -0.3 // 0.712
DNA (E) - Cyclic-AMP-dependent transcription (D)	36 // 533 // 649	29 // 389 // 477	7 // 31 // 31	9 // 61 // 61	309.8 // 7009.4	291.8 // 6036.7	53.9 // -7.3 // -3.6 // 0.199	-18.4 // 1.8 // -1.3 // 0.890
DNA (F) - Cyclic-AMP-dependent transcription (D)	37 // 527 // 616	29 // 389 // 477	6 // 31 // 31	10 // 61 // 61	325.1 // 6909.0	352.2 // 6036.7	104.3 // -5.9 // -7.3 // 0.610	-18.4 // 0.7 // -1.3 // 0.795
**1TQE**								
**protein-protein**								
Myocyte-specific enhancer factor 2B (R) - Histone deacetylase 9 (Y)	34 // 585 // 751	33 // 150 // 172	9 // 90 // 90	10 // 23 // 23	340.8 // 8682.8	370.6 // 2552.4	-55.6 // -2.3 // -1.3 // 0.341	-8.8 // -4.0 // -2.6 // 0.243
Myocyte-specific enhancer factor 2B (S) - Histone deacetylase 9 (Y)	69 // 591 // 751	60 // 150 // 172	16 // 90 // 90	14 // 23 // 23	557.5 // 8734.4	587.1 // 2552.4	-54.2 // -2.4 // -2.8 // 0.552	-8.8 // -5.0 // -4.7 // 0.438
**DNA-protein**								
DNA (E) - Myocyte-specific enhancer factor 2B (R)	57 // 289 // 347	50 // 585 // 751	6 // 17 // 17	15 // 90 // 90	498.7 // 3884.6	501.5 // 8682.8	31.5 // -5.9 // -6.2 // 0.508	-55.6 // 0.3 // -2.0 // 0.783
DNA (F) - Myocyte-specific enhancer factor 2B (R)	61 // 286 // 344	47 // 585 // 751	12 // 17 // 17	14 // 90 // 90	405.9 // 3914.2	420.5 // 8682.8	46.9 // -1.9 // -10.0 // 0.926	-55.6 // -1.3 // -1.8 // 0.592
DNA (E) - Myocyte-specific enhancer factor 2B (S)	46 // 289 // 347	36 // 591 // 751	11 // 17 // 17	11 // 90 // 90	291.4 // 3884.6	313.6 // 8734.4	31.5 // -3.1 // -5.0 // 0.641	-54.2 // -0.2 // -1.5 // 0.709
DNA (F) - Myocyte-specific enhancer factor 2B (S)	54 // 286 // 344	50 // 591 // 751	6 // 17 // 17	14 // 90 // 90	501.2 // 3914.2	500.3 // 8734.4	46.9 // -5.1 // -8.9 // 0.752	-54.2 // 0.5 // -2.0 // 0.807
**1X9M**								
**protein-protein**								
DNA polymerase (A) - Thioredoxin 1 (B)	107 // 2999 // 5251	111 // 471 // 790	32 // 626 // 675	28 // 98 // 105	1066.8 // 31700.8	1064.0 // 5601.6	-614.9 // -9.1 // -3.2 // 0.029	-107.3 // -6.3 // -3.6 // 0.226
**DNA-protein**								
DNA (C) - DNA polymerase (A)	69 // 160 // 190	73 // 2999 // 5251	8 // 9 // 9	29 // 626 // 675	670.4 // 2287.4	606.3 // 31700.8	20.8 // -8.8 // -9.0 // 0.501	-614.9 // 1.6 // -2.2 // 0.923
DNA (D) - DNA polymerase (A)	88 // 190 // 219	114 // 2999 // 5251	10 // 11 // 11	40 // 626 // 675	968.4 // 2655.9	834.7 // 31700.8	16.3 // -10.6 // -7.6 // 0.334	-614.9 // -0.2 // -3.4 // 0.842
**1XS9**								
**protein-protein**								
Multiple antibiotic resistance protein marA (A) - DNA-directed RNA polymerase alpha chain (D)	60 // 657 // 1085	66 // 401 // 632	16 // 121 // 129	17 // 75 // 81	608.8 // 8257.2	589.2 // 4925.6	-105.8 // 1.2 // -1.4 // 0.822	-66.3 // -0.8 // -2.9 // 0.790
**DNA-protein**								
DNA (B) - Multiple antibiotic resistance protein marA (A)	67 // 341 // 415	86 // 657 // 1085	12 // 20 // 20	24 // 121 // 129	742.0 // 4456.5	671.2 // 8257.2	32.3 // -18.0 // -6.3 // 0.026	-105.8 // -0.5 // -2.0 // 0.682
DNA (C) - Multiple antibiotic resistance protein marA (A)	76 // 336 // 407	75 // 657 // 1085	13 // 20 // 20	19 // 121 // 129	678.8 // 4464.9	703.3 // 8257.2	39.2 // -12.6 // -8.9 // 0.269	-105.8 // 0.2 // -1.7 // 0.741
**1YNW**								
**protein-protein**								
Vitamin D3 Receptor (A) - Retinoic acid receptor RXR-alpha (B)	1 // 519 // 761	2 // 360 // 574	1 // 91 // 96	1 // 69 // 73	7.2 // 6961.0	6.5 // 4781.5	-69.4 // -0.1 // -0.0 // 0.339	-61.1 // -0.1 // 0.0 // 0.436
**DNA-protein**								
DNA (C) - Vitamin D3 Receptor (A)	49 // 312 // 370	55 // 519 // 761	8 // 18 // 18	19 // 91 // 96	442.5 // 4206.0	357.9 // 6961.0	37.8 // -9.0 // -5.9 // 0.270	-69.4 // 0.2 // -1.3 // 0.717
DNA (D) - Vitamin D3 Receptor (A)	39 // 309 // 361	44 // 519 // 761	6 // 18 // 18	13 // 91 // 96	427.4 // 4146.8	386.0 // 6961.0	49.3 // -6.7 // -6.2 // 0.443	-69.4 // 4.0 // -1.0 // 0.965
DNA (C) - Retinoic acid receptor RXR-alpha (B)	40 // 312 // 370	38 // 360 // 574	7 // 18 // 18	13 // 69 // 73	374.3 // 4206.0	324.3 // 4781.5	37.8 // -8.3 // -4.8 // 0.231	-61.1 // 3.7 // 0.2 // 0.919
DNA (D) - Retinoic acid receptor RXR-alpha (B)	38 // 309 // 361	40 // 360 // 574	6 // 18 // 18	14 // 69 // 73	384.6 // 4146.8	370.1 // 4781.5	49.3 // -7.5 // -6.1 // 0.367	-61.1 // 4.5 // 0.2 // 0.947
**2AS5**								
**protein-protein**								
Nuclear factor of activated T-cells, cytoplasmic 2 (F) - Forkhead box protein P2 (N)	29 // 430 // 707	40 // 1409 // 2290	11 // 78 // 83	15 // 271 // 287	328.3 // 5602.2	323.5 // 16254.2	-74.5 // 0.4 // -0.4 // 0.674	-250.7 // 0.7 // -0.4 // 0.726
**DNA-protein**								
DNA (A) - Nuclear factor of activated T-cells, cytoplasmic 2 (F)	38 // 359 // 431	47 // 430 // 707	6 // 21 // 21	11 // 78 // 83	408.7 // 4817.1	385.3 // 5602.2	59.3 // -11.0 // -6.3 // 0.150	-74.5 // 0.0 // -0.6 // 0.623
DNA (B) - Nuclear factor of activated T-cells, cytoplasmic 2 (F)	43 // 354 // 424	44 // 430 // 707	8 // 21 // 21	16 // 78 // 83	419.8 // 4761.2	375.0 // 5602.2	48.7 // -9.4 // -5.9 // 0.233	-74.5 // 0.3 // -0.6 // 0.658
DNA (A) - Forkhead box protein P2 (N)	41 // 359 // 431	35 // 1409 // 2290	9 // 21 // 21	13 // 271 // 287	402.7 // 4817.1	412.4 // 16254.2	59.3 // -7.2 // -6.8 // 0.449	-250.7 // 2.8 // -0.4 // 0.938
DNA (B) - Forkhead box protein P2 (N)	56 // 354 // 424	63 // 1409 // 2290	7 // 21 // 21	18 // 271 // 287	617.7 // 4761.2	573.8 // 16254.2	48.7 // -10.8 // -7.7 // 0.272	-250.7 // 0.9 // -0.7 // 0.739
**2BSQ**								
**protein-protein**								
TRAFFICKING PROTEIN A (A) - TRAFFICKING PROTEIN B (E)	135 // 708 // 1122	115 // 420 // 510	42 // 136 // 144	28 // 69 // 69	1236.7 // 8362.4	1421.3 // 6547.9	-131.5 // -9.6 // -6.1 // 0.161	-35.9 // -8.4 // -5.4 // 0.248
**DNA-protein**								
DNA (I) - TRAFFICKING PROTEIN A (A)	12 // 600 // 712	9 // 708 // 1122	3 // 35 // 35	3 // 136 // 144	103.5 // 8146.2	90.7 // 8362.4	108.4 // -2.2 // -2.2 // 0.470	-131.5 // 0.0 // -0.4 // 0.720
DNA (I) - TRAFFICKING PROTEIN B (E)	29 // 600 // 712	20 // 420 // 510	6 // 35 // 35	6 // 69 // 69	187.5 // 8146.2	228.2 // 6547.9	108.4 // -1.8 // -5.2 // 0.797	-35.9 // 0.7 // -0.9 // 0.801
DNA (J) - TRAFFICKING PROTEIN B (E)	27 // 599 // 718	20 // 420 // 510	7 // 35 // 35	9 // 69 // 69	245.9 // 8049.6	209.8 // 6547.9	59.2 // -5.5 // -2.7 // 0.225	-35.9 // 0.1 // -0.9 // 0.724
