# Supplementary material for: Computational Structural Analysis: Multiple Proteins Bound to DNA
Source: PLoS One. 2008 Sep 19;3(9):e3243. doi: 10.1371/journal.pone.0003243 (PMC2532747; doi:10.1371/journal.pone.0003243)
Supplement: Table S2 — The number of observed hydrogen bonds between amino acid and nucleotide moieties in protein-DNA interfaces (group-MultiProteins∶DNA) (0.07 MB DOC) [file pone.0003243.s009.doc]

**Table S2.** The number of observed hydrogen bonds between amino acid and nucleotide moieties in protein-DNA interfaces (group-MultiProteins:DNA)

| Nuc. moiety  Amino acid | A | C | G | T | Deoxyribose | Phosphate | Total |
| --- | --- | --- | --- | --- | --- | --- | --- |
| ARG | **34 (53.3)** | **10 (21.1)** | **154 (95.4)** | **69 (51.3)** | 87 (77.2) | **284 (339.8)** | 638 |
| LYS | **7 (21.3)** | **1 (8.4)** | 40 (38.1) | **7 (20.5)** | 38 (30.8) | **162 (135.8)** | 255 |
| ASN | **41 (11.3)** | 5 (4.4) | **4 (20.2)** | 18 (10.9) | 13 (16.3) | **54 (71.9)** | 135 |
| ASP | 0 (0.8) | **8 (0.3)** | 0 (1.5) | 0 (0.8) | 0 (1.2) | 2 (5.3) | 10 |
| GLN | **16 (7.1)** | 1 (2.8) | **2 (12.7)** | 6 (6.9) | 11 (10.3) | 49 (45.3) | 85 |
| GLU | **15 (2.6)** | **16 (1.1**) | 0 (4.9) | 0 (2.6) | 0 (3.9) | **2 (17.6)** | 33 |
| HIS | 0 (2.2) | 0 (0.9) | **10 (3.9)** | 3 (2.1) | 2 (3.2) | 11 (13.8) | 26 |
| PRO | 0 (0.0) | 0 (0.0) | 0 (0.0) | 0 (0.0) | 0 (0.0) | 0 (0.0) | 0 |
| TYR | 8 (6.5) | 0 (2.6) | **0 (11.7)** | 2 (6.3) | 3 (9.4) | **65 (41.5)** | 78 |
| TRP | 0 (1.2) | 0 (0.5) | 0 (2.3) | 0 (1.2) | 0 (1.8) | **15 (8.0)** | 15 |
| SER | **2 (8.7)** | 4 (3.5) | 10 (16.0) | 4 (8.5) | 12 (12.8) | **74 (56.5)** | 106 |
| THR | 4 (6.7) | 6 (2.6) | **4 (12.0)** | 6 (6.4) | 7 (9.7) | 53 (42.6) | 80 |
| GLY | 3 (3.7) | 1 (1.5) | 10 (6.6) | **10 (3.5)** | 6 (5.3) | **14 (23.4)** | 44 |
| ALA | 0 (1.1) | 0 (0.4) | 2 (2.0) | 0 (1.0) | 1 (1.6) | 10 (7.0) | 13 |
| MET | 0 (0.0) | 0 (0.0) | 0 (0.0) | 0 (0.0) | 0 (0.0) | 0 (0.0) | 0 |
| CYS | 0 (0.8) | 0 (0.3) | 0 (1.5) | 0 (0.8) | 4 (1.2) | 6 (5.3) | 10 |
| PHE | 0 (1.1) | 0 (0.4) | 0 (2.0) | 1 (1.0) | 1 (1.6) | 11 (7.0) | 13 |
| LEU | 0 (1.2) | 0 (0.5) | 0 (2.1) | 1 (1.1) | 1 (1.7) | 12 (7.5) | 14 |
| VAL | 2 (1.3) | 0 (0.5) | 0 (2.4) | 0 (1.3) | 3 (1.9) | 11 (8.5) | 16 |
| ILE | 0 (0.7) | 0 (0.3) | 0 (1.2) | 0 (0.6) | 2 (1.0) | 6 (4.3) | 8 |
| Total | 132 | 52 | 236 | 127 | 191 | 841 | 1579 |

Numbers in parentheses are the expected values assuming random occurrence of interactions. Entries that diverge from the expected distribution (with a probability higher than 0.99) are in bold
