# Supplementary material for: Computational Structural Analysis: Multiple Proteins Bound to DNA
Source: PLoS One. 2008 Sep 19;3(9):e3243. doi: 10.1371/journal.pone.0003243 (PMC2532747; doi:10.1371/journal.pone.0003243)
Supplement: Table S3 — The number of observed hydrogen bonds between amino acid and nucleotide moieties in protein-DNA interfaces (group-SingleSameProtein∶DNA) (0.07 MB DOC) [file pone.0003243.s010.doc]

**Table S3.** The number of observed hydrogen bonds between amino acid and nucleotide moieties in protein-DNA interfaces (group-SingleSameProtein:DNA)

| Nuc. moiety  Amino acid | A | C | G | T | Deoxyribose | Phosphate | Total |
| --- | --- | --- | --- | --- | --- | --- | --- |
| ARG | **4 (12.1)** | 2 (2.7) | **40 (20.5)** | 12 (9.8) | 7 (8.0) | **44 (55.8)** | 109 |
| LYS | 1 (3.7) | 0 (0.8) | 3 (6.2) | 3 (3.0) | 5 (2.4) | 21 (17.0) | 33 |
| ASN | **10 (2.4)** | 0 (0.5) | 0 (4.1) | 5 (2.0) | 1 (1.6) | 6 (11.3) | 22 |
| ASP | 0 (0.0) | 0 (0.0) | 0 (0.0) | 0 (0.0) | 0 (0.0) | 0 (0.0) | 0 |
| GLN | 5 (1.9) | 1 (0.4) | 2 (3.2) | 1 (1.5) | 2 (1.2) | 6 (8.7) | 17 |
| GLU | 2 (0.5) | **3 (0.12)** | 0 (0.9) | 0 (0.5) | 0 (0.4) | 0 (2.6) | 5 |
| HIS | 0 (0.2) | 0 (0.1) | 0 (0.4) | 1 (0.2) | 0 (0.2) | 1 (1.0) | 2 |
| PRO | 0 (0.0) | 0 (0.0) | 0 (0.0) | 0 (0.0) | 0 (0.0) | 0 (0.0) | 0 |
| TYR | 2 (1.6) | 0 (0.4) | 0 (2.8) | 0 (1.3) | 0 (1.1) | 13 (7.7) | 15 |
| TRP | 0 (0.3) | 0 (0.1) | 0 (0.6) | 0 (0.3) | 0 (0.2) | 3 (1.5) | 3 |
| SER | 0 (1.5) | 0 (0.3) | 1 (2.6) | 0 (1.3) | 1 (1.0) | 12 (7.2) | 14 |
| THR | 3 (1.2) | 0 (0.3) | 0 (2.1) | 0 (1.0) | 1 (0.8) | 7 (5.6) | 11 |
| GLY | 0 (0.1) | 0 (0.1) | 0 (0.2) | 0 (0.1) | 0 (0.1) | 1 (0.5) | 1 |
| ALA | 0 (0.0) | 0 (0.0) | 0 (0.0) | 0 (0.0) | 0 (0.0) | 0 (0.0) | 0 |
| MET | 0 (0.0) | 0 (0.0) | 0 (0.0) | 0 (0.0) | 0 (0.0) | 0 (0.0) | 0 |
| CYS | 0 (0.0) | 0 (0.0) | 0 (0.0) | 0 (0.0) | 0 (0.0) | 0 (0.0) | 0 |
| PHE | 0 (0.5) | 0 (0.1) | 0 (0.9) | 0 (0.4) | 0 (0.4) | 5 (2.6) | 5 |
| LEU | 0 (0.8) | 0 (0.2) | 0 (1.3) | 0 (0.6) | 1 (0.5) | 6 (3.6) | 7 |
| VAL | 0 (0.0) | 0 (0.0) | 0 (0.0) | 0 (0.0) | 0 (0.0) | 0 (0.0) | 0 |
| ILE | 0 (0.0) | 0 (0.0) | 0 (0.0) | 0 (0.0) | 0 (0.0) | 0 (0.0) | 0 |
| Total | 27 | 6 | 46 | 22 | 18 | 125 | 244 |

Numbers in parentheses are the expected values assuming random occurrence of interactions. Entries that diverge from the expected distribution (with probability higher than 0.99) are in bold
