# Supplementary material for: Computational Structural Analysis: Multiple Proteins Bound to DNA
Source: PLoS One. 2008 Sep 19;3(9):e3243. doi: 10.1371/journal.pone.0003243 (PMC2532747; doi:10.1371/journal.pone.0003243)
Supplement: Table S4 — The number of observed hydrogen bonds between amino acid and nucleotide moieties in protein-DNA interfaces (group-SubSetMultiProteins∶DNA). (0.06 MB DOC) [file pone.0003243.s011.doc]

**Table S4.** The number of observed hydrogen bonds between amino acid and nucleotide moieties in protein-DNA interfaces (group-SubSetMultiProteins:DNA).

| Nuc. moiety  Amino acid | A | C | G | T | Deoxyribose | Phosphate | Total |
| --- | --- | --- | --- | --- | --- | --- | --- |
| ARG | 25 (29.2) | 9 (13.4) | **83 (52.8)** | **34 (23.2)** | 22 (24.0) | **130 (160.4)** | 303 |
| LYS | **2 (11.2)** | 2 (5.2) | 22 (20.3) | 4 (8.9) | 15 (0.2) | 73 (61.6) | 118 |
| ASN | **29 (7.1)** | 4 (3.3) | 4 (12.9) | 11 (5.7) | 1 (5.8) | **26 (39.2)** | 75 |
| ASP | 0 (0.5) | 5 (0.2) | 0 (0.9) | 0 (0.4) | 0 (0.4) | 0 (2.6) | 5 |
| GLN | 8 (4.1) | 1 (1.9) | 0 (7.4) | 2 (3.2) | 8 (3.4) | 24 (22.5) | 43 |
| GLU | 1 (0.9) | **7 (0.4)** | 0 (1.5) | 0 (0.7) | 0 (0.7) | 1 (4.7) | 9 |
| HIS | 0 (1.1) | 0 (0.5) | 2 (1.9) | 2 (0.8) | 1 (0.9) | 6 (5.7) | 11 |
| PRO | 0 (0.0) | 0 (0.0) | 0 (0.0) | 0 (0.0) | 0 (0.0) | 0 (0.0) | 0 |
| TYR | 2 (3.6) | 0 (1.7) | 0 (6.5) | 0 (2.9) | 1 (3.0) | **35 (19.8)** | 38 |
| TRP | 0 (1.0) | 0 (0.4) | 0 (1.7) | 0 (0.8) | 0 (0.8) | 10 (5.2) | 10 |
| SER | 3 (4.3) | 1 (2.0) | 9 (7.7) | 2 (3.4) | 1 (3.5) | 29 (23.5) | 45 |
| THR | 1 (3.0) | 2 (1.4) | 2 (5.3) | 1 (2.3) | 3 (2.4) | 22 (16.2) | 31 |
| GLY | 1 (1.8) | 1 (0.8) | 7 (3.3) | 1 (1.4) | 0 (1.5) | 9 (9.9) | 19 |
| ALA | 0 (0.8) | 0 (0.3) | 0 (1.4) | 0 (0.6) | 1 (0.6) | 7 (4.2) | 8 |
| MET | 0 (0.0) | 0 (0.0) | 0 (0.0) | 0 (0.0) | 0 (0.0) | 0 (0.0) | 0 |
| CYS | 0 (0.8) | 0 (0.4) | 0 (1.4) | 0 (0.6) | 3 (0.6) | 5 (4.2) | 8 |
| PHE | 0 (0.7) | 1 (0.3) | 0 (1.2) | 0 (0.5) | 1 (0.5) | 5 (3.7) | 7 |
| LEU | 0 (0.5) | 0 (0.2) | 0 (0.9) | 0 (0.4) | 0 (0.4) | 5 (2.6) | 5 |
| VAL | 0 (0.6) | 0 (0.3) | 1 (1.0) | 0 (0.4) | 0 (0.5) | 5 (3.1) | 6 |
| ILE | 0 (0.5) | 0 (0.2) | 0 (0.9) | 0 (0.4) | 2 (0.4) | 3 (2.6) | 5 |
| Total | 72 | 33 | 130 | 57 | 59 | 395 | 746 |

Numbers in parentheses are the expected values assuming random occurrence of interactions. Entries that diverge from the expected distribution (with probability higher than 0.99) are in bold
