# Supplementary material for: Computational Structural Analysis: Multiple Proteins Bound to DNA
Source: PLoS One. 2008 Sep 19;3(9):e3243. doi: 10.1371/journal.pone.0003243 (PMC2532747; doi:10.1371/journal.pone.0003243)
Supplement: Table S5 — Number of observed van der Waals contacts between amino acid and nucleotide moieties in protein-DNA interfaces (group-MultiProteins∶DNA). (0.06 MB DOC) [file pone.0003243.s012.doc]

**Table S5.** Number of observed van der Waals contacts between amino acid and nucleotide moieties in protein-DNA interfaces (group-MultiProteins:DNA).

| Nuc. moiety  Amino acid | A | C | G | T | Deoxyribose | Phosphate | Total |
| --- | --- | --- | --- | --- | --- | --- | --- |
| ARG | 223 (246.0) | 151 (146.5) | **489 (242.7)** | **375 (327.4)** | 929 (947.8) | **724 (980.5)** | 2891 |
| LYS | **33 (102.2)** | **14 (60.8)** | **52 (100.8)** | **64 (136.0)** | **291 (393.7)** | **747 (407.3)** | 1201 |
| ASN | **133 (60.9)** | **21 (26.3)** | 67 (60.1) | 63 (81.1) | 258 (234.7) | **174 (242.8)** | 716 |
| ASP | 0 (3.7) | **13 (2.2)** | 1 (3.7) | **0 (5.0)** | 8 (14.4) | 22 (14.9) | 44 |
| GLN | 67 (55.1) | 45 (32.8) | **12 (54.3)** | **96 (73.3)** | 219 (212.1) | 208 (219.4) | 647 |
| GLU | 12 (17.4) | **61 (10.4)** | **3 (17.2)** | **48 (23.2)** | **41 (67.2)** | **40 (69.5)** | 205 |
| HIS | **13 (27.4)** | 22 (16.3) | **52 (27.0)** | **52 (36.5)** | 94 (105.6) | 89 (109.2) | 322 |
| PRO | 29 (18.7) | 5 (11.1) | **2 (18.5)** | 21 (25.0) | 82 (72.1) | 81 (74.6) | 220 |
| TYR | 30 (40.7) | **11 (24.2)** | **4 (40.1)** | 46 (54.1) | 167 (156.7) | **220 (162.1)** | 478 |
| TRP | **5 (14.7)** | **37 (8.8)** | **3 (14.5)** | **6 (19.6)** | **29 (56.7)** | **93 (58.7)** | 173 |
| SER | **17 (51.4)** | 18 (30.6) | **69 (50.7)** | 69 (68.4) | 217 (198.0) | 214 (204.8) | 604 |
| THR | **19 (41.2)** | 36 (24.5) | **10 (40.6)** | 63 (54.8) | 166 (158.7) | 190 (164.1) | 484 |
| GLY | 24 (31.8) | 22 (18.9) | 35 (31.4) | 34 (43.4) | **178 (122.6)** | **81 (126.8)** | 374 |
| ALA | 6 (14.2) | 8 (8.5) | 11 (14.0) | 26 (18.9) | 55 (54.7) | 61 (56.6) | 167 |
| MET | **75 (13.3)** | 6 (7.9) | **1 (13.1)** | **1 (17.7)** | 51 (51.1) | **22 (52.9)** | 156 |
| CYS | 0 (3.9) | 1 (2.3) | 0 (3.9) | 2 (5.2) | 18 (15.1) | **25 (15.6)** | 46 |
| PHE | 47 (34.3) | **1 (20.4)** | **6 (33.8)** | 46 (45.6) | **203 (132.1)** | **100 (136.7)** | 403 |
| LEU | 11 (12.6) | **17 (7.5)** | **0 (12.4)** | 17 (16.8) | 60 (48.5) | 43 (50.2) | 148 |
| VAL | **70 (24.8)** | **2 (14.7)** | **0 (24.4)** | **56 (33.0)** | **57 (95.4)** | 106 (98.7) | 291 |
| ILE | 14 (13.6) | 2 (8.1) | **0 (13.4)** | 17 (18.1) | 67 (52.5) | 60 (54.3) | 160 |
| Total | 828 | 493 | 817 | 1102 | 3190 | 3300 | 9730 |

Numbers in parentheses are the expected values assuming random occurrence of interactions. Entries that diverge from the expected distribution (with a probability higher than 0.99) are in bold.
