# Supplementary material for: Computational Structural Analysis: Multiple Proteins Bound to DNA
Source: PLoS One. 2008 Sep 19;3(9):e3243. doi: 10.1371/journal.pone.0003243 (PMC2532747; doi:10.1371/journal.pone.0003243)
Supplement: Table S6 — Number of observed van der Waals contacts between amino acid and nucleotide moieties in protein-DNA interfaces (group-SingleSameProtein∶DNA). (0.07 MB DOC) [file pone.0003243.s013.doc]

**Table S6.** Number of observed van der Waals contacts between amino acid and nucleotide moieties in protein-DNA interfaces (group-SingleSameProtein:DNA).

| Nuc. moiety  Amino acid | A | C | G | T | Deoxyribose | Phosphate | Total |
| --- | --- | --- | --- | --- | --- | --- | --- |
| ARG | 25 (28.2) | **22 (11.6)** | **33 (17.8)** | 35 (33.0) | **28 (40.7)** | 34 (45.7) | 177 |
| LYS | 8 (13.5) | 3 (5.6) | 10 (8.6) | 9 (15.8) | 25 (19.5) | 30 (22.0) | 85 |
| ASN | **12 (5.4)** | 2 (2.2) | 2 (3.4) | 12 (6.3) | 4 (7.8) | **2 (8.8)** | 34 |
| ASP | 0 (0.2) | 0 (0.1) | 0 (0.1) | 1 (0.2) | 0 (0.2) | 0 (0.3) | 1 |
| GLN | 8 (4.5) | 1 (1.8) | 3 (2.8) | 1 (5.2) | 11 (6.4) | 4 (7.2) | 28 |
| GLU | 2 (2.4) | **5 (1.0)** | 1 (1.5) | 5 (2.8) | 0 (3.5) | 2 (3.9) | 15 |
| HIS | 3 (1.6) | 0 (0.7) | 1 (1.0) | 2 (1.9) | 1 (2.3) | 3 (2.6) | 10 |
| PRO | 1 (1.3) | 0 (0.5) | 0 (0.8) | 1 (1.5) | 4 (1.8) | 2 (2.1) | 8 |
| TYR | 2 (4.2) | 1 (1.7) | 0 (2.6) | 4 (4.8) | 3 (6.0) | **16 (6.7)** | 26 |
| TRP | 0 (1.0) | 0 (0.4) | 1 (0.6) | 1 (1.1) | 0 (1.4) | 4 (1.6) | 6 |
| SER | 0 (4.0) | 1 (1.6) | 4 (2.5) | 6 (4.7) | 6 (5.7) | 8 (6.5) | 25 |
| THR | 1 (4.5) | 0 (1.8) | 2 (2.8) | 7 (5.2) | 11 (6.4) | 7 (7.2) | 28 |
| GLY | 3 (3.0) | 1 (1.2) | 1 (1.9) | 1 (3.5) | 8 (4.4) | 5 (4.9) | 19 |
| ALA | 2 (2.2) | 0 (0.9) | 0 (1.4) | 4 (2.6) | 4 (3.2) | 4 (3.6) | 14 |
| MET | 0 (1.4) | 0 (0.6) | 0 (0.9) | 1 (1.7) | 2 (2.1) | 6 (2.3) | 9 |
| CYS | 0 (0.2) | 0 (0.1) | 0 (0.1) | 0 (0.2) | 0 (0.2) | 1 (0.3) | 1 |
| PHE | 6 (5.1) | 0 (2.1) | 0 (3.2) | 8 (6.0) | 10 (7.4) | 8 (8.3) | 32 |
| LEU | 3 (3.8) | 0 (1.6) | 1 (2.4) | 6 (4.5) | 6 (5.5) | 8 (6.2) | 24 |
| VAL | **15 (5.4)** | 2 (2.2) | 1 (3.4) | 6 (6.3) | 7 (7.8) | 3 (8.8) | 34 |
| ILE | 4 (3.2) | 1 (1.3) | 0 (2.0) | 1 (3.7) | 7 (4.6) | 7 (5.2) | 20 |
| Total | 95 | 39 | 60 | 111 | 137 | 154 | 596 |

Numbers in parentheses are the expected values assuming random occurrence of interactions. Entries that diverge from the expected distribution (with probability higher than 0.99) are in bold.
