# Supplementary material for: Computational Structural Analysis: Multiple Proteins Bound to DNA
Source: PLoS One. 2008 Sep 19;3(9):e3243. doi: 10.1371/journal.pone.0003243 (PMC2532747; doi:10.1371/journal.pone.0003243)
Supplement: Table S7 — Number of observed van der Waals contacts between amino acid and nucleotide moieties in protein-DNA interfaces (group-SubSetMultiProteins∶DNA). (0.06 MB DOC) [file pone.0003243.s014.doc]

**Table S7.** Number of observed van der Waals contacts between amino acid and nucleotide moieties in protein-DNA interfaces (group-SubSetMultiProteins:DNA).

| Nuc. moiety  Amino acid | A | C | G | T | Deoxyribose | Phosphate | Total |
| --- | --- | --- | --- | --- | --- | --- | --- |
| ARG | 34 (32.3) | 17 (15.6) | **58 (32.3)** | 62 (49.8) | 84 (93.9) | **124 (155.2)** | 379 |
| LYS | **6 (18.3)** | 3 (8.8) | 19 (18.3) | 10 (28.2) | 46 (53.3) | **131 (88.0)** | 215 |
| ASN | **27 (9.9)** | 6 (4.8) | 10 (0.9) | 18 (15.2) | 24 (28.8) | **31 (47.5)** | 116 |
| ASP | 0 (0.5) | 4 (0.2) | 1 (0.5) | 0 (0.8) | 0 (1.5) | 1 (2.5) | 6 |
| GLN | 7 (5.8) | 3 (2.8) | 3 (5.8) | 8 (8.9) | 20 (16.9) | 27 (27.8) | 68 |
| GLU | 2 (2.3) | **9 (1.1)** | 0 (2.3) | 6 (3.5) | 5 (6.7) | 5 (11.1) | 27 |
| HIS | 1 (2.5) | 2 (1.2) | 2 (2.6) | 6 (3.9) | 10 (7.4) | 9 (12.3) | 30 |
| PRO | 3 (2.4) | 0 (1.1) | 0 (2.4) | 0 (3.7) | 9 (6.9) | 16 (11.5) | 28 |
| TYR | 2 (5.5) | 0 (2.6) | 0 (5.4) | 4 (8.4) | 16 (15.6) | **42 (26.2)** | 64 |
| TRP | 1 (2.2) | 2 (1.1) | 0 (2.2) | 1 (3.4) | 3 (6.4) | 19 (10.6) | 26 |
| SER | 1 (6.4) | 3 (3.1) | 9 (6.4) | 12 (0.8) | 18 (18.6) | 32 (30.1) | 75 |
| THR | 1 (4.9) | 2 (2.4) | 4 (4.9) | 8 (7.6) | 14 (14.4) | 29 (23.7) | 58 |
| GLY | 1 (5.0) | 4 (2.4) | 9 (5.0) | 3 (7.7) | 25 (14.6) | 17 (24.1) | 59 |
| ALA | 2 (2.5) | 0 (1.2) | 0 (2.5) | 9 (3.9) | 6 (7.4) | 13 (12.3) | 30 |
| MET | 0 (0.8) | 1 (0.4) | 1 (0.8) | 0 (1.2) | 4 (2.2) | 3 (3.7) | 9 |
| CYS | 0 (0.8) | 0 (0.4) | 0 (0.8) | 1 (1.2) | 3 (2.2) | 5 (3.7) | 9 |
| PHE | 6 (3.6) | 0 (1.7) | 0 (3.6) | 6 (5.5) | 12 (10.4) | 18 (17.2) | 42 |
| LEU | 4 (3.0) | 0 (1.4) | 0 (3.0) | 6 (4.6) | 15 (8.7) | 10 (14.3) | 35 |
| VAL | **15 (5.0)** | 0 (2.4) | 0 (5.02) | 17 (7.7) | 9 (14.6) | 18 (24.1) | 59 |
| ILE | 3 (2.4) | 0 (1.1) | 0 (2.4) | 2 (3.7) | 15 (6.9) | 8 (11.5) | 28 |
| Total | 116 | 56 | 116 | 179 | 338 | 558 | 1363 |

Numbers in parentheses are the expected values assuming random occurrence of interactions. Entries that diverge from the expected distribution (with probability higher than 0.99) are in bold.
