# Supplementary material for: Computational Structural Analysis: Multiple Proteins Bound to DNA
Source: PLoS One. 2008 Sep 19;3(9):e3243. doi: 10.1371/journal.pone.0003243 (PMC2532747; doi:10.1371/journal.pone.0003243)
Supplement: Table S8 — The number of water-mediated contacts in protein-protein and protein-DNA intrerfaces of selected complexes in group-MultipleProteins∶DNA (0.04 MB PDF) [file pone.0003243.s015.pdf]

Table S8. The number of water-mediated contacts in protein-protein and protein-DNA intrerfaces of selected complexes in group-MultipleProteins:DNA

	protein-protein	protein-DNA
**1awc**	4	5
		0
**1b8i**	1	14
		10
**1b72**	1	13
		15
**1d3u**	9	7
		9
**1dsz**	3	20
		26
**1h8a**		
		0
**1k78**	3	19
		5
		16
**1LE8**	5	6
		12
**1MNM**	2	4
		3
**1nh2**	14	22
		6
**1nkp**	8	17
		20
**1nlw**	4	3
		15
**1puf**	6	23
		16
**1R0O**	5	15
		16
**1RIO**	6	10
		4
**1x9m**	4	22
		0
**2nll**	3	5
		24
