# Supplementary material for: Computational Structural Analysis: Multiple Proteins Bound to DNA
Source: PLoS One. 2008 Sep 19;3(9):e3243. doi: 10.1371/journal.pone.0003243 (PMC2532747; doi:10.1371/journal.pone.0003243)
Supplement: Table S9 — Detailed list of rmsd values calculated from fitting each DNA structure in the complexes from group-MultiProteins∶DNA to a corresponding canonical A-DNA and B-DNA. (0.04 MB PDF) [file pone.0003243.s016.pdf]

Table S9. Detailed list of rmsd values calculated from fitting each DNA structure in the complexes from group-MultiProteins:DNA to a corresponding canonical A-DNA and B-DNA.

	A-DNA	B-DNA
1A02	10.245	4.923
1AKH	10.006	6.029
1AWC	7.788	3.091
1B72	7.639	2.497
1B8I	9.744	4.553
1CF7	6.997	1.476
1CQT	6.765	1.656
1D3U	8.382	9.838
1DSZ	6.43	1.428
1FOS	8.018	2.396
1GT0	9.702	5.438
1H8A	9.058	3.138
1H9D	4.312	1.641
1HBX	9.72	7.083
1HJB	8.611	4.1
1IO4	8.458	3.263
1JEY	19.454	18.705
1JFI	5.144	8.617
1K6O	7.746	5.689
1K78	10.901	4.786
1LB2	14.308	9.81
1LE5	7.574	2.751
1LE8	8.15	4.817
1MDM	9.174	2.756
1MNM	7.139	10.453
1N6J	-	-
1NGM	7.011	7.32
1NH2	5.011	7.35
1NKP	7.41	3.029
1NLW	5.501	2.51
1O4X	6.902	4.419
1OUZ	8.659	4.138
1PUF	8.064	2.874
1R0O	8.844	2.862
1RIO	8.215	3.945
1RZR	5.728	3.582
1T2K	9.681	3.923
1TQE	9.11	3.7
1X9M	4.002	5.453
1XS9	6.387	4.435
1YNW	8.225	2.434
2AS5	9.968	5.25
2BSQ	8.604	5.667
2F8X	6.263	1.957
2FO1	9.834	4.532
2NLL	6.81	1.501
