# Supplementary material for: Computational Structural Analysis: Multiple Proteins Bound to DNA
Source: PLoS One. 2008 Sep 19;3(9):e3243. doi: 10.1371/journal.pone.0003243 (PMC2532747; doi:10.1371/journal.pone.0003243)
Supplement: Table S10 — Detailed list of rmsd values calculated from fitting each DNA structure in the complexes from group-SingleProtein∶DNA to a corresponding canonical A-DNA and B-DNA. (0.04 MB PDF) [file pone.0003243.s017.pdf]

Table S10. Detailed list of rmsd values calculated from fitting each DNA structure in the complexes from group-SingleProtein:DNA to a corresponding canonical A-DNA and B-DNA.

	A-DNA	B-DNA
1A0A	5.465	2.881
1A3Q	5.366	1.5
1AM9	5.525	2.678
1B01	-	-
1B3T	5.802	3.561
1BDT	8.413	4.159
1BG1	5.306	2.272
1BL0	10.161	5.993
1BPY	3.841	2.156
1C8C	2.5	3.239
1CEZ	-	-
1CKT	4.351	5.523
1CL8	5.596	2.433
1CW0	7.078	4.169
1D02	6.082	1.876
1DC1	9.122	4.973
1DDN	9.83	5.961
1DEW	6.42	3.671
1DFM	9.794	4.652
1DH3	5.876	2.179
1DIZ	3.563	7.209
1DMU	-	-
1DP7	4.384	2.094
1E3O	4.654	1.637
1ECR	7.015	3.695
1EFA	5.836	4.418
1EGW	-	-
1ESG	3.652	1.194
1EWN	4.652	4.759
1EWQ	9.208	6.517
1EYG	-	-
1F44	7.383	3.176
1F4K	7.015	1.813
1FOK	8.853	3.434
1FZP	4.941	2.877
1G38	6.497	3.933
1G9Z	-	-
1GDT	-	-
1HLV	7.64	4.031
1HWT	8	2.221
1I3J	7.548	2.791
1I6J	2.342	0.861
1I7D	2.54	3.581
1IAW	6.654	4.571
1IC8	7.096	2.085
1IGN	8.579	3.491
1J1V	5.147	2.319
1JB7	4.911	5.212
1JE8	5.94	4.981
1JJ4	6.81	3.2
1JMC	6.433	6.439
1JT0	8.365	3.485
1JX4	4.845	2.45
1K3X	-	-
1K4T	3.359	1.132

**1KC6**	5.405	4.584
**1KDH**	1.997	2.421
**1KU7**	5.923	2.134
**1L3L**	5.59	3.259
**1L3S**	2.497	1.696
**1LLM**	6.34	2.48
**1LMB**	9.047	3.345
**1LQ1**	8.164	3.627
**1LRR**	6.538	0.986
**1LWY**	4.937	5.404
**1M5R**	8.089	4.652
**1MHD**	6.036	1.616
**1MJO**	7.798	3.099
**1MNN**	7.699	2.266
**1MUS**	6.482	5.284
**1MW8**	2.983	3.418
**1MWI**	4.779	2.959
**1ODH**	4.058	3.095
**1OE4**	5.972	3.046
**1ORN**	4.11	4.701
**1OUP**	2.554	2.272
**1P4E**	3.432	2.429
**1P71**	7.687	7.944
**1P7H**	4.598	1.304
**1PV4**	-	-
**1QNA**	3.652	7.343
**1QPZ**	5.78	4.325
**1QRV**	2.803	6.346
**1QUM**	1.786	1.282
**1REP**	6.612	3.136
**1SKN**	9.587	4.847
**1TC3**	6.24	6.353
**1TRO**	7.618	2.656
**1TUP**	8.177	3.05
**1UBD**	6.543	2.103
**1VAS**	7.336	5.384
**1ZME**	7.214	4.083
**2BOP**	5.853	3.343
**2CGP**	3.391	1.94
**2DRP**	5.422	2.702
**2HDD**	8.167	2.817
**2IRF**	5.859	2.274
**2PJR**	3.109	3.34
**3HTS**	4.247	1.023
**3PVI**	7.858	4.038
**6CRO**	-	-
**6MHT**	6.449	2.995
