# Supplementary material for: Computational Structural Analysis: Multiple Proteins Bound to DNA
Source: PLoS One. 2008 Sep 19;3(9):e3243. doi: 10.1371/journal.pone.0003243 (PMC2532747; doi:10.1371/journal.pone.0003243)
Supplement: Table S11 — Detailed list of rmsd values calculated from fitting each DNA structure in the complexes from group-SingleSameProtein∶DNA to a corresponding canonical A-DNA and B-DNA. (0.03 MB PDF) [file pone.0003243.s018.pdf]

Table S11. Detailed list of rmsd values calculated from fitting each DNA structure in the complexes from group-SingleSameProtein:DNA to a corresponding canonical A-DNA and B-DNA.

	A-DNA	B-DNA
**1A66**	6.477	1.303
**2H7H**	5.817	2.262
**1LFU**	6.987	1.612
**1TGH**	3.095	7.497
**1GU4**	7.043	2.54
**1BC8**	5.258	2.284
**1Y05**	4.094	3.397
**2RAM**	7.038	2.031
**1K61**	8.158	2.59
**1YTB**	-	-
**1TTU**	9.689	4.363
**1P7H**	10.462	5.235
**1KB2**	6.535	1.519
**1U8B**	2.234	0.958
**1KU7**	5.923	2.134
**1C7U**	10.129	3.532
**9ANT**	7.636	2.632
