# Supplementary material for: Computational Structural Analysis: Multiple Proteins Bound to DNA
Source: PLoS One. 2008 Sep 19;3(9):e3243. doi: 10.1371/journal.pone.0003243 (PMC2532747; doi:10.1371/journal.pone.0003243)
Supplement: Table S12 — Detailed list of rmsd values calculated from fitting each DNA structure in the complexes from group-SubSetMutliProteins∶DNA to a corresponding canonical A-DNA and B-DNA. (0.04 MB PDF) [file pone.0003243.s019.pdf]

Table S12. Detailed list of rmsd values calculated from fitting each DNA structure in the complexes from group-SubSetMutliProteins:DNA to a corresponding canonical A-DNA and B-DNA.

	A-DNA	B-DNA
**1A02**	10.245	4.923
**1B72**	7.639	2.497
**1B8I**	9.744	4.553
**1D3U**	8.382	9.838
**1H8A**	9.058	3.138
**1HJB**	8.611	4.1
**1IO4**	8.458	3.263
**1JFI**	5.144	8.617
**1K6O**	7.746	5.689
**1K78**	10.901	4.786
**1LE5**	7.574	2.751
**1MNM**	7.139	10.453
**1PUF**	8.064	2.874
**1RIO**	8.215	3.945
**1T2K**	9.681	3.923
**1XS9**	6.387	4.435
**1YNW**	8.225	2.434
**2AS5**	9.968	5.25
**2FO1**	9.834	4.532
