# Supplementary material for: Computational Structural Analysis: Multiple Proteins Bound to DNA
Source: PLoS One. 2008 Sep 19;3(9):e3243. doi: 10.1371/journal.pone.0003243 (PMC2532747; doi:10.1371/journal.pone.0003243)
Supplement: Table S13 — Average rmsd values calculated from fitting each DNA structure in the complexes from group -SubSetMultiProteins∶DNA and -SingleSameProtein∶DNA to a corresponding canonical A-DNA and B-DNA. (0.03 MB DOC) [file pone.0003243.s020.doc]

**Table S13. Average rmsd values calculated from fitting each DNA structure in the complexes from group –SubSetMultiProteins:DNA and –SingleSameProtein:DNA to a corresponding canonical A-DNA and B-DNA.**

| Dataset of complexes | Average rmsd (± SE)  from A-DNA | Average rmsd (± SE) from B-DNA |
| --- | --- | --- |
| Group-SubSetMultiProteins:DNA | 8.47±0.3 | 4.84±0.5 |
| Group-SingleSameProtein:DNA | 6.66±0.6 (p=0.003) | 2.87±0.4 (p=0.004) |

p-values are calculated in comparison with Group-SubSetMultiProteins:DNA and obtained using the one-tailed Student’s t-test
