# Supplementary material for: Computational Structural Analysis: Multiple Proteins Bound to DNA
Source: PLoS One. 2008 Sep 19;3(9):e3243. doi: 10.1371/journal.pone.0003243 (PMC2532747; doi:10.1371/journal.pone.0003243)
Supplement: Table S14 — Detailed list of energies for each complex in group-MultiProteins∶DNA (0.04 MB PDF) [file pone.0003243.s021.pdf]

Table S14. Detailed list of energies for each complex in group-MultiProteins:DNA

	deltaG-int (kcal/mol)	deltaG-diss (kcal/mol)	deltaG-int (kJ/mol)	deltaG-diss (kJ/mol)
**1A02**	-48.1	20.5	-201.38508	85.8294
**1AKH**	-27.5	13.6	-115.137	56.94048
**1AWC**	-42.8	4.8	-179.19504	20.09664
**1B72**	-33.1	12.8	-138.58308	53.59104
**1B8I**	-27.4	5.3	-114.71832	22.19004
**1CF7**	-37.6	14.2	-157.42368	59.45256
**1CQT**	-41.2	11.5	-172.49616	48.1482
**1D3U**	-46.6	13.8	-195.10488	57.77784
**1DSZ**	-21.8	4.2	-91.27224	17.58456
**1FOS**	-50.6	15.6	-211.85208	65.31408
**1GT0**	-33.7	4.9	-141.09516	20.51532
**1H8A**	-71	17.1	-297.2628	71.59428
**1H9D**	-22	3	-92.1096	12.5604
**1HBX**	-117.6	29.7	-492.36768	124.34796
**1HJB**	-59.2	3.4	-247.85856	14.23512
**1IO4**	-68.1	3	-285.12108	12.5604
**1JEY**	-162.7	30.2	-681.19236	126.44136
**1JFI**	-80.5	7.3	-337.0374	30.56364
**1K6O**	-88.3	12.6	-369.69444	52.75368
**1K78**	-67.1	7.8	-280.93428	32.65704
**1LB2**	-40.5	3	-169.5654	12.5604
**1LE5**	-39.4	14	-164.95992	58.6152
**1LE8**	-37.9	12.8	-158.67972	53.59104
**1MDM**	-49.8	7.1	-208.50264	29.72628
**1MNM**	-103.4	1.3	-432.91512	5.44284
**1N6J**	-76.3	7.1	-319.45284	29.72628
**1NGM**	-36.6	9.9	-153.23688	41.44932
**1NH2**	-63.4	6.3	-265.44312	26.37684
**1NKP**	-73.3	34.1	-306.89244	142.76988
**1NLW**	-65.4	28.7	-273.81672	120.16116
**1O4X**				
**1OUZ**	-81.6	37.6	-341.64288	157.42368
**1PUF**	-34.3	10.5	-143.60724	43.9614
**1R0O**	-28.8	3	-120.57984	12.5604
**1RIO**	-66.6	4.3	-278.84088	18.00324
**1RZR**	-75.8	32.2	-317.35944	134.81496
**1T2K**	-92.5	7.7	-387.279	32.23836
**1TQE**	-82.7	7	-346.24836	29.3076
**1X9M**	-35.2	8.4	-147.37536	35.16912
**1XS9**				
**1YNW**	-32.7	4	-136.90836	16.7472
**2AS5**	-43.9	16.9	-183.80052	70.75692
**2BSQ**				
**2F8X**	-47.2	9.3	-197.61696	38.93724
**2FO1**	-34.7	7	-145.28196	29.3076
**2NLL**	-20.6	10.2	-86.24808	42.70536
