# Supplementary material for: Computational Structural Analysis: Multiple Proteins Bound to DNA
Source: PLoS One. 2008 Sep 19;3(9):e3243. doi: 10.1371/journal.pone.0003243 (PMC2532747; doi:10.1371/journal.pone.0003243)
Supplement: Table S15 — Detailed list of energies for each complex in group-SubMultiProteins∶DNA (0.04 MB PDF) [file pone.0003243.s022.pdf]

Table S15. Detailed list of energies for each complex in group-SubMultiProteins:DNA

	deltaG-int (kcal/mol)	deltaG-diss (kcal/mol)	deltaG-int (kJ/mol)	deltaG-diss (kJ/mol)
**1A02-F**	-22	6.9	-92.1096	28.88892
**1A02-J**	-18.2	4.1	-76.19976	17.16588
**1A02-N**	-15	2.8	-62.802	11.72304
**1AKH-A**	-14	10.6	-58.6152	44.38008
**1AKH-B**	-5.9	4.6	-24.70212	19.25928
**1B72-A**	-18.5	7.1	-77.4558	29.72628
**1B72-B**	-15.7	5.5	-65.73276	23.0274
**1B8I-A**	-16.9	9.1	-70.75692	38.09988
**1B8I-B**	-9.7	-0.5	-40.61196	-2.0934
**1CF7-A**	-17.9	3.9	-74.94372	16.32852
**1CF7-B**	-17.2	0.8	-72.01296	3.34944
**1CQT-B**	-25.8	25.5	-108.01944	106.7634
**1CQT-J**	-16.2	3	-67.82616	12.5604
**1D3U-A**	-23.8	5.3	-99.64584	22.19004
**1D3U-B**	-31.3	17.7	-131.04684	74.10636
**1DSZ-A**	-17.9	3.7	-74.94372	15.49116
**1DSZ-B**	-14.7	3.7	-61.54596	15.49116
**1FOS-G**	-21	3.4	-87.9228	14.23512
**1FOS-H**	-19.3	2.7	-80.80524	11.30436
**1GT0-C**	-27.9	19.4	-116.81172	81.22392
**1GT0-D**	-17.7	5.2	-74.10636	21.77136
**1H8A-A,B**	-52.2	19.3	-218.55096	80.80524
**1H8A-C**	-33.7	17.7	-141.09516	74.10636
**1HBX-A,B**	-89	44.8	-372.6252	187.56864
**1HBX-G**	-28.7	12.9	-120.16116	54.00972
**1HJB-D,E**	-52.8	18.9	-221.06304	79.13052
**1HJB-F**	-20.2	4.1	-84.57336	17.16588
**1IO4-A,B**	-51.3	14.8	-214.78284	61.96464
**1IO4-C**	-27.5	4.8	-115.137	20.09664
**1JEY-A**	-22.4	1.6	-93.78432	6.69888
**1JEY-B**	-31.5	15.9	-131.8842	66.57012
**1JFI-A**				
**1JFI-B**	-16.1	-1.7	-67.40748	-7.11756
**1JFI-C**	-21.1	4.9	-88.34148	20.51532
**1K6O-A**	-22.2	10.8	-92.94696	45.21744
**1K6O-B,C**	-72.2	26.3	-302.28696	110.11284
**1K78-A,I**	-53.4	6.7	-223.57512	28.05156
**1K78-B**	-30.9	7.2	-129.37212	30.14496
**1LB2-A**	-28.3	10.3	-118.48644	43.12404
**1LB2-B,E**	-20.4	2.4	-85.41072	10.04832
**1LE5-A**	-17.4	6.4	-72.85032	26.79552
**1LE5-B**	-24	10.6	-100.4832	44.38008
**1LE8-A**	-20.8	8.2	-87.08544	34.33176
**1LE8-B**	-18.5	4.4	-77.4558	18.42192
**1MDM-A**	-38.6	27.1	-161.61048	113.46228
**1MDM-B**	-28.1	5.9	-117.64908	24.70212
**1MNM-A,B**	-76.4	28.6	-319.87152	119.74248
**1MNM-C,D**	-22.4	7.2	-93.78432	30.14496
**1N6J-A,B**	-65.3	13.4	-273.39804	56.10312
**1N6J-G**				
**1NGM-A**	-25.5	8.6	-106.7634	36.00648
**1NGM-B**				
**1NH2-A,B**	1.6	2.7	6.69888	11.30436
**1NH2-B,C**	-17.2	21.1	-72.01296	88.34148
**1NKP-A**	-25.4	11.2	-106.34472	46.89216
**1NKP-B**	-27.9	12.5	-116.81172	52.335
**1NLW-A**	-27.9	12.5	-116.81172	52.335
**1NLW-B**	-26.2	10.4	-109.69416	43.54272
**1O4X-A**				
**1O4X-B**				
**1OUZ-A**	-31.2	15.7	-130.62816	65.73276
**1OUZ-B**	-29.2	10.7	-122.25456	44.79876
**1PUF-A**	-22.8	17.5	-95.45904	73.269
**1PUF-B**	-16.2	4.7	-67.82616	19.67796
**1R0O-A**	-23.2	8.8	-97.13376	36.84384
**1R0O-B**	-18.4	2.6	-77.03712	10.88568
**1RIO-A,B**	-75.3	53.9	-315.26604	225.66852
**1RIO-H**	-35	5.1	-146.538	21.35268
**1RZR-A,D**	-114.4	32.2	-478.96992	134.81496

**1RZR-S,Y**				
**1T2K-A,B**	-58.3	11	-244.09044	46.0548
**1T2K-C,D**	-55.5	8.8	-232.3674	36.84384
**1TQE-S,R**	-69.4	18.1	-290.56392	75.78108
**1TQE-Y**				
**1X9M-A**	-27.4	12.2	-114.71832	51.07896
**1X9M-B**	-0.6	2.8	-2.51208	11.72304
**1XS9-A**				
**1XS9-D**				
**1YNW-A**	-24.9	6.7	-104.25132	28.05156
**1YNW-B**	-60	4.2	-251.208	17.58456
**2AS5-F**				
**2AS5-N**	-25.4	11.6	-106.34472	48.56688
**2BSQ-A**	-6.6	34.7	-27.63288	145.28196
**2BSQ-E**	-6.6	34.7	-27.63288	145.28196
**2F8X-C**	-25	9.5	-104.67	39.7746
**2F8X-K**				
**2F8X-M**				
**2FO1-A**	-21.4	7.1	-89.59752	29.72628
**2FO1-E**				
**2NLL-A**	-11.5	-0.8	-48.1482	-3.34944
**2NLL-B**	-16.7	10.4	-69.91956	43.54272
