# Supplementary material for: Computational Structural Analysis: Multiple Proteins Bound to DNA
Source: PLoS One. 2008 Sep 19;3(9):e3243. doi: 10.1371/journal.pone.0003243 (PMC2532747; doi:10.1371/journal.pone.0003243)
Supplement: Table S16 — Detailed list of energies for each complex in group-SingleProtein∶DNA (0.04 MB PDF) [file pone.0003243.s023.pdf]

Table S16. Detailed list of energies for each complex in group-SingleProtein:DNA

	deltaG-int (kcal/mol)	deltaG-diss (kcal/mol)	deltaG-int (kJ/mol)	deltaG-diss (kJ/mol)
1A0A	-45.8	18.1	-191.75544	75.78108
1A3Q	-36.5	20.7	-152.8182	86.66676
1AM9	-22.4	20.8	-93.78432	87.08544
1B01				
1B3T	-55.2	30.2	-231.11136	126.44136
1BDT	-49	21.2	-205.1532	88.76016
1BG1	-8.9	0.4	-37.26252	1.67472
1BL0	-39.4	25.5	-164.95992	106.7634
1BPY	-24	13.2	-100.4832	55.26576
1C8C	-15.4	0.5	-64.47672	2.0934
1CEZ	-20.7	16.6	-86.66676	69.50088
1CKT	-18	1.2	-75.3624	5.02416
1CL8	-15.9	10.9	-66.57012	45.63612
1CW0	-30.2	2.8	-126.44136	11.72304
1D02	-48.4	27	-202.64112	113.0436
1DC1	-47.9	28.5	-200.54772	119.3238
1DDN	-54.1	27.8	-226.50588	116.39304
1DEW	-15	2.8	-62.802	11.72304
1DFM	-53.8	33.7	-225.24984	141.09516
1DH3	-42.4	21.9	-177.52032	91.69092
1DIZ	-12.5	2.5	-52.335	10.467
1DMU	-7.6	5.4	-31.81968	22.60872
1DP7	-3.2	-2.1	-13.39776	-8.79228
1E3O				
1ECR	-45.1	28.7	-188.82468	120.16116
1EFA	-42.3	10.8	-177.10164	45.21744
1EGW	-51.6	9.3	-216.03888	38.93724
1ESG	-18.4	-1.3	-77.03712	-5.44284
1EWN	-15.9	5.7	-66.57012	23.86476
1EWQ	-34	15.8	-142.3512	66.15144
1EYG	-5.8	0.9	-24.28344	3.76812
1F44	-30.7	30.8	-128.53476	128.95344
1F4K	-60.8	43.5	-254.55744	182.1258
1FOK	-31.3	27.3	-131.04684	114.29964
1FZP	-26.4	-5.6	-110.53152	-23.44608
1G38	-22.9	15.7	-95.87772	65.73276
1G9Z	-20.9	15.1	-87.50412	63.22068
1GDT	-25	13.9	-104.67	58.19652
1HLV	-36.1	26.8	-151.14348	112.20624
1HWT	-35	12.8	-146.538	53.59104
1I3J	-39	30	-163.2852	125.604
1I6J	-4.5	-8.4	-18.8406	-35.16912
1I7D	-15.6	18.7	-65.31408	78.29316
1IAW	-13.1	-1.2	-54.84708	-5.02416
1IC8	-29.1	16.7	-121.83588	69.91956
1IGN	-25	32.2	-104.67	134.81496
1J1V	-17.8	9.7	-74.52504	40.61196
1JB7	-21.7	4.6	-90.85356	19.25928
1JE8	-53.7	43.9	-224.83116	183.80052
1JJ4	-43.3	40.6	-181.28844	169.98408
1JMC	-5.7	1.9	-23.86476	7.95492
1JT0	-35.1	16.6	-146.95668	69.50088
1JX4	-35	29.4	-146.538	123.09192
1K3X	-10.5	2.9	-43.9614	12.14172
1K4T	-21	3.2	-87.9228	13.39776
1KC6	-142.1	-17.5	-594.94428	-73.269
1KDH	0	0	0	0
1KU7	-29.8	5.4	-124.76664	22.60872
1L3L	-9.8	1	-41.03064	4.1868
1L3S	-17.3	15.7	-72.43164	65.73276
1LLM	-52.5	40.1	-219.807	167.89068
1LMB	-48.2	27.6	-201.80376	115.55568
1LQ1	-36.2	5.9	-151.56216	24.70212
1LRR	-11	1.9	-46.0548	7.95492
1LWY	-17.6	6.1	-73.68768	25.53948
1M5R	11.6	2.9	48.56688	12.14172
1MHD	-16.6	3.9	-69.50088	16.32852
1MJO	-33.9	9.9	-141.93252	41.44932
1MNN	-17.1	8.7	-71.59428	36.42516

**1MUS**	-11.9	0.7	-49.82292	2.93076
**1MW8**	-15.9	12.1	-66.57012	50.66028
**1MWI**	-7.2	0.5	-30.14496	2.0934
**1ODH**	-23.4	13.8	-97.97112	57.77784
**1OE4**	-13.3	-1	-55.68444	-4.1868
**1ORN**	-15.5	11	-64.8954	46.0548
**1OUP**	-11.7	0.5	-48.98556	2.0934
**1P4E**	-43.3	35.9	-181.28844	150.30612
**1P71**	-64.6	-0.1	-270.46728	-0.41868
**1P7H**	-26.1	4.6	-109.27548	19.25928
**1PV4**	-2.1	0.8	-8.79228	3.34944
**1QNA**	-14	0	-58.6152	0
**1QPZ**	-9.2	2.3	-38.51856	9.62964
**1QRV**	-13	-8.4	-54.4284	-35.16912
**1QUM**	-11.4	2.2	-47.72952	9.21096
**1REP**	-26.4	17.7	-110.53152	74.10636
**1SKN**	-17.2	8.2	-72.01296	34.33176
**1TC3**	-24.8	13.6	-103.83264	56.94048
**1TRO**	-67.1	22.5	-280.93428	94.203
**1TUP**	-16	-0.3	-66.9888	-1.25604
**1UBD**	-33.5	19.1	-140.2578	79.96788
**1VAS**	-12.3	8.1	-51.49764	33.91308
**1ZME**	-40.1	15.2	-167.89068	63.63936
**2BOP**	10.6	3.7	44.38008	15.49116
**2CGP**	-63.1	0.6	-264.18708	2.51208
**2DRP**	-25	13.5	-104.67	56.5218
**2HDD**	-28.8	3	-120.57984	12.5604
**2IRF**	-28.8	4.2	-120.57984	17.58456
**2PJR**	-55.1	0.1	-230.69268	0.41868
**3HTS**	-9.3	3.1	-38.93724	12.97908
**3PVI**	-11.9	7.4	-49.82292	30.98232
**6CRO**				
**6MHT**	-16.8	0.5	-70.33824	2.0934
