# Supplementary material for: Computational Structural Analysis: Multiple Proteins Bound to DNA
Source: PLoS One. 2008 Sep 19;3(9):e3243. doi: 10.1371/journal.pone.0003243 (PMC2532747; doi:10.1371/journal.pone.0003243)
Supplement: Table S17 — Detailed list of energies for each complex in group-SingleSameProtein∶DNA (0.04 MB PDF) [file pone.0003243.s024.pdf]

Table S17. Detailed list of energies for each complex in group-SingleSameProtein:DNA

	deltaG-int (kcal/mol)	deltaG-diss (kcal/mol)	deltaG-int (kJ/mol)	deltaG-diss (kJ/mol)
**1A66**	-22	6.2	-92.1096	25.95816
**2H7H**	-39.6	16.3	-165.79728	68.24484
**1LFU**	-11.1	0.3	-46.47348	1.25604
**1TGH**	-18.8	9.4	-78.71184	39.35592
**1GU4**	-45.7	20.3	-191.33676	84.99204
**1BC8**	-22.4	13.9	-93.78432	58.19652
**1Y05**	-14.9	8.5	-62.38332	35.5878
**2RAM**	-16.6	5.2	-69.50088	21.77136
**1K61**	-18.1	-3.9	-75.78108	-16.32852
**1YTB**	-5.4	1.3	-22.60872	5.44284
**1TTU**	-20.1	11.1	-84.15468	46.47348
**1P7H**	-20.2	10.6	-84.57336	44.38008
**1KB2**	-22.5	8.7	-94.203	36.42516
**1U8B**	-11.5	0.8	-48.1482	3.34944
**1KU7**	-16.5	7.4	-69.0822	30.98232
**1C7U**	-65.5	10	-274.2354	41.868
**9ANT**	-34.3	0	-143.60724	0
