# Supplementary material for: Computational Structural Analysis: Multiple Proteins Bound to DNA
Source: PLoS One. 2008 Sep 19;3(9):e3243. doi: 10.1371/journal.pone.0003243 (PMC2532747; doi:10.1371/journal.pone.0003243)
Supplement: Table S18 — Detailed list of energies for each complex in group-SubSetMultiProteins∶DNA (0.04 MB PDF) [file pone.0003243.s025.pdf]

Table S18. Detailed list of energies for each complex in group-SubSetMultiProteins:DNA

	deltaG-int (kcal/mol)	deltaG-diss (kcal/mol)	deltaG-int (kJ/mol)	deltaG-diss (kJ/mol)
**1A02**	-48.1	20.5	-201.38508	85.8294
**1B72**	-33.1	12.8	-138.58308	53.59104
**1B8I**	-27.4	5.3	-114.71832	22.19004
**1D3U**	-46.6	13.8	-195.10488	57.77784
**1H8A**	-71	17.1	-297.2628	71.59428
**1HJB**	-59.2	3.4	-247.85856	14.23512
**1IO4**	-68.1	3	-285.12108	12.5604
**1JFI**	-80.5	7.3	-337.0374	30.56364
**1K6O**	-88.3	12.6	-369.69444	52.75368
**1K78**	-67.1	7.8	-280.93428	32.65704
**1LE5**	-39.4	14	-164.95992	58.6152
**1MNM**	-103.4	1.3	-432.91512	5.44284
**1PUF**	-34.3	10.5	-143.60724	43.9614
**1RIO**	-66.6	4.3	-278.84088	18.00324
**1T2K**	-92.5	7.7	-387.279	32.23836
**1XS9**				
**1YNW**	-32.7	4	-136.90836	16.7472
**2AS5**	-43.9	16.9	-183.80052	70.75692
**2FO1**	-34.7	7	-145.28196	29.3076
