# Supplementary material for: Computational Structural Analysis: Multiple Proteins Bound to DNA
Source: PLoS One. 2008 Sep 19;3(9):e3243. doi: 10.1371/journal.pone.0003243 (PMC2532747; doi:10.1371/journal.pone.0003243)
Supplement: Table S19 — Detailed list of energies Z-scores (direct and indirect readouts) for each complex in group-MultiProteins∶DNA (0.04 MB PDF) [file pone.0003243.s026.pdf]

Table S19. Detailed list of energies Z-scores (direct and indirect readouts) for each complex in group-MultiProteins:DNA

	Z-score (Direct Readout)	Z-score (Indirect Readout)
**1A02**	-6.55	-2.37
**1AKH**	-5.29	-2.2
**1AWC**		
**1B72**	-2.63	-2.13
**1B8I**	-2.29	-1.9
**1CF7**	-5.32	-4.08
**1CQT**	-2.62	-0.96
**1D3U**	-3.46	-1.71
**1DSZ**	-3.75	-3.49
**1FOS**	-3.56	-1.87
**1GT0**	-3.14	-2.35
**1H8A**	-2.41	-2.95
**1H9D**	0.24	-1.56
**1HBX**	-4.37	-3.33
**1HJB**	-1.39	-2
**1IO4**	-0.4	-2.19
**1JEY**	-3.58	-1.67
**1JFI**	-2.4	-1.35
**1K6O**	-4.2	-3
**1K78**	-1.65	-3.87
**1LB2**	-2.08	-1.83
**1LE5**	-3.09	-1.54
**1LE8**	-5.08	-2.23
**1MDM**	-1.22	-3.85
**1MNM**	-6.09	-3.63
**1N6J**	-1.94	-2.94
**1NGM**	-3.17	0.22
**1NH2**	-4.12	-1.57
**1NKP**	-1.26	-2.95
**1NLW**	-1.53	-2.52
**1O4X**	-3.05	-2.05
**1OUZ**	-6.24	-2.98
**1PUF**	-1.74	-1.44
**1R0O**	-3.5	-3.63
**1RIO**	-2.57	-4.04
**1RZR**	-2.21	-1.01
**1T2K**	-2.41	-3.74
**1TQE**	-2.03	-1.53
**1X9M**	-0.01	-1.56
**1XS9**	-2.32	-1.24
**1YNW**	-2.75	-2.54
**2AS5**	-4.16	-1.65
**2BSQ**	1.8	-2.65
**2F8X**	-1.33	-2.54
**2FO1**	-1.88	-2.83
**2NLL**	-3.9	-2.87
