# Supplementary material for: Computational Structural Analysis: Multiple Proteins Bound to DNA
Source: PLoS One. 2008 Sep 19;3(9):e3243. doi: 10.1371/journal.pone.0003243 (PMC2532747; doi:10.1371/journal.pone.0003243)
Supplement: Table S20 — Detailed list of energies Z-scores (direct and indirect readouts) for each complex in group-SubMultiProteins∶DNA (0.04 MB PDF) [file pone.0003243.s027.pdf]

Table S20. Detailed list of energies Z-scores (direct and indirect readouts) for each complex in group-SubMultiProteins:DNA

	Z-score (Direct Readout)
1A02-F	-3.25
1A02-J	-3.27
1A02-N	-4.54
1AKH-A	-4.23
1AKH-B	-3.36
1B72-A	-2.06
1B72-B	-1.27
1B8I-A	-2.57
1B8I-B	-0.13
1CF7-A	-5.44
1CF7-B	-1.65
1CQT-B	-3.03
1CQT-J	2.97
1D3U-A	-2.97
1D3U-B	-1.97
1DSZ-A	-2.78
1DSZ-B	-1.94
1FOS-G	-1.96
1FOS-H	-2.8
1GT0-C	-4.42
1GT0-D	-0.26
1H8A-A,B	-2.05
1H8A-C	-0.95
1HBX-A,B	-4.71
1HBX-G	0.34
1HJB-D,E	-1.16
1HJB-F	-0.33
1IO4-A,B	-0.45
1IO4-C	0.36
1JEY-A	-3.82
1JEY-B	-4.24
1JFI-A	2.62
1JFI-B	2.08
1JFI-C	-3.38
1K6O-A	0.91
1K6O-B,C	-4.99
1K78-A,I	-0.19
1K78-B	-1.54
1LB2-A	-2.32
1LB2-B,E	-0.67
1LE5-A	-1.16
1LE5-B	-2.61
1LE8-A	-3.94
1LE8-B	-3.51
1MDM-A	-0.1
1MDM-B	-1.11
1MNM-A,B	-5.2
1MNM-C,D	-3.09
1N6J-A,B	-1.94
1N6J-G	
1NGM-A	-3.18
1NGM-B	0.6
1NH2-A,B	-4.01
1NH2-B,C	-0.01
1NKP-A	0.77

**1NKP-B**	-2.02
**1NLW-A**	-0.83
**1NLW-B**	-1.04
**1O4X-A**	-3.21
**1O4X-B**	-1.18
**1OUZ-A**	-5.35
**1OUZ-B**	-4.92
**1PUF-A**	-1.24
**1PUF-B**	-1.21
**1R0O-A**	-2.78
**1R0O-B**	-1.96
**1RIO-A,B**	-2.67
**1RIO-H**	0.38
**1RZR-A,D**	-2.21
**1RZR-S,Y**	
**1T2K-A,B**	-2.32
**1T2K-C,D**	0.01
**1TQE-S,R**	-2.01
**1TQE-Y**	0.16
**1X9M-A**	-0.01
**1X9M-B**	
**1XS9-A**	-2.32
**1XS9-D**	1
**1YNW-A**	-0.36
**1YNW-B**	-2.78
**2AS5-F**	-2.49
**2AS5-N**	-3.18
**2BSQ-A**	3.99
**2BSQ-E**	0.55
**2F8X-C**	-1.2
**2F8X-K**	0.35
**2F8X-M**	-0.85
**2FO1-A**	-1.95
**2FO1-E**	2.38
**2NLL-A**	-2.59
**2NLL-B**	-2.77
