# Supplementary material for: Computational Structural Analysis: Multiple Proteins Bound to DNA
Source: PLoS One. 2008 Sep 19;3(9):e3243. doi: 10.1371/journal.pone.0003243 (PMC2532747; doi:10.1371/journal.pone.0003243)
Supplement: Table S21 — Detailed list of energies Z-scores (direct and indirect readouts) for each complex in group-SingleProtein∶DNA (0.04 MB PDF) [file pone.0003243.s028.pdf]

Table S21. Detailed list of energies Z-scores (direct and indirect readouts) for each complex in group-SingleProtein:DNA

	Z-score (Direct Readout)	Z-score (Indirect Readout)
1A0A	-0.79	-0.97
1A3Q	-4.78	-1.06
1AM9	-2.73	-4.43
1B01	-2.09	-0.98
1B3T	-6.01	-3.58
1BDT	-1.82	-1.81
1BG1		
1BL0	-6.88	-3.5
1BPY	-2.4	-1.81
1C8C	0.88	-1.85
1CEZ	-0.74	-1.73
1CKT		
1CL8		
1CW0	0.14	-2.75
1D02	-4.61	-0.07
1DC1	-1.75	-0.32
1DDN	-2.24	-1.09
1DEW	1.45	-1.69
1DFM	-2.68	-2.14
1DH3	-2.48	-1.62
1DIZ	-0.86	-1.4
1DMU		
1DP7		
1E3O	-4.01	-2.66
1ECR	-6.12	-1.48
1EFA	-2.77	-1.39
1EGW		
1ESG	0.69	-1.09
1EWN	-0.77	-1.59
1EWQ	2.02	-1.75
1EYG		
1F44	-0.3	-2.51
1F4K	-2.7	-2.86
1FOK	-0.86	-3.3
1FZP	-0.71	-1.7
1G38		
1G9Z	1.13	-2.1
1GDT	-5.71	-3.05
1HLV	-0.46	-2.91
1HWT	-5.71	-3.13
1I3J	0.72	-2.44
1I6J	-0.15	-1.45
1I7D		
1IAW	1.14	-3.49
1IC8	-2.59	-2.47
1IGN	-6.02	-3.65
1J1V	-1.23	-2.26
1JB7		-0.32
1JE8	-2.22	-3.9
1JJ4	-0.85	-2.42
1JMC		
1JT0	0.49	-3.32
1JX4	-0.64	-3.28
1K3X		
1K4T	-1.68	-4.57

**1KC6**	0.87	0.19
**1KDH**		
**1KU7**	0.57	-2.14
**1L3L**	-1.53	-3.07
**1L3S**	0.15	-1.8
**1LLM**	-4.12	-1.78
**1LMB**	-5.31	-4.52
**1LQ1**	-2.82	-3.5
**1LRR**		
**1LWY**	-0.22	-2.58
**1M5R**	-2.32	-2.25
**1MHD**	-4.54	-2.53
**1MJO**	-3.51	-2.59
**1MNN**	-0.73	-1.96
**1MUS**	0.21	-3.27
**1MW8**		
**1MWI**		
**1ODH**	0.24	-2.51
**1OE4**	1.22	-2.23
**1ORN**	0.38	-2.62
**1OUP**	1.2	-1.89
**1P4E**		-2.45
**1P71**	-2.39	-0.04
**1P7H**	-2.67	-2.13
**1PV4**		
**1QNA**		0.44
**1QPZ**	1.31	-1.76
**1QRV**	0.33	-0.85
**1QUM**	1.31	-1.76
**1REP**	-6.62	-3.75
**1SKN**	-2.55	-1.98
**1TC3**	-4.9	-4.43
**1TRO**	-5.88	-3.65
**1TUP**	-4.61	-1.51
**1UBD**	-5.74	-2.25
**1VAS**	-0.18	-0.32
**1ZME**	1.02	-1.82
**2BOP**		
**2CGP**	0.13	-1.49
**2DRP**	-4.2	-3.24
**2HDD**	-3.72	-2.34
**2IRF**		
**2PJR**		
**3HTS**		
**3PVI**	-2.3	0.12
**6CRO**	-6.54	0.26
**6MHT**	2.37	-1.53
