# Supplementary material for: Computational Structural Analysis: Multiple Proteins Bound to DNA
Source: PLoS One. 2008 Sep 19;3(9):e3243. doi: 10.1371/journal.pone.0003243 (PMC2532747; doi:10.1371/journal.pone.0003243)
Supplement: Table S22 — Detailed list of energy Z-scores (direct and indirect readouts) for each complex in group-SingleSameProtein∶DNA (0.04 MB PDF) [file pone.0003243.s029.pdf]

Table S22. Detailed list of energy Z-scores (direct and indirect readouts) group-SingleSameProtein:DNA

	Z-score (Direct Readout)	Z-score (Indirect Readout)
**1A66**	-1.87	-0.56
**2H7H**	-2.25	-3.66
**1LFU**	-1.18	-0.25
**1TGH**	-2.67	0.72
**1GU4**	-1.04	-1.96
**1BC8**	-1.34	-1.04
**1Y05**	-0.19	-2.88
**2RAM**	-0.74	-1.3
**1K61**		-2.37
**1YTB**	-3.43	0.35
**1TTU**	-1.85	-2.11
**1P7H**	-0.65	-2.58
**1KB2**	-0.8	-2.53
**1U8B**	-1.83	-0.66
**1KU7**	0.57	-2.14
**1C7U**	-2.11	-0.02
**9ANT**	-0.07	-2.09
