# Supplementary material for: Computational Structural Analysis: Multiple Proteins Bound to DNA
Source: PLoS One. 2008 Sep 19;3(9):e3243. doi: 10.1371/journal.pone.0003243 (PMC2532747; doi:10.1371/journal.pone.0003243)
Supplement: Table S23 — Detailed list of energy Z-scores (direct and indirect readouts) for each complex in group-SubSetMultiProteins∶DNA (0.04 MB PDF) [file pone.0003243.s030.pdf]

Table S23. Detailed list of energy Z-scores (direct and indirect readouts) for each complex in group-SubSetMultiProteins:DNA

	Z-score (Direct Readout)	Z-score (Indirect Readout)
**1A02**	-6.55	-2.37
**1B72**	-2.63	-2.13
**1B8I**	-2.29	-1.9
**1D3U**	-3.46	-1.71
**1H8A**	-2.41	-2.95
**1HJB**	-1.39	-2
**1IO4**	-0.4	-2.19
**1JFI**	-2.4	-1.35
**1K6O**	-4.2	-3
**1K78**	-1.65	-3.87
**1LE5**	-3.09	-1.54
**1MNM**	-6.09	-3.63
**1PUF**	-1.74	-1.44
**1RIO**	-2.57	-4.04
**1T2K**	-2.41	-3.74
**1XS9**	-2.32	-1.24
**1YNW**	-2.75	-2.54
**2AS5**	-4.16	-1.65
**2FO1**	-1.88	-2.83
