# Supplementary material for: Computational Structural Analysis: Multiple Proteins Bound to DNA
Source: PLoS One. 2008 Sep 19;3(9):e3243. doi: 10.1371/journal.pone.0003243 (PMC2532747; doi:10.1371/journal.pone.0003243)
Supplement: Table S24 — Detailed list of protein-DNA energy binding affinity, overlapping volume and number of atoms in collision for each complex in group-MultiProteins∶DNA (0.04 MB PDF) [file pone.0003243.s031.pdf]

Table S24. Detailed list of protein-DNA energy binding affinity, overlapping volume and number of atoms in collision for each complex in group-MultiProteins:DNA

	Protein-DNA energy binding affinity (kcal/mol)	Protein-DNA energy binding affinity (kJ/mol)	Overlapping volume	# Atoms in collision
**1A02**	-9.78	-40.946904	1.78	37
**1AKH**	-8.97	-37.555596	4.255	30
**1AWC**	-7.52	-31.484736	0.999	19
**1B72**	-8.84	-37.011312	3.264	25
**1B8I**	-8.63	-36.132084	5.265	23
**1CF7**	-8.09	-33.871212	1.389	17
**1CQT**	-9.24	-38.686032	2.859	35
**1D3U**	-12.07	-50.534676	2.225	25
**1DSZ**	-8.69	-36.383292	0.571	20
**1FOS**	-7.79	-32.615172	0	0
**1GT0**	-11.4	-47.72952	5.307	49
**1H8A**	-10.4	-43.54272	2.619	17
**1H9D**	-6.89	-28.847052	1.507	13
**1HBX**	-11.76	-49.236768	12.341	66
**1HJB**	-9.99	-41.826132	5.027	32
**1IO4**	-10.04	-42.035472	4.773	25
**1JEY**	-9.04	-37.848672	2.414	18
**1JFI**	-11.09	-46.431612	1.576	18
**1K6O**	-10.91	-45.677988	3.228	42
**1K78**	-12.37	-51.790716	2.092	38
**1LB2**	-8.26	-34.582968	3.617	31
**1LE5**	-8.98	-37.597464	8.662	62
**1LE8**	-8.55	-35.79714	3.291	17
**1MDM**	-11.2	-46.89216	3.409	32
**1MNM**	-11.87	-49.697316	3.601	36
**1N6J**	-7.67	-32.112756	9.375	92
**1NGM**	-9.77	-40.905036	1.129	12
**1NH2**	-9.48	-39.690864	0.941	9
**1NKP**	-8.42	-35.252856	3.45	20
**1NLW**	-8.18	-34.248024	2.717	20
**1O4X**	-10.48	-43.877664	33.869	171
**1OUZ**	-9.98	-41.784264	2.944	31
**1PUF**	-9.71	-40.653828	2.552	27
**1R0O**	-8.82	-36.927576	2.534	25
**1RIO**	-10.03	-41.993604	3.592	35
**1RZR**	-9.06	-37.932408	6.163	38
**1T2K**	-12.23	-51.204564	6.621	39
**1TQE**	-7.98	-33.410664	1.179	14
**1X9M**	-8.61	-36.048348	2.788	29
**1XS9**	-8.42	-35.252856	16.36	87
**1YNW**	-8.6	-36.00648	3.964	27
**2AS5**	-9.27	-38.811636	3.745	26
**2BSQ**	-6.46	-27.046728	2.312	8
**2F8X**	-7.52	-31.484736	0.55	10
**2FO1**	-7.31	-30.605508	1.055	10
**2NLL**	-8.61	-36.048348	2.225	18
