# Supplementary material for: Computational Structural Analysis: Multiple Proteins Bound to DNA
Source: PLoS One. 2008 Sep 19;3(9):e3243. doi: 10.1371/journal.pone.0003243 (PMC2532747; doi:10.1371/journal.pone.0003243)
Supplement: Table S25 — Detailed list of protein-DNA energy binding affinity, overlapping volume and number of atoms in collision for each complex in group-SubMultiProteins∶DNA (0.05 MB PDF) [file pone.0003243.s032.pdf]

Table S25. Detailed list of protein-DNA energy binding affinity, overlapping volume and number of atoms in collision for each complex in group-SubMultiProteins:DNA

	Protein-DNA energy binding affinity (kcal/mol)	Protein-DNA energy binding affinity (kJ/mol)	Overlapping volume	# Atoms in collision
1A02-F	-6.79	-28.428372	0.57	10
1A02-J	-6.58	-27.549144	0.247	3
1A02-N	-7.36	-30.814848	0.964	24
1AKH-A	-6.91	-28.930788	2.132	12
1AKH-B	-7.53	-31.526604	2.132	18
1AWC-A	-7.43	-31.107924	0.999	19
1AWC-B	-5.56	-23.278608	0	0
1B72-A	-7.05	-29.51694	1.69	12
1B72-B	-7.27	-30.438036	1.57	13
1B8I-A	-7.15	-29.93562	3.32	14
1B8I-B	-6.96	-29.140128	1.94	9
1CF7-A	-6.87	-28.763316	1.11	13
1CF7-B	-6.7	-28.05156	0.27	4
1CQT-B	-8.62	-36.090216	2.82	32
1CQT-J	-6.09	-25.497612	0.04	3
1D3U-A	-9.83	-41.156244	0.73	12
1D3U-B	-7.7	-32.23836	1.5	13
1DSZ-A	-7.04	-29.475072	0.3	9
1DSZ-B	-7.12	-29.810016	0.27	11
1FOS-G	-6.69	-28.009692	0	0
1FOS-H	-6.57	-27.507276	0	0
1GT0-C	-8.62	-36.090216	2.89	27
1GT0-D	-8.36	-35.001648	2.42	22
1H8A-A,B	-8.26	-34.582968	2.62	17
1H8A-C	-7.62	-31.903416	1.52	12
1HBX-A,B	-9.51	-39.816468	4.93	34
1HBX-G	-7.73	-32.363964	7.41	32
1HJB-D,E	-8.34	-34.917912	1.99	14
1HJB-F	-7.12	-29.810016	3.04	18
1IO4-A,B	-8.32	-34.834176	1.61	11
1IO4-C	-7.21	-30.186828	3.17	14
1IO4-D	-5.47	-22.901796	0	0
1JEY-A	-7.1	-29.72628	0.34	2
1JEY-B	-7.41	-31.024188	2.08	16
1JFI-A	-5.93	-24.827724	0.22	4
1JFI-B	-6.43	-26.921124	0.23	3
1JFI-C	-9.68	-40.528224	1.12	11
1K6O-A	-7.52	-31.484736	1.07	10
1K6O-B,C	-8.86	-37.095048	2.16	32
1K78-A,I	-10.63	-44.505684	1.79	27
1K78-B	-7.21	-30.186828	0.3	11
1LB2-A	-6.95	-29.09826	1.74	17
1LB2-B,E	-6.79	-28.428372	1.87	14
1LE5-A	-7.12	-29.810016	3.99	28
1LE5-B	-7.33	-30.689244	4.67	34
1LE8-A	-6.93	-29.014524	1.26	10
1LE8-B	-7.09	-29.684412	2.02	7
1MDM-A	-9.24	-38.686032	2.06	23
1MDM-B	-7.44	-31.149792	1.344	9
1MNM-A,B	-8.33	-34.876044	1.69	18
1MNM-C,D	-9.02	-37.764936	1.9	18
1N6J-A,B	-7.67	-32.112756	9.38	92
1N6J-G	-5.47	-22.901796	0	0

**1NGM-A**	-9.71	-40.653828	1.13	12
**1NGM-B**	-5.53	-23.153004	0	0
**1NH2-A,B**	-9.24	-38.686032	0.68	7
**1NH2-C,D**	-5.72	-23.948496	0.26	2
**1NKP-A**	-6.95	-29.09826	2.67	10
**1NKP-B**	-6.94	-29.056392	0.78	10
**1NLW-A**	-6.76	-28.302768	1.16	12
**1NLW-B**	-6.89	-28.847052	1.55	8
**1O4X-A**	-7.69	-32.196492	11.59	69
**1O4X-B**	-8.26	-34.582968	22.27	102
**1OUZ-A**	-7.93	-33.201324	0.74	8
**1OUZ-B**	-7.52	-31.484736	1.48	17
**1PUF-A**	-7.91	-33.117588	1.92	17
**1PUF-B**	-7.27	-30.438036	0.63	10
**1R0O-A**	-7.09	-29.684412	0.19	5
**1R0O-B**	-7.21	-30.186828	0.65	4
**1RIO-A,B**	-8.76	-36.676368	3.51	27
**1RIO-H**	-6.74	-28.219032	0.08	8
**1RZR-A,D**	-9.06	-37.932408	6.16	38
**1RZR-S,Y**	-5.47	-22.901796	0	0
**1T2K-A,B**	-9.98	-41.784264	3.85	30
**1T2K-C,D**	-7.72	-32.322096	2.77	9
**1TQE-S,R**	-7.98	-33.410664	1.18	14
**1TQE-Y**	-5.47	-22.901796	0	0
**1X9M-A**	-8.61	-36.048348	2.79	29
**1X9M-B**	-5.47	-22.901796	0	0
**1XS9-A**	-8.45	-35.37846	16.36	87
**1XS9-D**			0	0
**1YNW-A**	-7.26	-30.396168	1.82	14
**1YNW-B**	-6.82	-28.553976	2.15	13
**2AS5-F**	-7.28	-30.479904	1.72	14
**2AS5-N**	-7.47	-31.275396	2.03	12
**2BSQ-A**	-5.61	-23.487948	0.38	2
**2BSQ-E**	-6.32	-26.460576	1.93	6
**2F8X-C**	-7.52	-31.484736	0.55	10
**2F8X-K**	-5.47	-22.901796	0	0
**2F8X-M**	-7.67	-32.112756	0	0
**2FO1-A**	-7.31	-30.605508	1.05	10
**2FO1-E**	-5.46	-22.859928	0	0
**2NLL-A**	-6.68	-27.967824	1.23	7
**2NLL-B**	-7.41	-31.024188	0.99	11
