# Supplementary material for: Computational Structural Analysis: Multiple Proteins Bound to DNA
Source: PLoS One. 2008 Sep 19;3(9):e3243. doi: 10.1371/journal.pone.0003243 (PMC2532747; doi:10.1371/journal.pone.0003243)
Supplement: Table S26 — Detailed list of protein-DNA energy binding affinity, overlapping volume and number of atoms in collision for each complex in group-SingleProtein∶DNA (0.05 MB PDF) [file pone.0003243.s033.pdf]

Table S26. Detailed list of protein-DNA energy binding affinity, overlapping volume and number of atoms in collision for each complex in group-SingleProtein:DNA

	Protein-DNA energy binding affinity (kcal/mol)	Protein-DNA energy binding affinity (kJ/mol)	Overlapping volume	# Atoms in collision
1A0A	-5.47	-22.901796	15.745	61
1A3Q	-8.52	-35.671536	1.52	18
1AM9	-8.85	-37.05318	1.677	11
1B01	-6.97	-29.181996	5.001	31
1B3T	-10.87	-45.510516	1.788	37
1BDT	-7.9	-33.07572	1.315	17
1BG1	-6.19	-25.916292	0.367	8
1BL0	-8.4	-35.16912	3.439	23
1BPY	-8.4	-35.16912	0.622	12
1C8C	-7.13	-29.851884	5.072	21
1CEZ	-9.68	-40.528224	4.042	29
1CKT	-7.59	-31.777812	0.873	15
1CL8	-6.68	-27.967824	0.422	12
1CW0	-9.52	-39.858336	1.266	24
1D02	-9.02	-37.764936	1.383	24
1DC1	-11.79	-49.362372	4.879	51
1DDN	-8.46	-35.420328	1.82	16
1DEW	-5.47	-22.901796	1.627	16
1DFM	-12.2	-51.07896	2.142	27
1DH3	-8	-33.4944	1.502	14
1DIZ	-5.47	-22.901796	0.499	5
1DMU	-7.17	-30.019356	2.4	24
1DP7	-6.26	-26.209368	1.6	20
1E3O	-7.77	-32.531436	1.941	21
1ECR	-10.69	-44.756892	1.931	33
1EFA	-7.46	-31.233528	1.949	16
1EGW	-7.64	-31.987152	3.696	56
1ESG	-6.88	-28.805184	1.238	6
1EWN	-7.22	-30.228696	2.517	16
1EWQ	-7.83	-32.782644	1.304	16
1EYG	-8.76	-36.676368	49.966	113
1F44	-10.31	-43.165908	6.283	40
1F4K	-9.42	-39.439656	1.825	25
1FOK	-9.98	-41.784264	3.118	24
1FZP	-6.52	-27.297936	2.261	11
1G38	-9.42	-39.439656	1.593	23
1G9Z	-7.55	-31.61034	1.384	15
1GDT	-6.43	-26.921124	0	0
1HLV	-9.19	-38.476692	2.554	24
1HWT	-7.75	-32.4477	2.63	18
1I3J	-9.83	-41.156244	2.39	20
1I6J	-6.3	-26.37684	0.931	5
1I7D	-8.61	-36.048348	4.468	44
1IAW	-7.95	-33.28506	3.684	27
1IC8	-8.39	-35.127252	3.571	24
1IGN	-9.87	-41.323716	7.414	67
1J1V	-7.41	-31.024188	1.088	15
1JB7	-9.63	-40.318884	2.474	33
1JE8	-8.55	-35.79714	1.04	15
1JJ4	-8.24	-34.499232	3.176	27
1JMC	-7.66	-32.070888	16.426	104
1JT0	-8.51	-35.629668	3.994	34
1JX4	-8.87	-37.136916	4.864	25

1K3X	-7.07	-29.600676	14.295	20
1K4T	-9.2	-38.51856	11.286	16
1KC6	-8.24	-34.499232	1.232	14
1KDH	-5.47	-22.901796	2.5	24
1KU7	-6.75	-28.2609	1.011	8
1L3L	-6.15	-25.74882	0.311	1
1L3S	-9.86	-41.281848	1.313	22
1LLM	-9.74	-40.779432	3.492	33
1LMB	-8.63	-36.132084	0	0
1LQ1	-7.95	-33.28506	0.382	15
1LRR	-7.04	-29.475072	3.451	17
1LWY	-7.67	-32.112756	12.01	18
1M5R	-7.31	-30.605508	0.427	15
1MHD	-6.69	-28.009692	0.343	3
1MJO	-7.36	-30.814848	1.669	29
1MNN	-8.21	-34.373628	2.077	19
1MUS	-8.73	-36.550764	1.556	20
1MW8	-7.45	-31.19166	7.336	34
1MWI	-5.74	-24.032232	1.92	10
1ODH	-7.52	-31.484736	1.374	12
1OE4	-6.39	-26.753652	0.09	2
1ORN	-7.51	-31.442868	10.871	21
1OUP	-6.17	-25.832556	0.042	6
1P4E	-7.66	-32.070888	3.572	20
1P71	-8.55	-35.79714	2.366	13
1P7H	-9.27	-38.811636	3.664	24
1PV4	-6.07	-25.413876	1.191	10
1QNA	-9.89	-41.407452	0.176	7
1QPZ	-6.27	-26.251236	1.878	20
1QRV	-8.04	-33.661872	0.618	11
1QUM	-7.36	-30.814848	0.599	12
1REP	-8.49	-35.545932	3.434	19
1SKN	-7.25	-30.3543	4.888	12
1TC3	-7.31	-30.605508	2.253	21
1TRO	-8.26	-34.582968	1.41	9
1TUP	-5.73	-23.990364	0	0
1UBD	-9.01	-37.723068	1.935	21
1VAS	-8.53	-35.713404	0	0
1ZME	-7.98	-33.410664	1.774	17
2BOP	-6.24	-26.125632	0	0
2CGP	-6.53	-27.339804	0.258	6
2DRP	-7.7	-32.23836	0	0
2HDD	-7.7	-32.23836	1.707	20
2IRF	-8.4	-35.16912	1.143	25
2PJR	-6.99	-29.265732	1.624	15
3HTS			0.056	2
3PVI	-7.61	-31.861548	1.914	12
6CRO	-6.95	-29.09826	1.892	2
6MHT	-7.81	-32.698908	3.408	16
