# Supplementary material for: Computational Structural Analysis: Multiple Proteins Bound to DNA
Source: PLoS One. 2008 Sep 19;3(9):e3243. doi: 10.1371/journal.pone.0003243 (PMC2532747; doi:10.1371/journal.pone.0003243)
Supplement: Table S27 — Detailed list of protein-DNA energy binding affinity, overlapping volume and number of atoms in collision for each complex in group-SingleSameProtein∶DNA (0.04 MB PDF) [file pone.0003243.s034.pdf]

Table S27. Detailed list of protein-DNA energy binding affinity, overlapping volume and number of atoms in collision for each complex in group-SingleSameProtein:DNA

	Protein-DNA energy binding affinity (kcal/mol)	Protein-DNA energy binding affinity (kJ/mol)	Overlapping volume	# Atoms in collision
**1A66**	-7.72	-32.322096	-	-
**2H7H**	-7.94	-33.243192	2.45	16
**1LFU**	-7.36	-30.814848	-	-
**1TGH**	-9.74	-40.779432	11.791	55
**1GU4**	-8.45	-35.37846	1.211	19
**1BC8**	-7.54	-31.568472	0.824	11
**1Y05**	-7.65	-32.02902	0.679	15
**2RAM**	-7.96	-33.326928	2	15
**1K61**	-8	-33.4944	1.38	14
**1YTB**	-9.59	-40.151412	0	0
**1TTU**	-7.75	-32.4477	2.567	18
**1P7H**	-7.39	-30.940452	2.513	15
**1KB2**	-7.4	-30.98232	1.112	10
**1U8B**	-6.44	-26.962992	1.807	14
**1KU7**	-6.75	-28.2609	1.011	8
**1C7U**	-8.36	-35.001648		
**9ANT**	-7.1	-29.72628	3.037	7
