# Supplementary material for: Computational Structural Analysis: Multiple Proteins Bound to DNA
Source: PLoS One. 2008 Sep 19;3(9):e3243. doi: 10.1371/journal.pone.0003243 (PMC2532747; doi:10.1371/journal.pone.0003243)
Supplement: Table S28 — Detailed list of protein-DNA energy binding affinity, overlapping volume and number of atoms in collision for each complex in group-SubSetMultiProteins∶DNA (0.04 MB PDF) [file pone.0003243.s035.pdf]

Table S28. Detailed list of protein-DNA energy binding affinity, overlapping volume and number of atoms in collision for each complex in group-SubSetMultiProteins:DNA

	Protein-DNA energy binding affinity (kcal/mol)	Protein-DNA energy binding affinity (kJ/mol)	Overlapping volume	# Atoms in collision
**1A02**	-9.78	-40.946904	1.78	37
**1B72**	-8.84	-37.011312	3.264	25
**1B8I**	-8.63	-36.132084	5.265	23
**1D3U**	-12.07	-50.534676	2.225	25
**1H8A**	-10.4	-43.54272	2.619	17
**1HJB**	-9.99	-41.826132	5.027	32
**1IO4**	-10.04	-42.035472	4.773	25
**1JFI**	-11.09	-46.431612	1.576	18
**1K6O**	-10.91	-45.677988	3.228	42
**1K78**	-12.37	-51.790716	2.092	38
**1LE5**	-8.98	-37.597464	8.662	62
**1MNM**	-11.87	-49.697316	3.601	36
**1PUF**	-9.71	-40.653828	2.552	27
**1RIO**	-10.03	-41.993604	3.592	35
**1T2K**	-12.23	-51.204564	6.621	39
**1XS9**	-8.42	-35.252856	16.36	87
**1YNW**	-8.6	-36.00648	3.964	27
**2AS5**	-9.27	-38.811636	3.745	26
**2FO1**	-7.31	-30.605508	1.055	10
