# Supplementary material for: Computational Structural Analysis: Multiple Proteins Bound to DNA
Source: PLoS One. 2008 Sep 19;3(9):e3243. doi: 10.1371/journal.pone.0003243 (PMC2532747; doi:10.1371/journal.pone.0003243)
Supplement: Table S29 — Detailed list of protein-protein binding free energy for each protein-proteincomplex in group-MultiProteins∶DNA (0.04 MB PDF) [file pone.0003243.s036.pdf]

Table S29. Detailed list of protein-protein binding free energy for each protein-proteincomplex in group-MultiProteins:DNA

	protein-protein binding free energy (kcal/mol)	protein-protein binding free energy (kJ/mol)	
**1A02: F-J**	-13.362505	-55.94613593	
**1A02: F-N**	-7.088587	-29.67849605	
**1A02: N-J**	-6.789815	-28.42759744	
**1AKH: A-B**	-9.211126	-38.56514234	
**1AWC: A-B**	-17.545782	-73.46068008	
**1B72: A-B**	-9.4	-39.35592	
**1B8I: A-B**	-7.895384	-33.05639373	
**1CF7: A-B**	-13.7021	-57.36795228	
**1CQT: B-J**	-9.049313	-37.88766367	
**1D3U: A-B**	-12.331	-51.6274308	
**1DSZ: A-B**	-5.167206	-21.63405808	
**1FOS: G-H**	-16.880608	-70.67572957	
**1GT0:C-D**	-5	-20.934	
**1H8A**			
**1H9D:C-D**	-17.12	-71.678016	
**1HBX: A,B-G**	-15.4725	-64.780263	
**1HJB**			
**1IO4: A,B-D**	-4.7	-19.67796	
**1IO4: C-D**	-15.7356	-65.88181008	
**1JEY:A-B**			(outlier)-165.486
**1JFI: A-B**	-50.105	-209.779614	
**1JFI: A-C**	-5.510958	-23.07327895	
**1JFI: B-C**	-12.866	-53.8673688	
**1K6O: A-B**	-5.0037	-20.94949116	
**1K78: A,I-B**	-6.323	-26.4731364	
**1LB2:A-B**	-6.569544	-27.50536682	
**1LE5: A-B**	-13.849224	-57.98393104	
**1LE8: A-B**	-10.0738	-42.17698584	
**1MDM: A-B**	-5.9408	-24.87294144	
**1MNM: A,B-C,D**	-15.211	-63.6854148	
**1N6J: A,B-G**			
**1NGM: A-B**	-25.416336	-106.4131156	
**1NH2: A-C,D**	-15.014	-62.8606152	
**1NKP: A-B**	-27.181874	-113.8050701	
**1NLW: A-B**	-23.057724	-96.53807884	
**1O4X: A-B**	-4.8122	-20.14771896	
**1OUZ: A-B**	-59.569	-249.4034892	
**1PUF:A-B**	-8.3709	-35.04728412	
**1R0O: A-B**	-6.480111	-27.13092873	
**1RIO: H-B**	-6.242602	-26.13652605	
**1RZR: A,D-S,Y**	-14.683422	-61.47655123	
**1T2K: B-C**	-4.77	-19.971036	
**1T2K: A-D**	-6.0956	-25.52105808	
**1TQE: R,S-Y**	-15.056488	-63.03850396	
**1X9M: A-B**	-20.981586	-87.84570426	
**1XS9: A-D**	-10.216332	-42.77373882	
**1YNW: A-B**	-4.7	-19.67796	
**2AS5: F-N**	-5.879633	-24.61684744	
**2BSQ: A-E**	-24.95	-104.46066	
**2F8X: K-M**	-8.467	-35.4496356	
**2F8X: M-C**	-15.712565	-65.78536714	
**2F8X: K-C**	-15.531004	-65.02520755	
**2FO1: A-E**	-25.444553	-106.5312545	
**2NLL: A-B**	-5.494899	-23.00604313	
