# Supplementary material for: Computational Structural Analysis: Multiple Proteins Bound to DNA
Source: PLoS One. 2008 Sep 19;3(9):e3243. doi: 10.1371/journal.pone.0003243 (PMC2532747; doi:10.1371/journal.pone.0003243)
Supplement: Table S30 — Detailed list of protein-protein binding free energy for each protein-proteincomplex in group-Protein∶Protein (0.06 MB PDF) [file pone.0003243.s037.pdf]

Table S30. Detailed list of protein-protein binding free energy for each protein-proteincomplex in group-Protein:Protein

	protein-protein binding free energy (kcal/mol)	protein-protein binding free energy (kJ/mol)	deltaG-int (kcal/mol)	deltaG-int (kJ/mol)	deltaG-diss (kcal/mol)	deltaG-diss (kJ/mol)
2PTC: E-I	-15.370447	-64.3529875	-6	-25.1208	3	12.5604
1MCT: A-I	-16.01	-67.030668	-7.1	-29.72628	3.4	14.23512
1AVW: A-B	-19.42	-81.307656	-6.3	-26.37684	0.6	2.51208
3TPI: Z-I	-14.422	-60.3820296	-6.2	-25.95816	2.8	11.72304
1TGS: Z-I	-17.3864	-72.79337952	-9.9	-41.44932	6.4	26.79552
1CHO: E,F,G-I	-14.69	-61.504092	-10.6	-44.38008	4.4	18.42192
1ACB: E-I	-14.037607	-58.77265299	-12.3	-51.49764	5.6	23.44608
1CBW: A,B,C-D	-15.834202	-66.29463693	-67.3	-281.77164	5.1	21.35268
1PPF: E-I	-14.28299	-59.80002253	-12	-50.2416	5.8	24.28344
1FLE: E-I	-16.865053	-70.6106039	-11.6	-48.56688	6.5	27.2142
2KAI: A.B-I	-14.767366	-61.82800797	-54.4	-227.76192	3	12.5604
1HIA: A,B-I	-16.285281	-68.18321449	-55.8	-233.62344	8.2	34.33176
3SGB: E-I	-10.181084	-42.62616249	-8.2	-34.33176	2.6	10.88568
1CSE: E-I	-13.802843	-57.78974307	-8.1	-33.91308	6.7	28.05156
2SIC: E-I	-15.28733	-64.00499324	-14.4	-60.28992	8.4	35.16912
2SNI: E-I	-14.839035	-62.12807174	-11.6	-48.56688	5.7	23.86476
1STF: E-I	-16.75585	-70.15339278	-7.8	-32.65704	2.9	12.14172
4CPA: A-I						
1BTH: J,H-P	-19.432242	-81.35891081	-18.2	-76.19976	6.7	28.05156
4HTC: L,H-I	-17.91	-74.985588	-14.9	-62.38332	5.8	24.28344
1TBQ: J,H-R	-22.72239	-95.13410245	-29	-121.4172	10.7	44.79876
1TOC: A,B-R	-18.316535	-76.68766874	-16	-66.9888	3.5	14.6538
1DAN: L,H-T,U	-22.444854	-93.97211473	-31.8	-133.14024	7.8	32.65704
1JHL: L,H-A	-12.750928	-53.38558535	-11.6	-48.56688	-5.2	-21.77136
1VFB: A,B-C	-13.404153	-56.12050778	-9.5	-39.7746	-7.1	-29.72628
1MLC: A,B-E	-15.42	-64.560456	-28.8	-120.57984	-1.6	-6.69888
3HFL (1YQV): L,H-Y	-15.0244	-62.90415792	-18.2	-76.19976	-2.3	-9.62964
3HFM: L,H-Y	-14.95	-62.59266	-22.3	-93.36564	-3.9	-16.32852
1FBI: P,Q-Y	-15.990846	-66.95047403	-24.5	-102.5766	-4.3	-18.00324
1MEL: A-L	-18.63	-78.000084	-7.5	-31.401	1.8	7.53624
1DVF: A,B-C,D	-15.873348	-66.45853341	-17.2	-72.01296	-4.7	-19.67796
1NFD: A,B-E,F	-12.999163	-54.42489565	-103.4	-432.91512	-15.1	-63.22068
1AO7: A-D,E	-10.664	-44.6480352	-12.7	-53.17236	2.4	10.04832
1JEL: L,H-P	-12.51	-52.376868	-27.5	-115.137	-1.4	-5.86152
1NCA: L,H-N	-16.71709	-69.99111241	-33.3	-139.42044	-1.9	-7.95492
1NMB: L,H-N	-9.9028	-41.46104304	-16	-66.9888	-1.2	-5.02416
1NSN: L,H-S	-11.809297	-49.44316468	-24.7	-103.41396	-6	-25.1208
1OSP: L,H-O	-12.906794	-54.03816512	-21.2	-88.76016	-9.7	-40.61196
1QFU: L,H-A,B	-13.80136	-57.78353405	-72.1	-301.86828	1.9	7.95492
1IAI: L,H-M,I	-16.073111	-67.29490113	-66.6	-278.84088	-3	-12.5604
1KB5: L,H-A,B	-17.861485	-74.7824654	-36.4	-152.39952	-0.1	-0.41868
2PCC: A,C-B,D	-17.627	-73.8007236	-1.2	-5.02416	-8.3	-34.75044
1GLA: F-G	-11.56514	-48.42092815	-10.9	-45.63612	3.2	13.39776
1BRS: B-E	-14.946989	-62.58005355	0	0	-3	-12.5604
1UDI: E-I	-16.966078	-71.03357537	-11.2	-46.89216	5.7	23.86476
1DHK: A-B	-25.35443	-106.1539275	-12.5	-52.335	3.4	14.23512
1FSS: A-B	-17.481443	-73.19130555	-6.1	-25.53948	1.9	7.95492
1YDR: E-I	-17.881904	-74.86795567	-4	-16.7472	5.1	21.35268
1DFJ: E-I	-15.546119	-65.08849103	-4.3	-18.00324	1.3	5.44284
1A0O: E-F	-9.917062	-41.52075518	-3.2	-13.39776	-3.5	-14.6538
1GUA: A-B	-10.229473	-42.82875756	-4.2	-17.58456	2.2	9.21096
1A2K: A-D	-14.780213	-61.88179579	-6.2	-25.95816	0.6	2.51208
1AGR: D-H	-11.336003	-47.46157736	-1.9	-7.95492	-3	-12.5604
1TX4: A-B	-16.964233	-71.02585072	-11.1	-46.47348	6.7	28.05156
1GG2: A-B	-21.686709	-90.79791324	-8	-33.4944	4	16.7472
1GOT: A-B,G	-22.746217	-95.23386134	-8.6	-36.00648	4.8	20.09664
2TRC: B,G-P	-33.354306	-139.6478084	-53.5	-223.9938	16.8	70.33824
1FIN: A-B	-27.222807	-113.9764483	-14	-58.6152	10.3	43.12404
1AIP: A-C	-13.917887	-58.27140929	-9.4	-39.35592	3	12.5604
1EFU: C-D	-24.17918	-101.2333908	-23.7	-99.22716	23.4	97.97112
1AK4: B-C	-11.110347	-46.51680082	-3	-12.5604	0	0
1IGC: L,H-A	-11.18811	-46.84237895	-26.6	-111.36888	-2.2	-9.21096
1EFN: A-B	-14.047	-58.8119796	-6.5	-27.2142	1.7	7.11756
1FC2: C-D	-13.815379	-57.8422288	-6.1	-25.53948	-1.8	-7.53624
1SEB: A,B-C,D	-13.693108	-57.33030457	-40.6	-169.98408	-1.8	-7.53624

**1ATN: A-D**	-13.294451	-55.66120745	-9.8	-41.03064	3.8	15.90984
**1YCS: A-B**	-12.1254	-50.76662472	-2.8	-11.72304	-1.2	-5.02416
**2BTF: A-P**	-16.8	-70.33824	-6.5	-27.2142	1.1	4.60548
**1HWG: A-B,C**	-28.595811	-119.7249415	-21.9	-91.69092	8.4	35.16912
**1DKG: A,B-D**	-10.6749	-44.69367132	-59.5	-249.1146	-1.3	-5.44284
