# Supplementary material for: Computational Structural Analysis: Multiple Proteins Bound to DNA
Source: PLoS One. 2008 Sep 19;3(9):e3243. doi: 10.1371/journal.pone.0003243 (PMC2532747; doi:10.1371/journal.pone.0003243)
Supplement: Table S31 — Average solvation energy (kJ/mol), free energy barrier of assembly dissociation (kJ/mol), and energy Z-scores for direct and indirect readouts for groups -SubSetMultiProteins∶DNA, -SingleSameProtein∶DNA (0.03 MB DOC) [file pone.0003243.s038.doc]

**Table S31.** Average solvation energy $\Delta G^{\mathrm{int}}$ (kJ/mol), free energy barrier of assembly dissociation $\Delta G^{diss}$ (kJ/mol), and energy Z-scores for direct and indirect readouts for groups –SubSetMultiProteins:DNA, -SingleSameProtein:DNA

| Dataset of complexes | Average (± SE) solvation energy $\Delta G^{\mathrm{int}}$ (kJ/mol) | Average (± SE) $\Delta G^{diss}$ (kJ/mol) | Average (± SE) energy Z-score for direct readout | Average (± SE)energy Z-score for indirect readout |
| --- | --- | --- | --- | --- |
| Group-SubSetMultiProteins:DNA | -241.18±22.3 | 39.38±5.4 | -2.86±0.3 | -2.43±0.2 |
| Group-SingleSameProtein:DNA | -99.79±15.0  (p<0.001) | 31.06±6.5  (p=0.16) | -1.34±0.3  (p<0.001) | -1.48±0.3  (p=0.008) |

p-values are calculated in comparison with Group A and obtained using the one-tailed Student’s t-test
