# Supplementary material for: Computational Structural Analysis: Multiple Proteins Bound to DNA
Source: PLoS One. 2008 Sep 19;3(9):e3243. doi: 10.1371/journal.pone.0003243 (PMC2532747; doi:10.1371/journal.pone.0003243)
Supplement: Table S32 — Average protein-DNA energy binding affinity (kJ/mol), interface overlapping volume (Å3) and average number of interface collision atoms for groups -SubSetMultiProteins∶DNA, -SingleSameProtein∶DNA (0.03 MB DOC) [file pone.0003243.s039.doc]

**Table S32.** Average protein-DNA energy binding affinity (kJ/mol), interface overlapping volume (Å3) and average number of interface collision atoms for groups –SubSetMultiProteins:DNA, -SingleSameProtein:DNA

| Dataset of complexes | Average (± SE) protein-DNA energy binding affinity (kJ/mol) | Average (± SE) protein-DNA  overlapping volume (Å^3^) | Average (± SE) number of atoms in collision in protein-DNA interfaces |
| --- | --- | --- | --- |
| Group-SubSetMultiProteins:DNA | -41.99±1.4 | 4.32±0.8 | 33.2±3.4 |
| Group-SingleSameProtein:DNA | -32.79± 0.9(p<0.001) ^#^ | 2.313±0.8 (p=0.04) | 15.5±3.3 (p=0.001) |

p-values are calculated in comparison with Group A and obtained using the one-tailed Student’s t-test

# unequal variance
