# Supplementary material for: Computational Structural Analysis: Multiple Proteins Bound to DNA
Source: PLoS One. 2008 Sep 19;3(9):e3243. doi: 10.1371/journal.pone.0003243 (PMC2532747; doi:10.1371/journal.pone.0003243)
Supplement: Table S33 — List of PDB IDs used in the study (group-MultiProteins∶DNA), with description of component (including Swiss Prot ID) and biological process of components. (0.08 MB DOC) [file pone.0003243.s040.doc]

**Table S33. List of PDB IDs used in the study (group-MultiProteins:DNA), with description of component (including Swiss Prot ID) and biological process of components.**

| **PDB ID** | **Components with UniProt ID** | **Biological process** |
| --- | --- | --- |
| 1A02 | Nfat (Q13469), Fos (P01100), Jun (P05412) | Regulation of transcription |
| 1AKH | Mating-type protein A-1 (P01366), mating-type protein Alpha-2 (Q6B2C0) | Regulation of transcription |
| 1AWC | GA binding protein alpha (Q00422), GA binding protein beta-1 (Q00420) | Regulation of transcription |
| 1B72 | Homeobox protein HOX-B1 (P14653), PBX1 (P40424) | Regulation of transcription |
| 1B8I | Ultrabithorax homeotic protein (P83949), homeobox protein extradenticle (P40427) | Regulation of transcription |
| 1CF7 | E2F-4 (Q16254), DP-2 (Q14188) | Regulation of transcription, regulation of progression through cell cycle |
| 1CQT | TF1 (P14859), association factor 1 (Q16633) | Regulation of transcription |
| 1D3U | TATA-binding protein (P62001), TF IIB (P61999) | Regulation of transcription |
| 1DSZ | Retinoic acid receptor alpha (P10276), retinoic acid receptor RXR-alpha (P19793) | Regulation of transcription |
| 1FOS | c-Jun (P05411), c-Fos (P01100) | Regulation of transcription |
| 1GT0 | Octamer binding TF-1 (P14859), SOX-2 (P48432) | Regulation of transcription |
| 1H8A | CAAT/ENHANCER binding protein beta (P17676), MYB transforming protein (P01104) | Regulation of transcription |
| 1H9D | Core-binding factor alpha subunit 1 (Q01196), core-binding factor cbf-beta (Q13951) | Regulation of transcription |
| 1HBX | Serum response factor (P11831), Ets-domain protein ELK-4 (P28324) | Regulation of transcription |
| 1HJB | CAAT/ENHANCER binding protein beta (P17676), RUNT-related TF (Q03347) | Regulation of transcription |
| 1IO4 | CAAT/ENHANCER binding protein beta (P17676), RUNT-related TF (Q03347), core-binding factor beta subunit (Q08024) | Regulation of transcription |
| 1JEY | Ku70 (P12956), Ku86 (P13010) | Double-strand break repair via non-homologous end joining |
| 1JFI | Transcription regulator NC2 (Q14919), TATA-box binding protein (P20226), TATA-binding protein-associated phosphor protein (Q01658) | Regulation of transcription |
| 1K6O | ETS domain protein ELK-4 (P28324), serum response factor (P11831) | Regulation of transcription |
| 1K78 | Pax5 (Q02548), C-Ets1 (P27577) | Regulation of transcription |
| 1LB2 | Catabolite gene activator protein (P0ACJ8), DNA-directed RNA polymerase alpha chain (P0A7Z4/9) | Regulation of transcription |
| 1LE5 | Nuclear factor NF-kappa-B p65 subunit (Q04207), nuclear factor NF-kappa-B p105 factor (P25799) | Regulation of transcription |
| 1LE8 | Mating type protein A-1 (P01366), mating type protein alpha-2 (Q6B2C0) | Regulation of transcription |
| 1MDM | Paired box protein PAX-5 (Q02548), C-ETS-1 protein (P27577) | Regulation of transcritpion |
| 1MNM | MCM1 (P11746), MAT ALPHA-2 (Q6B2C0) | Regulation of transcritpion |
| 1N6J | Myocyte-specific enhancer factor 2B (Q02080), calcineurin-binding protein Cabin 1 (Q9Y6J0) | Regulation of transcription |
| 1NGM | TF IID (P13393), TF IIIB BRF subunit (P29056) | Regulation of transcription |
| 1NH2 | TF IID (P13393), TF IIA (P32773-4) | Regulation of transcription |
| 1NKP | Myc proto-oncogene protein (P01106), Max protein (P61244/5) | Regulation of transcription |
| 1NLW | Mad protein (Q05195), Max protein (P61244/5) | Regulation of transcription |
| 1O4X | OCT-1 (P14859), SOX-2 (P48431) | Regulation of transcription |
| 1OUZ | Integration host factor alpha subunit (P0A6X7), integration host factor beta subunit (P0A6Y1) | DNA binding |
| 1PUF | Homeobox protein Hox-A9 (P09631), pre-B-cell leukemia transcription factor-1 (P40424) | Regulation of transcription |
| 1R0O | Ultraspiracle protein (P20153), ecdysone receptor (P34021) | Regulation of transcription |
| 1RIO | SigA (Q9EZJ8), repressor protein Cl (P03034) | Regulation of transcription |
| 1RZR | Glucose-resistance amylase regulator (P46828), phosphocarrier protein HPr (O69250) | Transport |
| 1T2K | Interferon regulatory factor 3 (Q14653), transcription factor AP-1 (P05412), cyclic-AMP-dependent transcription factor ATF-2 (P15336) | Regulation of transcription |
| 1TQE | Myocyte-specific enhancer factor 2B (Q02080), histone deacetylase 9 (Q99N13) | Regulation of transcription |
| 1X9M | DNA polymerase (P00581), thioredoxin 1 (P0AA25) | DNA replication, glycerol ether metabolic process |
| 1XS9 | Multiple antibiotic resistance protein marA (P0ACH5), DNA-directed RNA polymerase alpha chain (P0A7Z4) | Regulation of transcription |
| 1YNW | Vitamin D3 receptor (P11473), retinoic acid receptor RXR-alpha (P19793) | Nuclear hormone receptor |
| 2AS5 | Nuclear factor of activated T-cells, cytoplasmic 2 (Q13469), Forkhead box protein P2 (Q15409) | Regulation of transcription |
| 2BSQ | Trafficking protein A (Q5F881), trafficking protein B (Q5F882) | - |
| 2F8X | Neurogenic locus notch homolog protein 1 (P46531), mastermind-like protein 1(Q92585), recombining binding protein suppressor of hairless, isoform 4 (Q06330) | Receptor for membrane-bound ligands, regulation of transcription |
| 2FO1 | Lin-12 and glp-1 phenotype protein 1 isoform b (Q8MXE7), protein lag-3 (Q09260), Lin-12 protein (P14585) | Regulation of transcription |
| 2NLL | Retinoic acid receptor (P19793), thyroid hormone receptor (P10828) | Regulation of transcription |
