# Supplementary material for: Computational Structural Analysis: Multiple Proteins Bound to DNA
Source: PLoS One. 2008 Sep 19;3(9):e3243. doi: 10.1371/journal.pone.0003243 (PMC2532747; doi:10.1371/journal.pone.0003243)
Supplement: Table S34 — The list of PDB codes of complexes from group-SingleProtein∶DNA (0.03 MB DOC) [file pone.0003243.s041.doc]

**Table S34.** The list of PDB codes of complexes from group-SingleProtein:DNA

| 1A0A  1A3Q  1AM9  1B01  1B3T  1BDT  1BG1  1BL0  1BPY  1C8C  1CEZ  1CKT  1CL8  1CW0 | 1D02  1DC1  1DDN  1DEW  1DFM  1DH3  1DIZ  1DMU  1DP7  1E3O  1ECR  1EFA  1EGW  1ESG | 1EWN  1EWQ  1EYG  1F44  1F4K  1FOK  1FZP  1G38  1G9Z  1GDT  1HLV  1HWT  1I3J  1I6J | 1I7D  1IAW  1IC8  1IGN  1J1V  1JB7  1JE8  1JJ4  1JMC  1JT0  1JX4  1K3X  1K4T  1KC6 | 1KDH  1KU7  1L3L  1L3S  1LLM  1LMB  1LQ1  1LRR  1LWY  1M5R 1MHD  1MJO  1MNN  1MUS | 1MW8  1MWI  1ODH  1OE4  1ORN  1OUP  1P4E  1P71  1P7H  1PV4  1QNA  1QPZ  1QRV | 1QUM  1REP  1SKN  1TC3  1TRO  1TUP 1UBD  1VAS  1ZME  2BOP  2CGP  2DRP  2HDD | 2IRF  2PJR  3HTS  3PVI  6CRO  6MHT |
| --- | --- | --- | --- | --- | --- | --- | --- |
