# Supplementary material for: Computational Structural Analysis: Multiple Proteins Bound to DNA
Source: PLoS One. 2008 Sep 19;3(9):e3243. doi: 10.1371/journal.pone.0003243 (PMC2532747; doi:10.1371/journal.pone.0003243)
Supplement: Table S35 — The list of PDB codes of complexes from group-SingleSameProtein∶DNA (0.03 MB DOC) [file pone.0003243.s042.doc]

**Table S35.** The list of PDB codes of complexes from group-SingleSameProtein:DNA

| 1A66  2H7H  1LFU  1TGH  1GU4 | 1BC8  1Y05  2RAM  1K61  1YTB | 1TTU  1PH7  1KB2  1U8B  1KU7 | 1C7U  9ANT |
| --- | --- | --- | --- |
