# Supplementary material for: Computational Structural Analysis: Multiple Proteins Bound to DNA
Source: PLoS One. 2008 Sep 19;3(9):e3243. doi: 10.1371/journal.pone.0003243 (PMC2532747; doi:10.1371/journal.pone.0003243)
Supplement: Table S36 — The list of PDB codes of complexes from group-SubSetMultiProteins∶DNA (0.03 MB DOC) [file pone.0003243.s043.doc]

**Table S36.** The list of PDB codes of complexes from group-SubSetMultiProteins:DNA

| 1A02  1B72  1B8I  1D3U  1H8A | 1HJB  1IO4  1JFI  1K6O  1K78 | 1LE5  1MNM  1PUF  1RIO  1T2K | 1XS9  1YNW  2AS5  2FO1 |
| --- | --- | --- | --- |
